# Structure–property relationships in dicyanopyrazinoquinoxalines and their hydrogen-bonding-capable dihydropyrazinoquinoxalinedione derivatives

**DOI:** 10.3762/bjoc.20.92

**Published:** 2024-05-08

**Authors:** Tural N Akhmedov, Ajeet Kumar, Daken J Starkenburg, Kyle J Chesney, Khalil A Abboud, Novruz G Akhmedov, Jiangeng Xue, Ronald K Castellano

**Affiliations:** 1 Department of Chemistry, University of Florida, PO Box 117200, Gainesville, FL, 32611, United States; 2 Department of Materials Science and Engineering, University of Florida, PO Box 116400, Gainesville, Florida 32611, United States; 3 C. Eugene Bennett Department of Chemistry, West Virginia University, 100 Prospect Street, Morgantown, WV 26506, United States; 4 Department of Chemistry and Biochemistry, The University of Oklahoma, 101 Stephenson Parkway, Norman, OK 73019, United States

**Keywords:** conjugated molecules, *N*-heteroacenes, hydrogen bonding, optoelectronic properties, organic field-effect transistors, organic semiconductors

## Abstract

Presented here is the design, synthesis, and study of a variety of novel hydrogen-bonding-capable π-conjugated *N*-heteroacenes, 1,4-dihydropyrazino[2,3-*b*]quinoxaline-2,3-diones (DPQDs). The DPQDs were accessed from the corresponding weakly hydrogen-bonding dicyanopyrazinoquinoxaline (DCPQ) suspensions with excess potassium hydroxide, resulting in moderate to good yields. Both families of compounds were analyzed by UV–vis and NMR spectroscopy, where the consequences of hydrogen bonding capability could be assessed through the structure–property studies. Conversion of the DCPQs into hydrogen-bonding capable DPQDs results in modulation of frontier MO energies, higher molar extinction coefficients, enhanced crystallinity, and on-average higher thermal stability (where in some cases the 5% weight loss temperature is increased by up to 100 °C). Single crystal X-ray diffraction data could be obtained for three DPQDs. One reveals pairwise hydrogen bonding in the solid state as well as a herringbone packing arrangement rendering it a promising candidate for additional studies in the context of organic optoelectronic devices.

## Introduction

The role of weak intermolecular interactions in tuning the properties of organic semiconductors has garnered significant attention in the past two decades, owing to their profound implications on device performance [1]. Among these interactions, hydrogen bonding (H-bonding, HB) as a highly directional noncovalent interaction can influence the structural, electronic, and optoelectronic properties of bulk materials [2–3]. Hydrogen bonding plays a crucial role in molecular ordering in solid-state organic semiconductors, thereby dictating charge transport pathways as well as carrier mobilities of both electrons and holes [4–6]. Furthermore, strong hydrogen bonding interactions effectively modulate energy levels of semiconducting materials, affecting the bandgap and charge injection/extraction processes [7]. The additional merits of H-bonding designs in organic optoelectronic materials include higher thermal stability, synergistic stabilizing effects with π-stacking interactions, etc. [8].

Acenes and *N*-heteroacenes are two prominent π-conjugated scaffolds for both *n*- and *p*-type organic semiconductors [9–12]. The Würthner and Meijer groups presented an early example of the utility of hydrogen bonding in the fabrication of organic semiconductor devices. A thin-film device architecture was developed consisting of oligo(*p*-phenylenevinylene) (OPV) and perylenebisimide (PBI) components connected laterally through H-bonding and self-assembled orthogonally through π–π interactions [13]. Mixtures of PBI and OPV successfully exhibited ambipolar charge transport depending on processing conditions as a result of the co-assembled morphology.

Our group utilized a supramolecular approach in the solar cell arena. π-Conjugated linear and branched oligothiophenes appended to H-bonding capable phthalhydrazide (PH) were prepared [14–15]. These compounds demonstrated a power conversion efficiency (PCE) twice as high as that of non-hydrogen bonding controls upon photovoltaic device fabrication with the electron acceptor C_60_. Subsequently, the design was extended to “ditopic” systems with diverse HB-capable units such as PH, 2,4-diamino-1,3,5-triazine (DAT), and barbiturate (B) that can form trimeric and hexameric “rosettes”, respectively [16].

**Figure 1:**
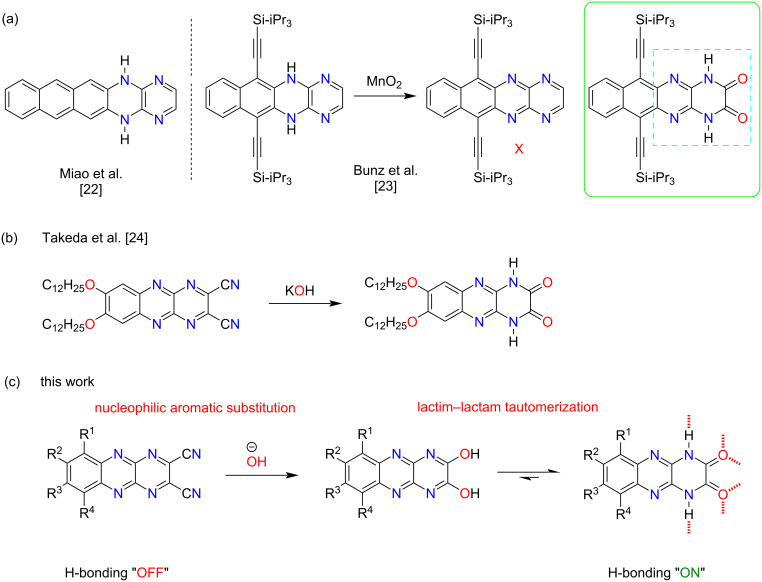
Chemical structures of H-bonding *N*-heteroacenes synthesized by Miao et al. and Bunz et al. (a) [22–23]. Preparation of electron-accepting azaacene derivatives as liquid-crystalline materials by Takeda et al. (b) [24]. Transformation of Yamashita’s DCPQs to H-bonding capable DPQD derivatives discussed in this work (c) [25].

The Sokolowski and Głowacki groups have extensively developed H-bonded systems including dyes and pigments utilized in organic field-effect transistor (OFET) devices [17–20]. In two different studies, Głowacki reported H-bonding capable quinacridone and epindolidione-based semiconductors within OFET devices that offer moderate to excellent hole transport mobilities (1.5 × 10^−3^ and 1.5 cm^2^/Vs, respectively) [21]. In 2012, Miao et al. reported an H-bonding capable 1,4-dihydropyrazinopyrazine fragment within *N*-heteroacenes [22]. The X-ray analysis of single crystals revealed the formation of highly-ordered ribbons constructed via intermolecular N–H∙∙∙N interactions. However, vacuum-deposited films afforded poor hole transport. In separate work, Bunz et al. reported the serendipitous and understated synthesis of an H-bonding capable 1,4-dihydropyrazino[2,3-*b*]quinoxaline-2,3-dione (Figure 1a) [23]. In 2018, Takeda et al. reported the synthesis, electronic characteristics, and liquid-crystalline properties of several electron-accepting acenes, including 1,4-dihydropyrazinoquinoxalinediones (Figure 1b). The incorporation of strong hydrogen-bonding interactions facilitated the formation of a highly ordered liquid-crystalline phase within these systems [24]. These molecules are particularly intriguing as they bear resemblance to Głowacki’s compounds discussed above. Reported here is a robust structure–property relationship study of these types of molecules with a particular interest in their optoelectronic properties.

Primary motivation for our effort comes from the work of Yamashita et al. who explored the OFET behavior of a library of electron-deficient dicyanopyrazinoquinoxaline (DCPQ) compounds [25]. The computations predicted low-lying LUMO levels, around −4.0 eV. However, the molecules exhibited poor *n*-type FET behavior and electron transport characteristics (μ_e_ = 1 × 10^−8^ to 3.6 × 10^−6^ cm^2^/Vs). Structurally, the combination of cyano groups and an electron-deficient pyrazinoquinoxaline creates an extremely electron poor π-system. Given our previous work and that of others, in 2013 we reasoned [26] that DCPQs could be efficiently transformed to tautomerically active, H-bonding capable 1,4-dihydropyrazino[2,3-*b*]quinoxaline-2,3-diones (Figure 1b, DPQDs) via nucleophilic aromatic substitution (S_N_Ar) at the *ipso*-CN positions. Here, the lactim–lactam tautomerization of DPQDs to arrive at the more stable 2,3-dione lactam form would mirror our prior work with phthalhydrazide (PH) and is also consistent with the work later reported by Takeda et al. We additionally envisioned that the DCPQs would serve as valuable comparators as the H-bonding capable DPQDs were studied.

Reported here is the synthesis of a library of dicyanopyrazinoquinoxalines (DCPQs) **1a**–**7a** and subsequent mild one-step synthesis of hydrogen-bonding dihydropyrazinoquinoxalinedione (DPQDs) **1b**–**7b**. The structure–property relationships have been established within and between the two families using optical measurements. Moreover, the incorporation of H-bonding functionality exhibits a noteworthy impact on the thermal stabilities (analyzed by TGA) of the compounds. The single-crystal X-ray diffraction of DPQD **2b** exhibits a desirable herringbone solid-state arrangement for a potential OFET application. Complementary gas-phase computational analysis has provided insight into the electronic structures of both families which is related to the optical properties studied in solution.

## Results and Discussion

### Synthesis of DCPQs and DPQDs

**Scheme 1:**
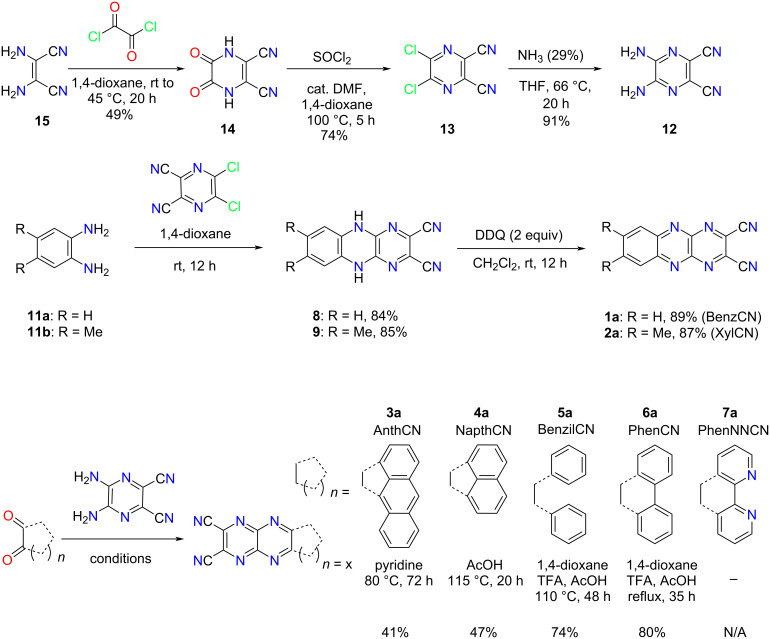
Synthesis of dicyanopyrazinoquinoxaline derivatives **1a**–**7a**.

Compounds **12** and **13** are two major building blocks to obtain dicyanopyrazinoquinoxalines (DCPQs) **1a**–**7a**. The synthesis proceeded through highly scalable reactions (over 10-gram scale) using diaminomaleonitrile (**15**) as a starting material in accordance with the procedure in the literature (Scheme 1) [27]. The first step is the acylation with oxalyl chloride to yield **14** followed by a reaction with SOCl_2_ in the presence of cat*.* DMF to obtain dichloropyrazine **13**. The solvent 1,4-dioxane is crucial to the synthesis and purification in the initial two steps as other solvents such as THF make the purification process more intricate. This sequence is followed by S_N_Ar with ammonia (29% v/v) to obtain building block diaminopyrazine **12**.

The synthesis of Yamashita’s DCPQs **1a** (BenzCN) and **2a** (XyICN) began via S_N_Ar reaction of commercially available *o*-phenylenediamines **11a** and **11b** with building block **13** to afford dihydropyrazine derivatives **8** and **9**, respectively, as precipitates in 1,4-dioxane solution. The reaction generates two equivalents of HCl, adding a slight excess of diamines to sequester released HCl proved to be pivotal in achieving better yields. Oxidation with two equivalents of DDQ provided the comparator DCPQs **1a** and **2a** in 89% and 87% yield, respectively.

The synthesis of DCPQ **3a** was attempted rigorously under different conditions using building blocks 1,2-aceanthrylenedione and **12**, as detailed in Table S1 (Supporting Information File 1). However, an inseparable unknown impurity was observed along with the desired product **3a** in all cases when analyzed by ^1^H NMR. Under strict temperature and time control (80 °C for 72 hours) using 2.5 equiv of 1,2-aceanthrylenedione, the reaction provided pure **3a** product in moderate yield (41%). Subsequent recrystallization with boiling DMSO provided pristine red-brown crystals of **3a**. The synthesis of DCPQ **4a** was simple and proceeded with the condensation of a 1:1 mixture of acenaphthenequinone and **12** under standard boiling acetic acid conditions, as reported in Scheme 1. The crude product could be collected via vacuum filtration, followed by additional purification via recrystallization from boiling DMF to afford golden crystals of **4a** in moderate yields.

In order to access **5a** and **6a**, a modified procedure from the literature was implemented [28]. To synthesize **6a**, the condensation was performed with 9,10-phenanthrenequinone, building block **12** in the presence of glacial CH_3_COOH, trifluoracetic acid, and 1,4-dioxane at reflux. Then, recrystallization from DMF and sublimation under ambient pressure worked well for the purification step to achieve an excellent 80% yield. To our delight, **5a** did not require any additional purification under the same reaction conditions.

**Scheme 2:**
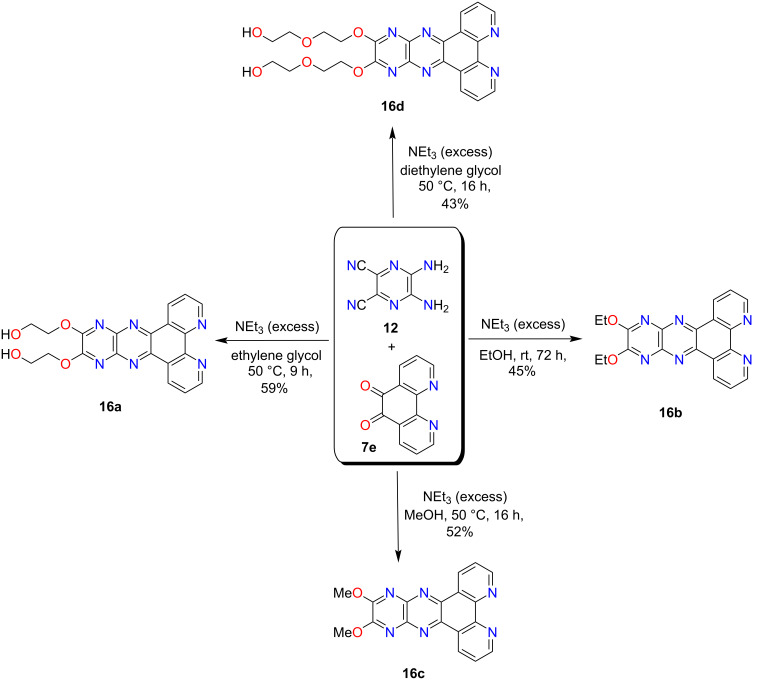
Synthesis of bis-alkoxy-substituted π-conjugated phenanthrolines **16a**, **16b**, **16c**, and **16d**.

**Scheme 3:**
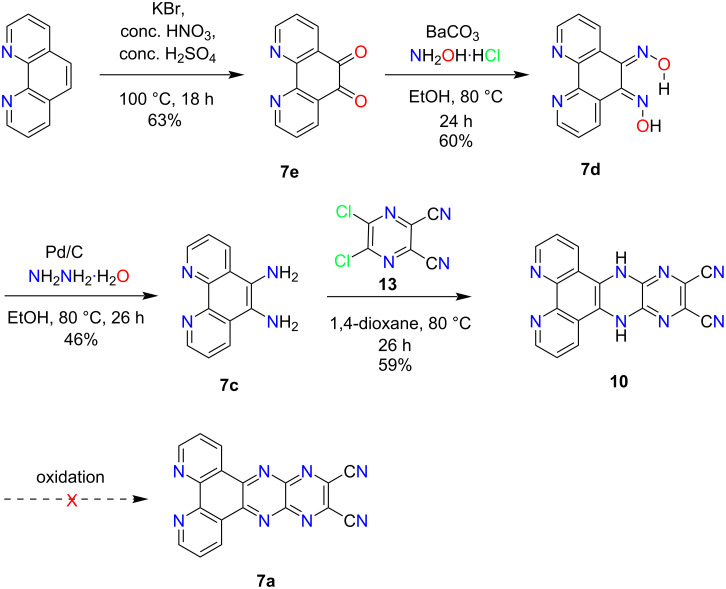
An alternative synthetic route to access **7a**.

Next, several unsuccessful synthetic efforts were made to access **7a** using starting material **7e** and building block **12**, listed in Table S2 (Supporting Information File 1). First, the starting material **7e** was obtained using 1,10-phenanthroline under harsh conditions as shown in Scheme 3 [29]. The same condensation reaction that worked for structurally similar **6a**, produced a poorly separable mixture in the case of **7a**. In the course of optimizing this reaction under different conditions, additional novel products were isolated in moderate yields (Scheme 2, Table S2, Supporting Information File 1). A variety of bis-alkoxylated products **16a**–**d** was obtained in moderate yields upon using different polar solvents such as methanol, ethanol, ethylene glycol, and diethylene glycol in the presence of excess triethylamine (Scheme 2). These products provide evidence for the in situ formation of DCPQ **7a** and demonstrate its ability to undergo trapping with various nucleophiles through an S_N_Ar mechanism.

An alternate strategy was employed to access **7a** as depicted in Scheme 3. The synthetic strategy first involved the access of dihydropyrazine precursor **10** which then underwent oxidation in the final step. Next, **7e** was easily converted to (*Z*,*E*)-bis-oxime derivative **7d** in satisfactory yield. The oxime stereochemistry shown is presumably secured due to the favorable hydrogen bonding (O–H∙∙∙N) between the oxime units; this idea is supported by ^1^H NMR spectroscopy which shows the presence of two distinct hydroxyl proton signals. The reduction of **7d** in the presence of Pd/C and hydrazine monohydrate afforded 1,10-phenanthroline-5,6-diamine (**7c**) in moderate yield. The condensation of **7c** with **13** in the presence of 1,4-dioxane yielded poorly soluble **10**. Unfortunately, several oxidation attempts to access target **7a** failed presumably due to the insolubility of **10** even in high polarity solvents, listed in Table S3 (Supporting Information File 1).

**Scheme 4:**
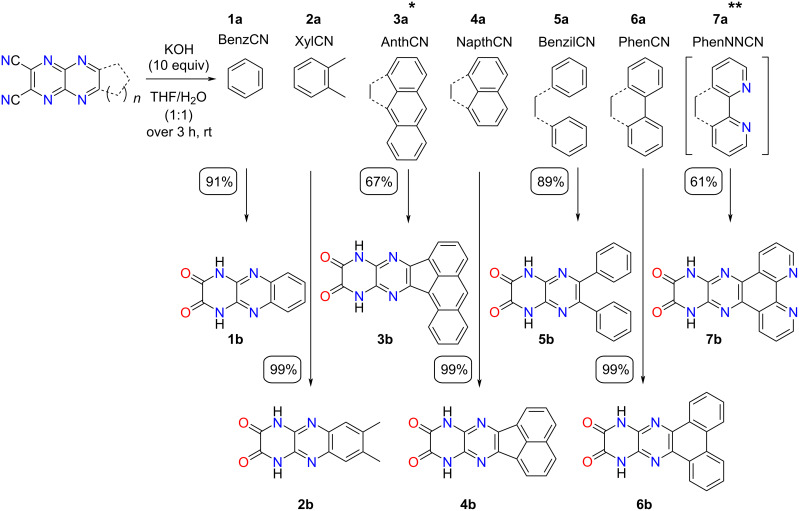
Synthesis of DPQDs **1b**–**7b** from their corresponding DCPQs **1a**–**7a**. *THF/H_2_O/1,4-dioxane (4:5:1). **in situ formation.

The H-bonding capable dihydropyrazinoquinoxaline diones (DPQDs) were obtained by a S_N_Ar mechanism involving the corresponding DCPQ derivatives. Based on numerous examples in the literature, it has been established that for electron-deficient π-systems containing cyano groups, the addition–elimination pathway will dominate over hydrolysis. This preference was crucial to prevent the formation of undesired carboxylic acid products [28,30–31]. These results also align with the observations previously reported by Takeda and co-workers [24]. The reaction was carried out using a large excess (10 equiv) of KOH in a 1:1 THF/H_2_O mixture to improve the solubility of the corresponding DCPQs (Scheme 4) at room temperature. A small amount of 1,4-dioxane (THF/H_2_O/1,4-dioxane 4:5:1) was also added to improve the solubility of **3a**. The solution was rendered neutral by the addition of conc. HCl until precipitation occurred and precipitates were subsequently collected via vacuum filtration with no exception for any DPQDs **1b**–**6b**. The yields obtained in this step were moderate to excellent (61–99%). Target **7b** was synthesized from **12** and **7e** via the in situ generation of **7a** and subsequent S_N_Ar with KOH given the high reactivity of intermediate **7a** (Scheme 4).

After optimizing the successful synthesis of alkoxylated and diketone derivatives of **7a**, we were intrigued to investigate whether other dicyanopyrazinoquinoxaline derivatives could undergo a similar transformation. To assess the efficacy of the reaction, we subjected **6a** to the conditions outlined in Scheme 2. Remarkably, under the same conditions, an isostructural bis-ethoxy derivative, **16e**, was obtained in an excellent yield (87%). In addition, 10 equivalents of ammonium hydroxide yielded mono-aminated derivative **16f** (Scheme S4, Supporting Information File 1). The double substitution did not proceed presumably due to the strong electron-donating effect of -NH_2_, diminishing the electrophilicity of the π-system. However, the mono-aminated product **16f** has the potential to serve as a useful building block for condensation with carbonyl functionality as well as Buchwald–Hartwig amination involving aryl halides.

### Thermal studies

**Figure 2:**
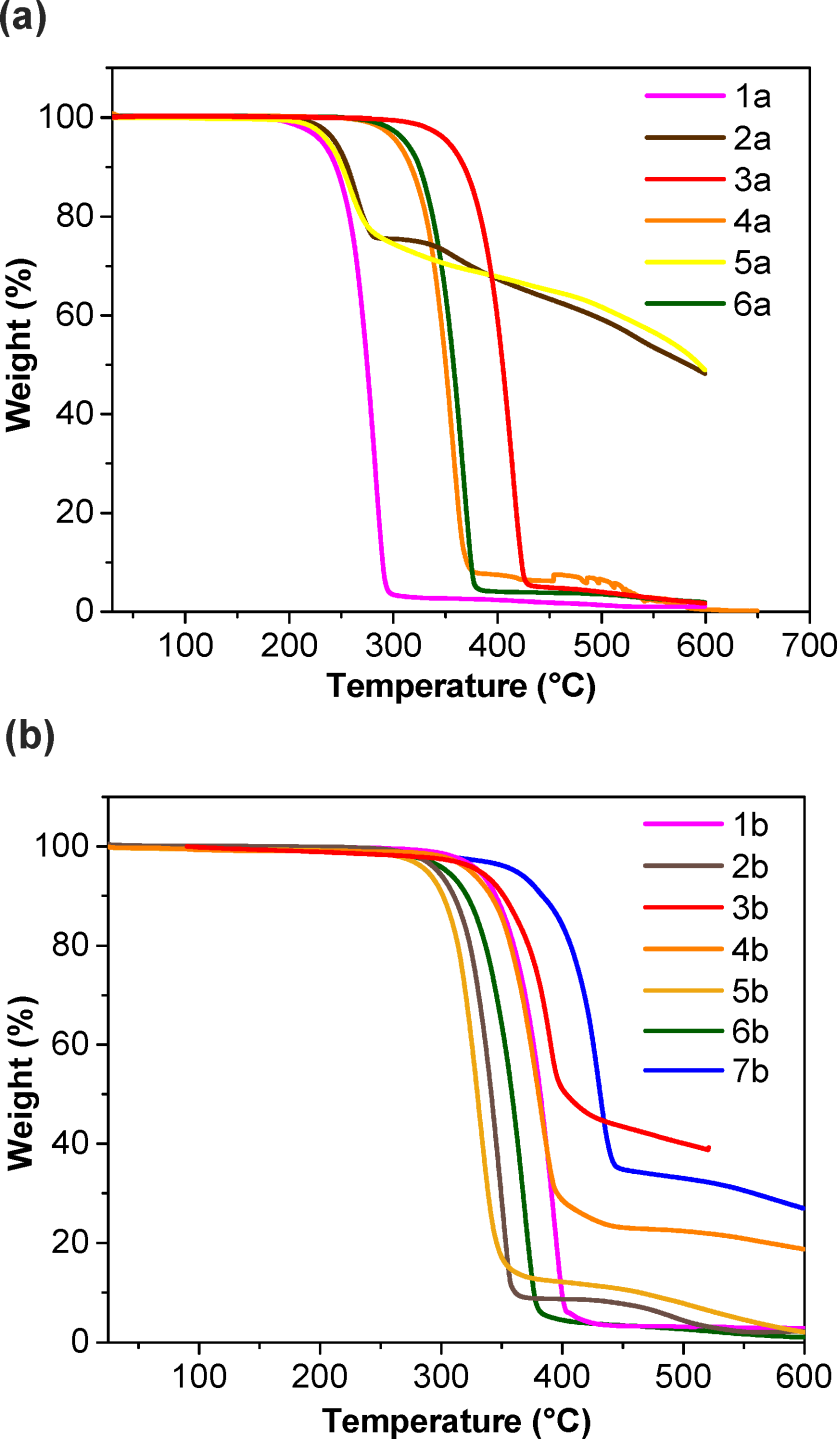
TGA of **1a**–**6a** (a) and **1b**–**7b** (b) obtained at 10 °C/min under nitrogen.

Thermal stabilities of DCPQs **1a**–**6a** and DPQDs **1b**–**7b** were evaluated using thermogravimetric analysis (TGA; Figure 2 and Table S4 in Supporting Information File 1). The thermal stability of the DCPQs is attributed to their favorable π–π stacking in the solid state. The 5% weight loss values range from 232 °C to 353 °C. Compounds with larger π-surfaces exhibited greater thermal stability (353 °C for **3a**, 304 °C for **4a**, 312 °C for **6a**) while others displayed 5% weight loss values under 250 °C (232 °C for **1a**, 244 °C for **2a** and 238 °C for **5a**). It was interesting to observe that there was a large difference in the thermal stabilities of non-planar **5a** and fully planar **6a** (5% weight loss of 238 °C vs 312 °C).

The TGA data for H-bonding DPQDs showed thermal stability with a 5% weight loss ranging between 288 °C and 361 °C. The 5% weight loss value is increased by about 100 °C, particularly in the case of **1b** as compared to the corresponding DCPQ **1a.** This enhancement can be attributed to the synergistic effect of π–π and H-bonding interactions on solid-state cohesive energy. However, **3b** and **7b** exhibited a decrease in 5% weight loss temperatures of 21 and 7 °C, respectively, from their corresponding DCPQ comparators.

To complement the TGA studies, the thermal stability of compound **4b** was evaluated through its exposure to air inside a 200 °C oven for one week. Structural evaluation by 2D NMR and mass spectrometry confirmed no decomposition and its high thermal stability.

### Optical properties

**Figure 3:**
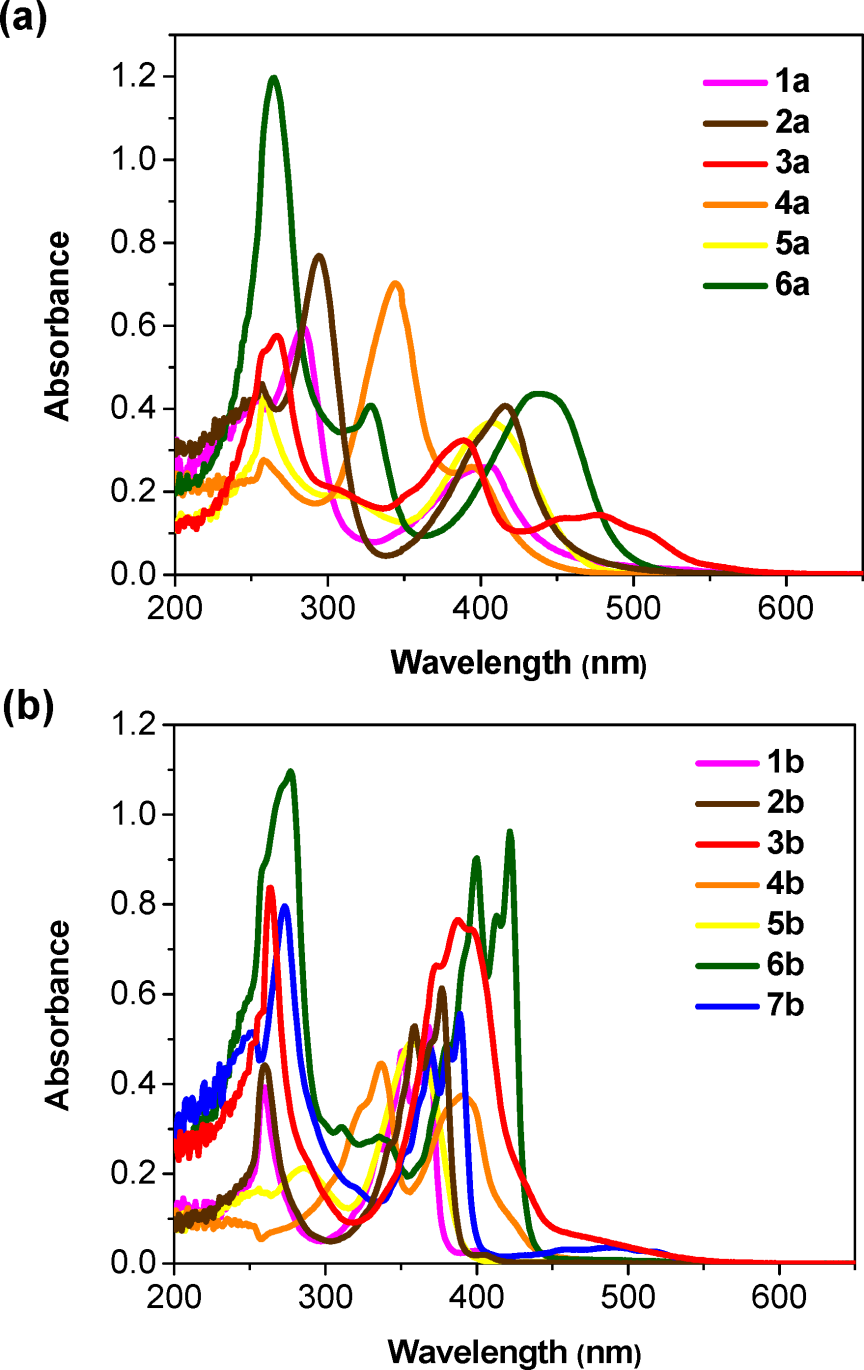
Absorption spectra (20 μM) for a) DCPQs **1a**–**6a** and b) DPQDs **1b**–**7b** in dimethyl sulfoxide.

The electronic properties of the DCPQs **1a**–**6a** and DPQDs **1b**–**7b** were explored by UV–vis absorption spectroscopy in dimethyl sulfoxide (DMSO) at 25 °C (Figure 3). The electronic absorption spectra of DCPQs exhibited structureless bands with λ_max_ values from 388 to 423 nm, indicative of π–π* transitions. On the contrary, DPQDs exhibited more structured, blue-shifted bands with λ_max_ from 357–400 nm. At the same time, DCPQs exhibit intramolecular charge transfer (ICT) bands at lower energy due to the dicyanopyrazinopyrazine moiety as a strong acceptor and aromatic groups as strong donors in the backbone; the dione groups in the case of DPQDs diminish the acceptor properties of the aza π-system. It is worth mentioning that ICT is significantly influenced by solvent polarity and the comprehensive study of solvent effects is not within the scope of this investigation [32].

Also, DPQDs displayed higher molar extinction coefficients (ε) than the corresponding DCPQs by a factor of approximately 1.4 to 5.3 in DMSO (Table 1). Furthermore, in all cases, the compounds from both families adhere to the Beer–Lambert law at the observed concentrations, as illustrated in Figures S37–S41 (Supporting Information File 1). This serves as good evidence that the synthesized compounds exhibit no signs of aggregation in DMSO within the assessed concentration range (i.e., 2.5–45 μM).

### Electronic structure calculations

**Figure 4:**
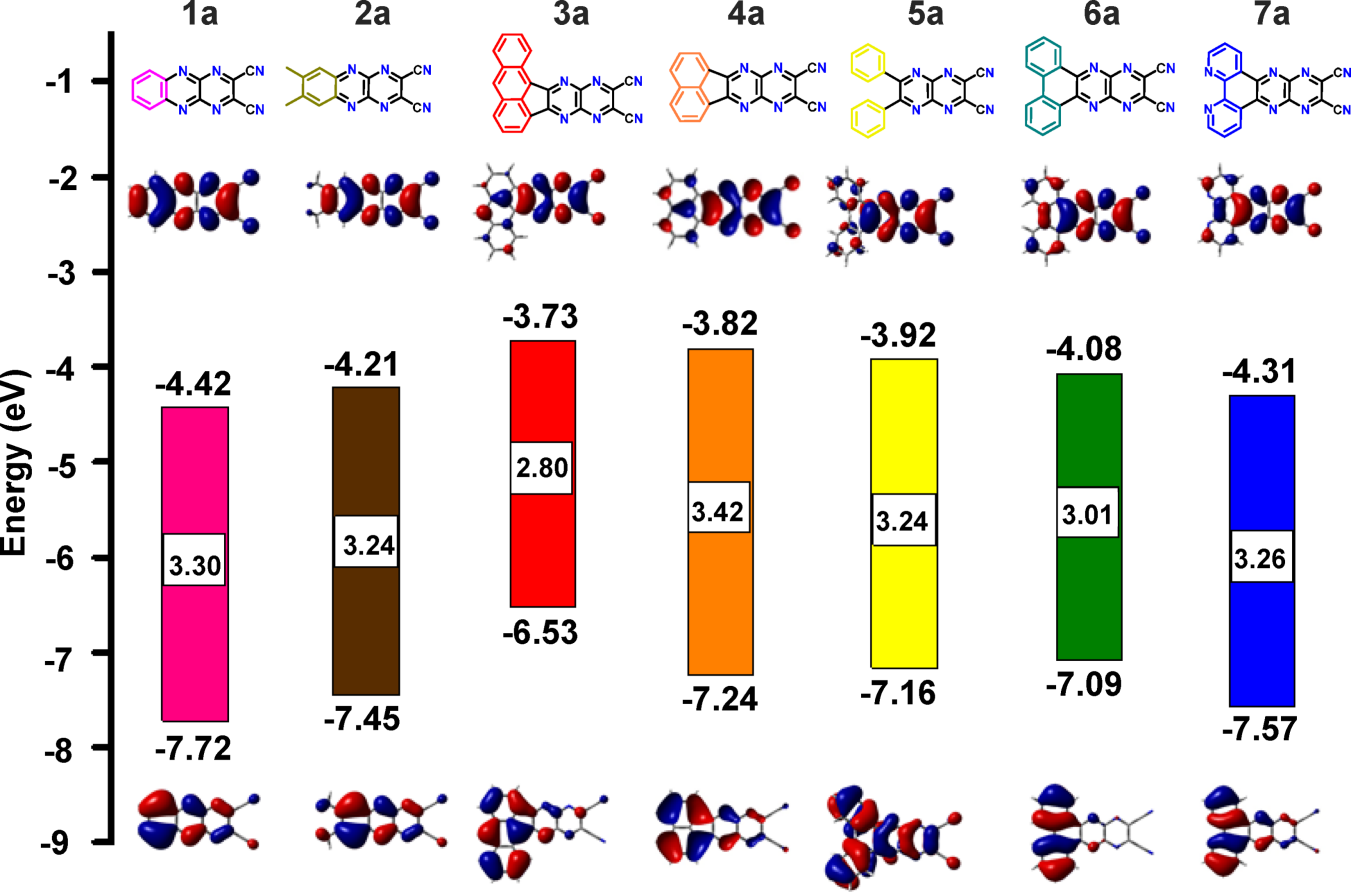
Calculated HOMO (below) and LUMO (above) energies by DFT analysis (B3LYP/6-31+G* level of theory), and corresponding molecular orbital plots for **1a**–**7a**.

**Figure 5:**
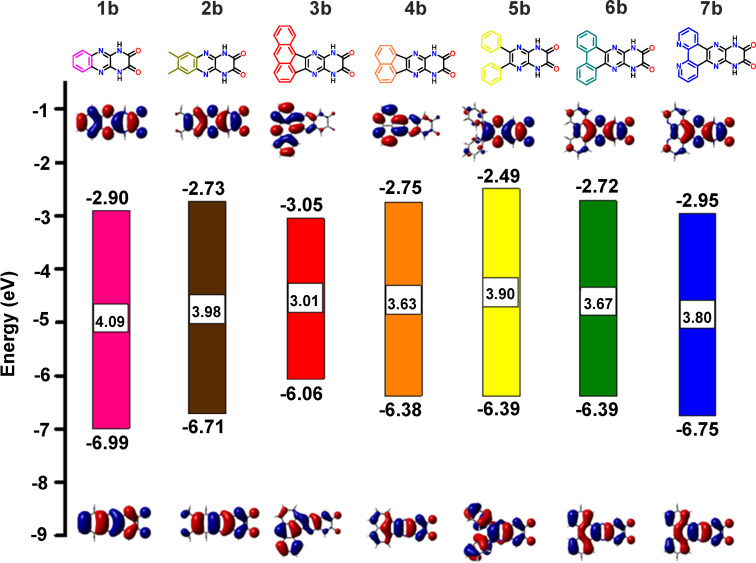
Calculated HOMO (below) and LUMO (above) energies by DFT analysis (B3LYP/6-31+G* level of theory), and corresponding molecular orbital plots for **1b**–**7b**.

Gas-phase computational investigations were conducted to estimate energies of the frontier molecular orbitals (FMOs), specifically the highest occupied molecular orbital (HOMO) and the lowest unoccupied molecular orbital (LUMO), for DCPQs **1a**–**7a** as well as **1b**–**7b**. The density functional theory (DFT) calculations were performed using the B3LYP/6-31+G* level of theory (depicted in Figure 4 and Figure 5). Figure 4 reveals that the HOMOs of **1a**–**7a** are predominantly localized on the π-donor units, such as benzene and naphthalene, with an illustration in the case of **6a**. On the contrary, the LUMOs are delocalized across the entire π-systems, while also displaying some localization on the dicyanopyrazinopyrazine acceptor. Notably, compound **3a** displays the highest orbital density separation between donors and acceptors – an attribute relevant to efficient intramolecular charge transfer processes. This aligns with the observed lower optical HOMO–LUMO gap for **3a** in comparison to other compounds within the DCPQ family of compounds. As shown in Figure 5 and Table 1, the donor–acceptor character of the H-bonding counterparts is considerably muted and the HOMO and LUMO in these cases are both relatively delocalized. The HOMO energies of DPQD **1b**–**7b** are also deep (<−6.0 eV).

**Table 1:** Absorbance properties of **1a**–**6a** and **1b**–**7b** in DMSO.^a^

Compound	λ_max_ (nm)	λ_onset_ (nm)	ε (10^4^ M^−1^ cm^−1^)	*E*_HOMO_^DFT^ (eV)^b^	*E*_LUMO_^DFT^ (eV)^b^	*E*_gap_^DFT^ (eV)^b^	*E*_gap_^opt^ (eV)^a^

**1a**	400	447	1.4	−7.72	−4.42	3.30	2.85
**2a**	400	455	2.1	−7.45	−4.21	3.24	2.71
**3a**	388	546	0.73	−6.53	−3.73	2.80	2.26
**4a**	394	436	1.3	−7.24	−3.82	3.42	2.85
**5a**	407	466	1.7	−7.16	−3.92	3.24	2.67
**6a**	423	492	2.2	−7.09	−4.08	3.01	2.52
**1b**	368	377	2.6	−6.99	−2.89	4.10	3.23
**2b**	377	388	2.8	−6.71	−2.72	3.98	3.20
**3b**	399	451	3.9	−6.06	−3.05	3.01	2.75
**4b**	391	443	1.8	−6.38	−2.75	3.63	2.80
**5b**	357	391	2.4	−6.39	−2.49	3.89	3.17
**6b**	400	432	4.6	−6.39	−2.72	3.67	2.86
**7b**	396	398	3.0	−6.75	−2.95	3.80	3.11

^a^All measurements performed at room temperature and 20 μM concentration; ^b^Energy values obtained from gas-phase computations using Gaussian 09 at the DFT B3LYP-6-31+G* level.

FMO energies are of great importance not only for the stability but also for device optimization. Raising the HOMO energy level can facilitate hole injection when using Pt electrodes [33], consequently enabling the fabrication of ambipolar devices supporting both hole and electron transport. On the other hand, unipolar electron transport could be facilitated in the case of DCPQs **1a**–**6a** given their extremely deep HOMO energy levels. Deep HOMO levels are important for optimal device performance and resistance to oxidation [34]. Compounds with deep LUMO energy levels are essential for efficient electron transport. However, compounds with LUMO levels ≈−4.0 eV are prone to undergo oxidation with O_2_ or reduction with H_2_O [35]. This could be the reason why electron transport properties in the OFETs previously reported by Yamashita are not very good [25]. Therefore, in the case of DPQD derivatives **1b**–**7b**, a balance is achieved, characterized by a HOMO with an energy level below –6.0 eV and moderately low LUMO with an energy level below −2.7 eV. The notable computed HOMO–LUMO difference between DCPQs and DPQDs amounting to more than 1.0 eV in certain cases, correlates well with optical gaps obtained from absorbance spectra, taken as the photon energy corresponding to the cut-off wavelength (Table 1). Subsequently, preliminary electrochemical studies were performed with selected DCPQs and corresponding DPQD counterparts using cyclic voltammetry (Figure S1, Supporting Information File 1). Cyclic voltammetric measurements of DCPQs, specifically **4a** and **6a**, and DPQDs **4b** and **6b**, support the overall trends observed through the electronic structure calculations.

### X-ray crystallography

**Figure 6:**
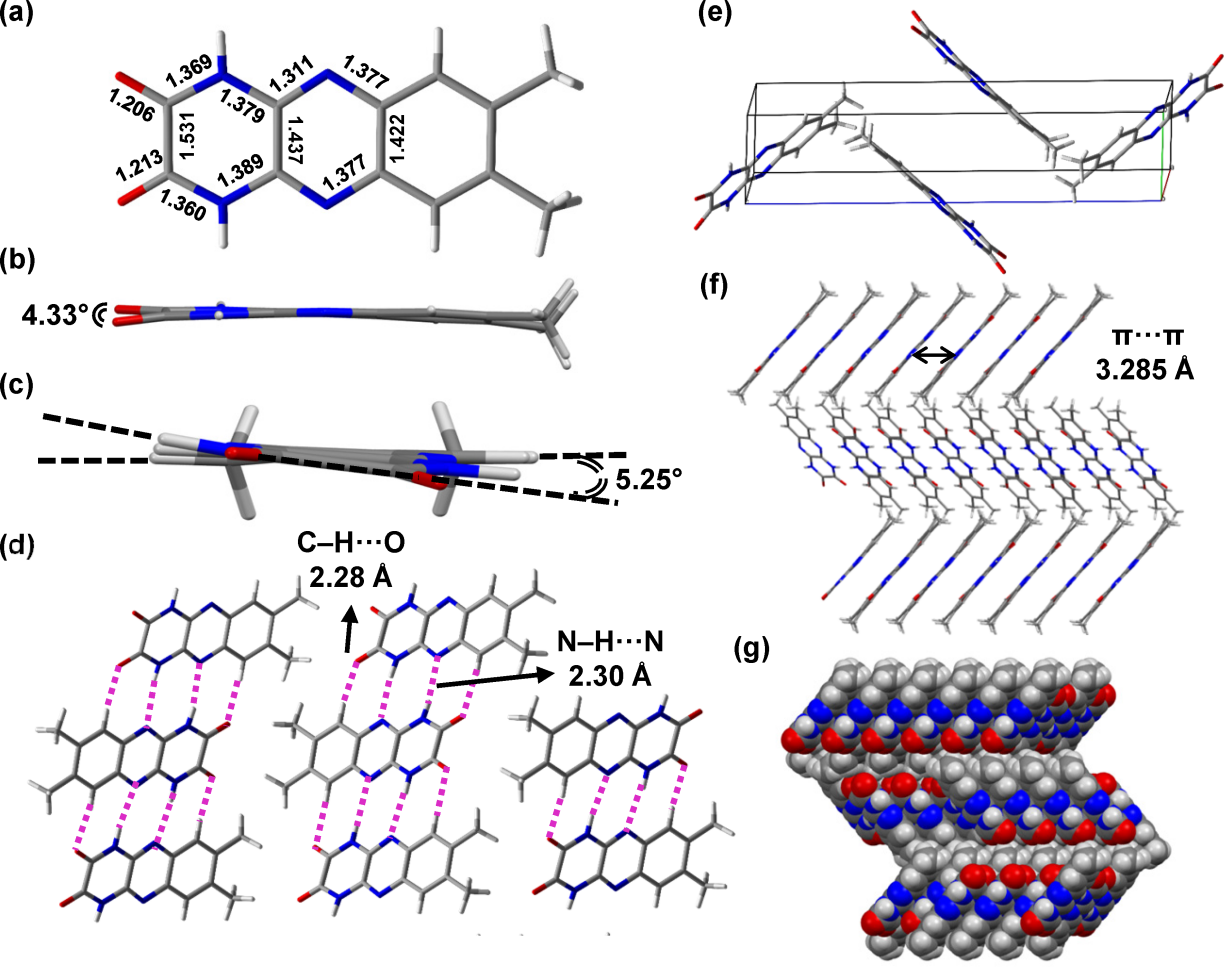
Asymmetric unit of DPQD **2b** with important bond lengths highlighted (a). Torsion angles of 4.33° and 5.25° are associated with the carbonyl groups and rings 1 and 3, respectively (b) and (c). Packing diagram showing the average intermolecular N–H∙∙∙N and C–H∙∙∙O=C distances (d). Unit cell comprised of four molecules of **2b** (e). Herringbone packing of **2b** showing the distance between adjacent ring centroids (f). Herringbone backing of **2b** shown as a spacefilling model (g). Atom color code: blue N, gray C, red O, and white H.

Single crystal X-ray diffraction data could be obtained for **2b**, **5b**, and **6b**. Figure 6 illustrates the single-crystal X-ray analysis of **2b**, and its important structural parameters were compared with that of **2a** (#CCDC 227464) reported in the literature [25]. An improved 2D nanostructure through highly ordered organization facilitated by H-bonding is revealed. Notably, the C–C distance between two carbonyl groups measures 1.531 Å, comparatively larger than that of aromatic systems and on the order of C_sp_^3^–C_sp_^3^ bonds (Figure 6a). This represents the first evidence that the keto form is largely favored. Next, the molecule does not adopt a fully planar conformation, instead exhibiting a torsional angle of 4.33° with respect to the carbonyl groups (Figure 6b) and 5.25° concerning peripheral rings 1 and 3 (Figure 6c). The unit cell is composed of a dimer of **2b** held together by intermolecular N–H∙∙∙N and C–H∙∙∙O=C H-bonds with average distances of 2.30 Å and 2.28 Å, respectively (Figure 6d and 6e). The herringbone packing of **2b** is expected to be favorable for charge mobility within stacked arrangements [36–37]. Additionally, the intermolecular π–π stacking distance is reduced to 3.285 Å in the staggered form (Figure 6f and 6g), 0.27 Å shorter than its non-hydrogen bonding dicyano comparator **2a**. The single-crystal X-ray analysis of other DPQDs **5b** and **6b** did not reveal a herringbone lattice pattern, as shown in Tables S5 and S6 and Figures S42–S45 (Supporting Information File 1). In the case of **5b**, this is presumably due to the twisted phenyl rings that dictate the packing arrangement. Nonetheless, close π-stacking distances (≈3.2 Å) are observed between the **5b** units. For **6b**, this is likely due to the included solvent (ethylene glycol) that hydrogen bonds to the quinoxalinedione. Moving forward, **2b** stands out as a promising candidate for future solid-state characterization and device fabrication studies.

## Conclusion

To conclude, we have used an efficient synthetic method to produce a structurally diverse class of H-bonding capable electron-deficient *N*-heteroacenes DPQDs **1b**–**7b**. The DPQDs were accessed from their respective *N*-heteroacenes DCPQs **1a**–**7a** in one step. Structure–property relationships for the DCPQs and DPQDs were established using gas-phase computational DFT analysis (B3LYP/6-31+G* level of theory), UV–visible spectroscopy, thermal analysis, and X-ray analysis in the solid state for **2b**, **5b**, and **6b**.

The primary takeaways from the work concern the attractive functional consequences of efficiently converting the DCPQs into hydrogen-bonding capable DPQDs. These include modulation of frontier MO energies, higher molar extinction coefficients, and on-average higher thermal stability (where in some cases the 5% weight loss temperature increased by up to 100 °C). Single-crystal X-ray analysis of DPQD derivative **2b** further revealed that it adopts a favorable herringbone motif characterized by short π–π distances and extensive intermolecular hydrogen bonding. These structural features are anticipated to promote efficient charge transport. Future work will involve device fabrication and comparison of the DCPQs and DPQDs in this context. Overall, this work contributes to our overarching objective to investigate the influence of hydrogen-bonding functionality on optoelectronic material organization and next-generation organic semiconductor device performance.

## Experimental

### General methods

Reagents and solvents were purchased from commercial sources and used without further purification unless otherwise specified. THF, ether, CH_2_Cl_2_, and DMF were degassed in 20 L drums and passed through two sequential purification columns (activated alumina; molecular sieves for DMF) under a positive argon atmosphere. Palladium(II) acetate and anhydrous 1,4-dioxane were purchased from Strem Chemicals or Sigma-Aldrich and used as received. Thin-layer chromatography (TLC) was performed on SiO_2_-60 F_254_ aluminum plates with visualization by UV light or staining. Flash column chromatography was performed using Purasil SiO_2_-60, 230−400 mesh from Whatman. Routine 300(75) MHz ^1^H(^13^C) NMR spectra were recorded on Varian Mercury 300 or Gemini 300 spectrometers. Routine 500(125) MHz ^1^H(^13^C) NMR were recorded on an INOVA 500 spectrometer. 2D NMR spectra were recorded using a 600 MHz Varian instrument. Chemical shifts (δ) are given in parts per million (ppm) relative to TMS and referenced to residual protonated solvent (CDCl_3_: δ_H_ 7.26 ppm, δ_C_ 77.23 ppm; DMSO-*d*_6_: δ_H_ 2.50 ppm, δ_C_ 39.50 ppm). Abbreviations used are s (singlet), d (doublet), t (triplet), q (quartet), quin (quintet), hp (heptet), b (broad), and m (multiplet). ESI- and ESI-TOF-MS spectra were recorded on a Bruker APEX II FTICR and Agilent 6210 TOF spectrometer, respectively. EI-, CI-, and DIP-CI-MS spectra were recorded on a Thermo Trace GC DSQ (single quadrupole) spectrometer.

### Absorbance measurements

Absorbance spectra were measured for 2.5, 5, 10, 15, 20, and 40 μM solutions of the DCPQs and DPQDs on a Cary 100 Bio spectrophotometer using 1 cm quartz cells. All solvents were HPLC grade (purchased from Fisher) and stored over 4 Å molecular sieves. The absorption intensity at λ_max_ was then plotted against the concentration in all cases to confirm, by linearity, that the compounds followed Beer’s law. Molar extinction coefficients (ε) were determined from the linear plot for each compound (where *A* = ε*bc*).

### Computational analysis

Starting geometries were obtained from semi-empirical calculations using the MM2 method as implemented in Chem3D Pro v. 13.0.0.3015 for Windows. The ground state geometries, energies and orbital energies were then obtained from DFT calculations at the B3LYP/6-31+G* level as implemented in Gaussian 09 [38], accessed through the UF High-Performance Computing Center. Frequency calculations were performed at the same computational level, and no imaginary frequencies were found. Molecular orbital plots were made using GaussView v. 5.0.898 from the Gaussian output files.

### Thermogravimetric analysis

Thermal gravimetric analysis (TGA) was performed on a TA Instruments TGA Q5000-0121 V3.8 Build 256 at a heating rate of 10 °C/min using 1–3 mg of sample in a 100 μL platinum pan (under nitrogen). The data was analyzed on Universal Analysis 2000 4.4A software.

### Synthetic procedures to access dicyanopyrazinoquinoxalines (DCPQs) **1a**–**6a**

#### Pyrazino[2,3-*b*]quinoxaline-2,3-dicarbonitrile (**1a**)

To a 50 mL round-bottom flask was added DDQ (0.581 g, 2.56 mmol) and CH_2_Cl_2_ (20 mL). To the suspension was added 5,10-dihydropyrazino[2,3-*b*]quinoxaline-2,3-dicarbonitrile (**8**, 0.300 g, 1.28 mmol) in small portions. The reaction was allowed to proceed overnight at room temperature, after which a pale orange reaction mixture was filtered, washed with copious amounts of CH_2_Cl_2_ and THF (10 mL), and dried in vacuo to afford a bright orange solid of **1a** (0.264 g, 1.14 mmol, 89%). ^1^H NMR (600 MHz, DMSO-*d*_6_) δ 8.30–8.31 (dd, *J* = 3 Hz, *J* = 6.6 Hz, 2H), 8.49–8.51 (dd, *J* = 3 Hz, *J* = 6.6 Hz, 2H); ^13^C NMR (150 MHz, DMSO-*d*_6_) δ 114.5, 130.4, 136.1, 136.2, 142.7, 147.4; HRMS (DART): [M + H]^+^ calcd for C_12_H_4_N_6_, 233.0570; found, 233.0574; [M + NH_4_]^+^, 250.0836; found, 250.0839.

#### 7,8-Dimethylpyrazino[2,3-*b*]quinoxaline-2,3-dicarbonitrile (**2a**)

Compound **2a** was synthesized the same way as **1a** using DDQ (0.581 g, 2.56 mmol), 7,8-dimethyl-5,10-dihydropyrazino[2,3-*b*]quinoxaline-2,3-dicarbonitrile (**9**, 0.300 g, 1.28 mmol) and CH_2_Cl_2_ (20 mL). The reaction mixture was filtered, washed with copious amounts of CH_2_Cl_2_, THF (20 mL), and dried in vacuo to give a bright orange solid of **2a** (0.26 g, 1.0 mmol, 87%). ^1^H NMR (500 MHz, CDCl_3_) δ 2.70 (s, 6H), 8.21 (s, 2H); ^1^H NMR (500 MHz, DMSO-*d*_6_) δ 2.66 (s, 6H), 8.27 (s, 2H); ^13^C NMR (125 MHz, DMSO-*d*_6_) δ 20.1, 114.2, 127.8, 134.6, 142.3, 146.6, 148.7. The proton NMR in chloroform matches the literature value [25]; HRMS (DART): [M + H]^+^ calcd for C_14_H_8_N_6_, 261.0883; found, 261.0889; [M + NH_4_]^+^, 278.1149; found, 278.1154.

#### Aceanthryleno[1,2-*b*]pyrazino[2,3-*e*]pyrazine-11,12-dicarbonitrile (**3a**)

An oven dried sealed tube was charged with 5,6-diaminopyrazine-2,3-dicarbonitrile (**12**) (56.6 mg, 0.353 mmol), 1,2-aceanthrylenedione (56.6 mg, 0.244 mmol) and dry pyridine (3 mL). The resulting orange suspension was heated to 80 °C for 72 hours. After this time, brown solids precipitated out. The tube was adequately cooled, and the solid residue was filtered and washed with copious amounts of methanol, ethyl acetate and dichloromethane, to afford pure red-brown colored amorphous material (36 mg, 0.10 mmol, 41%). The material could be further purified by recrystallization from boiling DMSO to obtain highly insoluble red-brown microcrystalline material. ^1^H and ^13^C NMR could not be recorded for the compound **3a** given its extremely poor solubility in common organic solvents. *R*_f_ (CH_2_Cl_2_): 0.75, fluorescent orange spot; HRMS (DART): [M + NH_4_]^+^ calcd for C_22_H_8_N_6_, 374.1154; found, 374.1155.

#### Acenaphtho[1,2-*b*]pyrazino[2,3-*e*]pyrazine-9,10-dicarbonitrile (**4a**)

An oven dried sealed tube was charged with 5,6-diaminopyrazine-2,3-dicarbonitrile (**12**) (0.15 g, 0.92 mmol), acenaphthylene-1,2-dione (0.15 g, 0.84 mmol) and glacial acetic acid (20 mL). The resulting pale-yellow suspension was heated to 115 °C for 20 hours with vigorous stirring. After 20 hours, the reaction was cooled, and yellow-gold shimmering particles began to settle out. The mixture was filtered, and the resulting gold-colored solid was recrystallized from hot DMF to obtain pure compound **4a** (0.12 g, 0.39 mmol, 47%). ^1^H NMR (500 MHz, CDCl_3_) δ 8.04–8.06 (t, *J* = 7.5 Hz, 2H), 8.40–8.42 (d, *J* = 8.5 Hz, 2H), 8.71–8.73 (d, *J* = 7 Hz, 2H); ^13^C NMR could not be obtained due to the poor solubility of the compound; HRMS (DART): [M + NH_4_]^+^ calcd for C_18_H_6_N_6_, 324.0997; found, 324.0978.

#### 6,7-Diphenylpyrazino[2,3-*b*]pyrazine-2,3-dicarbonitrile (**5a**)

Compound **5a** was synthesized by a modified literature method [31]. To an oven dried sealed tube was added 5,6-diaminopyrazine-2,3-dicarbonitrile (**12**, 306 mg, 1.91 mmol), diphenylethanedione (benzil) (365 mg, 1.74 mmol), glacial acetic acid (7 mL), trifluoroacetic acid (1 mL), and 1,4-dioxane (2 mL)*.* The resulting orange suspension was heated at 110 °C for 48 hours. After completion, the tube was allowed to cool to room temperature and placed in a freezer overnight. Gold-colored crystals of **5a** were collected by filtration and dried in vacuo (430 mg, 1.28 mmol, 74%). ^1^H NMR (500 MHz, CDCl_3_) δ 7.41–7.44 (t, *J* = 7.5 Hz, 2H), 7.53–7.56 (t, *J* = 7.5 Hz, 1H), 7.73–7.75 (d, *J* = 8 Hz, 2H); ^13^C NMR (125 MHz, CDCl_3_) δ 113.0, 129.2, 130.9, 132.4, 133.8, 136.5, 143.9, 162.8; HRMS (DART): [M + H]^+^ calcd for C_20_H_10_N_6_, 335.1040; found, 335.1094; [M + NH_4_]^+^, 352.1305; found, 352.1358.

#### Dibenzo[*f*,*h*]pyrazino[2,3-*b*]quinoxaline-11,12-dicarbonitrile (**6a**)

An oven dried sealed tube was charged with 5,6-diaminopyrazine-2,3-dicarbonitrile (**12**, 0.73 g, 4.6 mmol), phenanthrene-9,10-dione (0.86 g, 4.1 mmol), glacial acetic acid (24 mL), TFA (6 mL) and 1,4-dioxane (12 mL). The resulting suspension was heated to 100 °C for 35 hours. By the end of the reaction, a red shimmering precipitate was observed. The reaction mixture was allowed to cool to room temperature, filtered and washed with copious amounts of dichloromethane and dried to obtain red-brick-colored solid **6a** (1.10 g, 3.30 mmol, 80%). ^1^H NMR (500 MHz, DMSO-*d*_6_) δ 7.88–7.91 (t, *J* = 7 Hz, 2H), 8.02–8.05 (t, *J* = 7.5 Hz, 2H), 8.83–8.84 (d, *J* = 8 Hz, 2H), 9.21–9.23 (d, *J* = 7 Hz, 2H). The compound was too insoluble for ^13^C NMR spectroscopic analysis.

### Synthesis of dihydropyrazinoquinoxaline diones **1b–7b** (DPQD) from dicyanopyrazinoquinoxalines (DCPQs) **1a**–**6a**

#### 1,4-Dihydropyrazino[2,3-*b*]quinoxaline-2,3-dione (**1b**)

To a 20 mL round-bottom flask was added pyrazino[2,3-*b*]quinoxaline-2,3-dicarbonitrile (**1a**, 146 mg, 0.628 mmol) and THF (10 mL). To the resulting orange suspension was dropwise added a solution of KOH (353 mg, 6.280 mmol) in water (10 mL) and reacted at room temperature for 3 hours. After this time the light orange solution was diluted with water (50 mL) and acidified with 1 N HCl until a white solid precipitated out. The solids were collected via filtration and dried in an oven (120 °C) for 24 hours. An off-white to pale yellow solid of **1b** was obtained (123 mg, 0.572 mmol, 91%). ^1^H NMR (600 MHz, DMSO-*d*_6_) δ 7.65–7.67 (dd, *J* = 3.6 Hz, *J* = 6.6 Hz, 2H), 7.84–7.86 (dd, *J* = 3.6 Hz, *J* = 6.6 Hz, 2H), 12.71 (s, 1H); ^13^C NMR (150 MHz, DMSO-*d*_6_) δ 126.7, 128.0, 137.4, 137.7, 155.6; HRESIMS: [M + Na]^+^ calcd for C_10_H_6_N_4_O_2_, 237.0388; found, 237.0394.

#### 7,8-Dimethyl-1,4-dihydropyrazino[2,3-*b*]quinoxaline-2,3-dione (**2b**)

Compound **2b** was synthesized with a method similar to that of **1b** using **2a** (97.7 mg, 0.378 mmol), THF (8 mL), and KOH (64 mg, 1.13 mmol) in water (8 mL). After reaction, the red-orange solution was diluted with water (50 mL) and acidified with 1 N HCl until a pink-colored solid precipitated out. The solids were collected via filtration using a fine sintered funnel and dried in an oven to afford off-white solid of **2b** (90 mg, 0.37 mmol, 99%). ^1^H NMR (500 MHz, DMSO-*d*_6_) δ 2.41 (s, 6H), 7.61 (s, 2H), 12.61 (s, 2H); ^13^C NMR (150 MHz, DMSO-*d*_6_) δ 19.7, 126.0, 136.1, 136.8, 137.9, 155.6; HRESIMS: [M + Na]^+^ calcd for C_12_H_10_N_4_O_2_, 265.0701; found, 265.0692.

#### 10,13-Dihydroaceanthryleno[1,2-*b*]pyrazino[2,3-*e*]pyrazine-11,12-dione (**3b**)

Compound **3b** was synthesized in the same way as **1b** using compound **3a** (10 mg, 0.03 mmol), THF (4 mL), 1,4-dioxane (1 mL), and KOH (10 equiv) in water (5 mL). The product **3b** was obtained as an orange precipitate after the reaction mixture was poured into 30 mL of ice and neutralized with 1 N HCl. Yield: 6 mg, 0.02 mmol, 67%. ^1^H NMR (500 MHz, DMSO-*d*_6_) δ 7.56–7.60 (t, *J* = 7.5 Hz, 1H), 7.71–7.74 (t, *J* = 7.5 Hz, 2H), 8.04–8.05 (d, *J* = 7 Hz, 1H), 8.21–8.23 (d, *J* = 8.5 Hz, 2H), 8.76 (s, 1H), 9.00–9.02 (d, *J* = 8.5 Hz, 1H), 12.72 (brs, 2H); ^13^C NMR (125 MHz, DMSO-*d*_6_ + TFA) δ 123.0, 124.3, 124.9, 126.0, 126.8, 127.9, 128.2, 128.8, 128.9, 129.8, 130.1, 130.4, 130.9, 133.5, 133.8, 134.4, 144.7, 147.0, 155.5, 155.6; HRESIMS: [M + K]^+^ calcd for C_20_H_10_N_4_O_2_, 377.0441; found, 377.0423.

#### 8,11-Dihydroacenaphtho[1,2-*b*]pyrazino[2,3-*e*]pyrazine-9,10-dione (**4b**)

Compound **4b** was synthesized using a method similar to that of **1b** using compound **4a** (102 mg, 0.333 mmol), THF (5 mL), and KOH (187 mg, 3.33 mmol) in water (5 mL). After the addition of KOH, the reaction mixture turned red-brown. After completion, the reaction mixture was diluted with water (10 mL) and acidified with concentrated HCl until a precipitate became apparent. The yellow-orange precipitate was filtered and washed with copious amounts of water and dried in vacuo (95 mg, 0.300 mmol, 99%). ^1^H NMR (500 MHz, DMSO-*d*_6_) δ 7.68–7.71 (t, *J* = 7.5 Hz, 2H), 7.96–7.98 (d, *J* = 7 Hz, 2H), 7.99–8.01 (d, *J* = 8.5 Hz, 2H), 11.57 (brs, 1H); ^1^H NMR (500 MHz, C_6_D_6_) δ 7.36–7.39 (t, *J* = 7 Hz, 2H), 7.64–7.65 (d, *J* = 8.3 Hz, 2H), 7.99–8.00 (d, *J* = 6.9 Hz, 2H), 12.94 (brs, 1H); ^13^C NMR (125 MHz, DMSO-*d*_6_) δ 121.9, 128.8, 129.1, 129.2, 130.7 131.3, 134.8, 145.3, 155.5; HRESIMS: [M + K]^+^ calcd for C_16_H_8_N_4_O_2_, 327.0284; found, 327.0279.

#### 6,7-Diphenyl-1,4-dihydropyrazino[2,3-*b*]pyrazine-2,3-dione (**5b**)

Compound **5b** was synthesized using a method similar to that of **1b** using compound **5a** (236 mg, 0.706 mmol), THF (9 mL), and KOH (400 mg, 7.06 mmol) in water (9 mL). The red solution was diluted with water (10 mL) and acidified with 1 N HCl until an orange precipitate of **5b** was obtained (198 mg, 0.628 mmol, 89%). ^1^H NMR (500 MHz, DMSO-*d*_6_) δ 7.32 (s, 10H), 12.70 (brs, 2H); ^13^C NMR (125 MHz, DMSO-*d*_6_) δ 128.5, 128.6, 129.9, 135.2, 138.4, 144.1, 156.1; HRESIMS: [M + H]^+^ calcd for C_18_H_12_N_4_O_2_, 317.1038; found, 317.1021.

#### 10,13-Dihydrodibenzo[*f*,*h*]pyrazino[2,3-*b*]quinoxaline-11,12-dione (**6b**)

Compound **6b** was synthesized similarly to **1b** using compound **6a** (1.11 g, 3.34 mmol, THF (50 mL), and KOH (1.87 g, 33.3 mmol) in water (50 mL). After completion, a yellow precipitate was observed which was filtered and washed with copious amounts of 1 N HCl and diethyl ether. The product **6b** was then dried in vacuo to obtain pale yellow amorphous solid (1.15 g, 3.31 mmol, 99%). ^1^H NMR (500 MHz, DMSO-*d*_6_) δ 7.60–7.63 (t, *J* = 7.5 Hz, 2H), 7.66–7.69 (t, *J* = 8.5 Hz, 2H), 8.71–8.73 (d, *J* = 8.5 Hz, 2H), 8.98–9.00 (d, *J* = 8 Hz, 2H); ^13^C NMR (125 MHz, DMSO-*d*_6_) δ 123.0, 123.8, 127.1, 129.1, 130.0, 132.6, 143.1, 160.4; ^13^C NMR (150 MHz, DMF-*d*_7_) δ 123.3, 124.0, 127.7, 128.6, 128.8, 129.9, 133.6, 136.5, 155.7; HRESIMS: [M + H]^+^ calcd for C_18_H_10_N_4_O_2_, 315.0881; found, 315.0877.

#### 10,13-Dihydropyrazino[2',3':5,6]pyrazino[2,3-*f*][1,10]phenanthroline-11,12-dione (**7b**)

In a 100 mL three-neck round-bottom flask, compounds **12** (100 mg, 0.624 mmol) and **7e** (150 mg, 0.713 mmol) were added to 15 mL of tetrahydrofuran (THF). Subsequently, a solution of potassium hydroxide in water (15 mL) was added dropwise at room temperature, resulting in a color change from orange to red. The reaction mixture stirred for 72 hours, resulted in the formation of an orange-brown precipitate. The reaction mixture was then filtered, and 1 N HCl was added until orange-red colored solids appeared. The solids were collected via filtration and washed with copious amounts of water and dried in an oven to obtain red-orange product **7b** (120 mg, 0.381 mmol, 61%). ^1^H NMR (500 MHz, DMSO-*d*_6_) δ 7.78–7.81 (dd, *J* = 4.5 Hz, 8.5 Hz, 2H), 8.96–8.98 (d, *J* = 7.5 Hz, 2H), 9.34–9.36 (d, *J* = 8 Hz, 2H); ^13^C NMR (125 MHz, DMSO-*d*_6_ + TFA) δ 126.8, 127.8, 136.8, 138.3, 139.0, 147.9, 155.8; HRESIMS: [M + H]^+^ calcd for C_16_H_8_N_6_O_2_, 317.0787; found, 317.0776.

## Supporting Information

File 1 General experimental methods, ^1^H and ^13^C NMR, and HRMS spectra of the compounds as well as photophysical, computational and X-ray data.

## Data Availability

All data that supports the findings of this study is available in the published article and/or the supporting information to this article.
