# Supplementary material for: Structure–property relationships in dicyanopyrazinoquinoxalines and their hydrogen-bonding-capable dihydropyrazinoquinoxalinedione derivatives
Source: Beilstein J Org Chem. 2024 May 8;20:1037–52. doi: 10.3762/bjoc.20.92 (PMC11092063; doi:10.3762/bjoc.20.92)
Supplement: File 1 — General experimental methods, 1H and 13C NMR, and HRMS spectra of the compounds as well as photophysical, computational and X-ray data. [file Beilstein_J_Org_Chem-20-1037-s001.pdf]

BEILSTEIN JOURNAL OF ORGANIC CHEMISTRY

Supporting Information

for

Structure–property relationships in dicyanopyrazinoquinoxalines and their hydrogen-bonding-capable dihydropyrazinoquinoxalinedione derivatives

Tural N. Akhmedov, Ajeet Kumar, Daken J. Starkenburg, Kyle J. Chesney, Khalil A. Abboud, Novruz G. Akhmedov, Jiangeng Xue and Ronald K. Castellano

Beilstein J. Org. Chem. **2024,** *20,* 1037–1052. doi:10.3762/bjoc.20.92

General experimental methods, ^{1}H and ^{13}C NMR, and HRMS spectra of the compounds as well as photophysical, computational and X-ray data

License and Terms: This is a supporting information file under the terms of the Creative Commons Attribution License (https://creativecommons.org/licenses/by/4.0). Please note that the reuse, redistribution and reproduction in particular requires that the author(s) and source are credited and that individual graphics may be subject to special legal provisions.
The license is subject to the *Beilstein Journal of Organic Chemistry* terms and conditions: (https://www.beilstein-journals.org/bjoc/terms)

Table of Contents

Synthetic details S2
Synthesis of compounds S3
Thermogravimetric analysis (TGA) S11
Electrochemistry S12
Mass spectrometry S13
NMR spectroscopy S19
UV–visible spectroscopy S47
X-ray diffraction S52
References S59

SYNTHETIC DETAILS

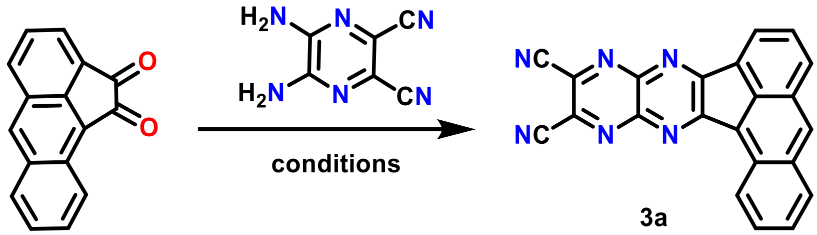

Scheme S1. Synthesis of **3a**

Table S1. Different reaction conditions employed to access **3a**

Entry	Conditions	Result
1	AcOH, 120 °C, 24-72h	incomplete
2	TFA, AcOH, 1,4-dioxane, 120 °C, 24-72h	mixture
3	Ethanol, AcOH, reflux, 48h	NR
4	Ethanol, reflux, 24-72h	NR
5	Toluene, 120 °C, 24h	NR
6	Pyridine, 115 °C, 24-72h	promising
7	Pyridine, 80 °C, 36h	41%

N.R.= No Result

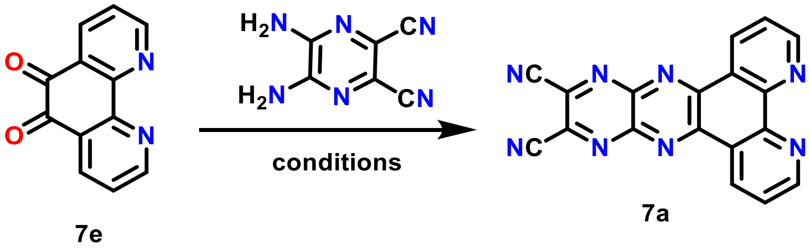

Scheme S2. Synthesis of **7a**

Table S2. Different conditions employed for the synthesis of **7a**

**Entry**	**Conditions**	**Result**
1	AcOH, 120 °C, 12-72h	mixture
2	TFA, AcOH, 1,4-dioxane, 120 °C, 12-72h	mixture
3	Methanol or Ethanol, AcOH, reflux, 48h	N.R.
4	Methanol or Ethanol, reflux, 12-72h	N.R.
5	Toluene, 120 °C, 12-24h	N.R.
6	Pyridine, 115 °C, 24-72h	decomp.
7	Pyridine, 80 °C, 48h	decomp.
8	Ethylene glycol, 135 °C, 48h	**16a** (18%)
9	DMF, 100 °C, 12h	mixture
10	NEt_3 (0.2 or 2 eq), DMF or MeCN, reflux, 12h	mixture
11	K_2CO_3 (1 eq), EtOH, reflux, 12h	mixture
12	NEt_3 (30 eq), EtOH, rt, 72h	**16b** (45%)
13	KOH (10 eq), THF, H_2O, rt, 12h	**7b** (61%)

N.R.= No Result

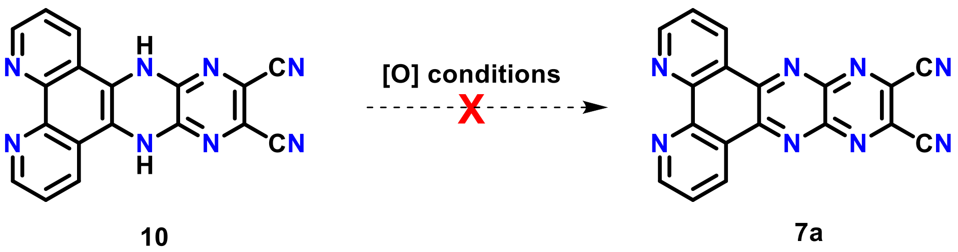

Scheme S3. Oxidation of **10**

Table S3. Different conditions employed for the oxidation of **10** to **7a**

Entry	Conditions	Result
1	PbO_2, CH_2Cl_2, rt	mixture
2	MnO_2, CH_2Cl_2, rt	mixture
3	DDQ, CH_2Cl_2, rt	mixture
4	DDQ, THF, rt	mixture
5	DDQ, DMSO, rt	decomp.
6	DDQ, DMF, rt	decomp.

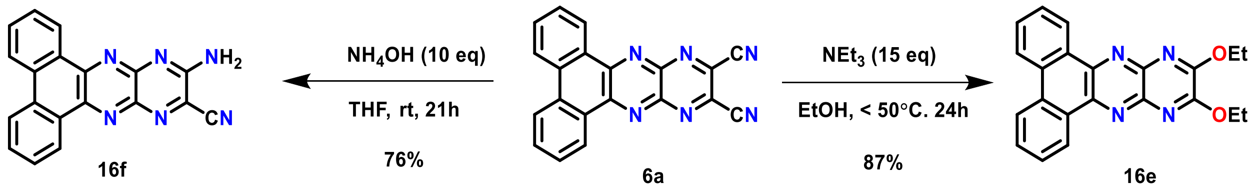

Scheme S4. Synthesis of substituted **6a** derivatives, **16e** and **16f**.

SYNTHESIS OF COMPOUNDS

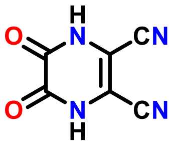

14

5,6-Dioxo-1,4,5,6-tetrahydropyrazine-2,3-dicarbonitrile (14)

A 2 L Erlenmeyer flask was charged with a 2.0 M solution of oxalyl chloride (140 mL, 278 mmol). To the solution stirring at a high rate, was slowly dropwise added a solution of diaminomaleonitrile (DAMN) **15** (15.0 g, 139 mmol) in anhydrous 1,4-dioxane (300 mL) over the course of one hour. The resulting pale-yellow suspension was gently warmed to 45 °C overnight.

After this time the mixture, which turned yellow-brown was cooled, filtered, and washed with copious amount of hexanes. The crude material was recrystallized from methanol to obtain off-white crystals of **14** (11 g, 68 mmol, 49%). ^{13}C NMR (151 MHz, DMSO-d_6): δ 106.17, 111.34, 155.21; HRMS (DART) calc'd for $C_6H_2N_4O_2$ $[M+H]^+$: 163.0256, found: 163.0252; $[M+NH_4]^+$: 180.0521, found: 180.0518. The ^{13}C NMR spectrum matches the values reported in the literature.[1]

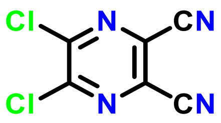

13

5,6-Dichloropyrazine-2,3-dicarbonitrile (13)

A 500 mL round-bottom flask was charged with 5,6-dioxo-1,4,5,6-tetrahydropyrazine-2,3-dicarbonitrile (**14**, 11.0 g, 67.9 mmol) and suspended in 1,4-dioxane (80 mL). Thionyl chloride (75.0 mL, 1030 mmol) was added at a rate of 1 drop/s over the course of an hour using an addition funnel. Additional 1,4-dioxane (10 mL) was used to rinse the addition funnel. After the addition, DMF (0.8 mL, 10 mmol) was added dropwise to the yellow suspension. The reaction mixture was heated to reflux at 100 °C for 5 hours at which point the suspension turned from yellow to brown. After this time the flask was cooled and thionyl chloride was removed in vacuo to obtain a brown solid. Column chromatography in CH_2Cl_2 afforded an off-yellow crystalline product (10.0 g, 50.2 mmol, 74.0%). ^{13}C NMR (125 MHz, $CDCl_3$): δ 111.6, 130.3, 152.3. ^{1}H NMR spectra matches with the literature.[2]

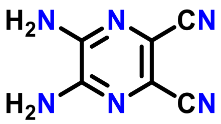

12

5,6-Diaminopyrazine-2,3-dicarbonitrile (12)

A 500 mL round-bottom flask was charged with 5,6-dichloropyrazine-2,3-dicarbonitrile (**13**, 10.14 g, 51 mmol) and dissolved in dry THF (220 mL) and placed in a water bath at room temperature. To the resulting red-brown solution was dropwise added ammonia (29% w/w) (34 mL, 510 mmol) using an addition funnel. The reaction mixture was heated to reflux for 20 hours. After this time, the flask was cooled, and the contents transferred to 500 mL of ice and mixed well. After extraction with EtOAc (4 × 100 mL) the combined organic layers were washed with brine (100 mL) and dried with Na_2SO_4. The solvents were removed in vacuo to obtain pure orange

amorphous solid (7.4 g, 46 mmol, 91%). ^{1}H NMR (500 MHz, DMSO-d_6): δ 7.55 (br s, 4H); ^{13}C NMR (125 MHz, DMSO-d_6): δ 115.8, 118.8, 145.5.

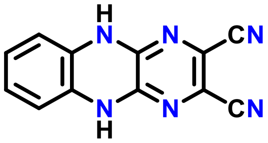

8

5,10-Dihydropyrazino[2,3-*b*]quinoxaline-2,3-dicarbonitrile (8)

To a solution of 5,6-dichloropyrazine-2,3-dicarbonitrile (**13**, 50 mg, 0.25 mmol) in 1,4-dioxane (3 mL) was added 1,2-phenylenediamine (60 mg, 0.55 mmol) in small portions. Within a few minutes, an orange precipitate formed. The reaction proceeded at room temperature for 12 hours after which a pale orange solid was observed. The solid was filtered and washed with water and diethyl ether and dried in vacuo to leave a bright orange amorphous material (45 mg, 0.46 mmol, 84%). ^{1}H NMR (500 MHz, DMSO-d_6): δ 6.36–6.38 (dd, 2H, J = 3.5 Hz, J = 6 Hz), 6.59–6.61 (dd, 2H, J = 3.5 Hz, J = 6 Hz), 10.23 (s, 1H); HRMS (ESI) calculated for $C_{12}H_6N_6$ $[M+H]^+$: 235.0732, found: 235.0732.

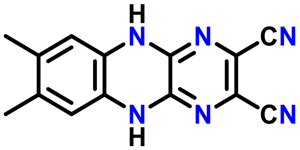

9

7,8-Dimethyl-5,10-dihydropyrazino[2,3-*b*]quinoxaline-2,3-dicarbonitrile (9)

To a purple solution of 5,6-dichloropyrazine-2,3-dicarbonitrile (**13**, 300 mg, 1.50 mmol), in 1,4-dioxane (18 mL) was added 1,2-phenylene-4,5-diamine (359 mg, 3.31 mmol) in small portions; within a few minutes, an orange precipitate formed. The reaction was allowed to proceed at room temperature for 12 hours. The solid was filtered and washed with water (30 mL) and diethyl ether (30 mL), then dried in vacuo to leave a bright orange amorphous material (300 mg, 2.81 mmol, 85%). ^{1}H NMR (500 MHz, DMSO-d_6): δ 1.92 (s, 6H), 6.18 (s, 2H), 10.15 (s, 2H). Proton NMR spectra matches closely with the literature report.[3]

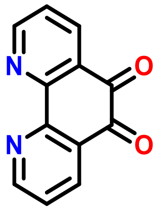

7e

1,10-Phenanthroline-5,6-dione (7e)

A cooled 500 mL round-bottom flask was charged with phenanthroline (7.0 g, 39 mmol), potassium bromide (11.6 g, 58.3 mmol) to which then was added chilled (ca. 0 °C) conc. sulfuric acid (70 mL) very slowly. After this time, conc. nitric acid (30 mL) was introduced to the dark orange suspension and heated to 100 °C for 18 hours. After this time, the yellow solution was cooled, added to ice (1 kg) and then slowly neutralized to pH ≈ 7 with a solution of NaOH (≈100 g) in water. After neutralization, the aqueous solution was extracted with dichloromethane (6 × 100 mL), the combined organic layer washed with brine (200 mL) and dried with anhydrous Na_2SO_4. After recrystallization of the crude residue with methanol, was left with yellow crystalline powder of **7e** (5.1 g, 63%). ^{1}H NMR (500 MHz, $CDCl_3$): δ 7.58–7.60 (dd, 2H, J = 5 Hz J = 8.0 Hz), 8.50–8.52 (d, 2H, J = 7.8 Hz), 9.12–9.13 (d, 2H, J = 5.0 Hz). Proton NMR matches the literature.[4]

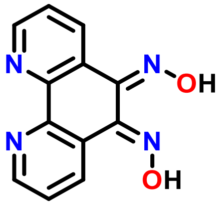

(5*Z*,6*E*)-1,10-Phenanthroline-5,6-dione dioxime (7d)

A 500 mL round-bottom flask was charged with 1,10-phenanthroline-5,6-dione (**7e**, 2.17 g, 10.3 mmol), hydroxyl amine hydrochloride (2.15 g, 31 mmol), barium carbonate (4.07 g, 20.64 mmol), and ethanol (220 mL). The resulting yellow suspension was heated to reflux for 24 hours. After this time, the pale-yellow solid was collected by filtration and re-added to a new flask and stirred in a solution of 0.2 N HCl overnight. After filtration, a pure, pale-yellow solid was obtained which was washed with water and dried in vacuo (1.50 g, 60%). ^{1}H NMR (500 MHz, DMSO-d_6): δ 7.51–7.57 (m, 2H), 8.25–8.29 (d, 1H, J = 13 Hz), 8.79–8.81 (d, 2H, J = 7 Hz), 8.92–8.95 (d, 1H, J = 13 Hz), 12.90 (s, 1H), 12.99 (brs, 1H). The proton NMR spectrum matches the literature.[5]

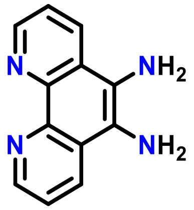

7c

1,10-Phenanthroline-5,6-diamine (7c)

A 100 mL 3-neck round-bottom flask was charged with (5*Z*, 6*E*)-1,10-phenanthroline-5,6-dione dioxime (**7d**, 113 mg, 0.53 mmol), Pd/C (113 mg) and ethanol (30 mL). The suspension was brought to a reflux for 26 hours as hydrazine monohydrate ($N_2H_4.H_2O$, 40 mL) was slowly added using an addition funnel. After this time, the catalyst was filtered off at room temperature on a

bed of celite. The red colored filtrate was concentrated in vacuo to obtain the crude solid. To the solid was added water (30 mL); the mixture was then sonicated and stirred at room temperature for 1 hour. After this time, the mixture was filtered and the solid residue was washed with water to obtain pure yellow-green colored solid of **7c** (58 mg, 0.24 mmol, 46%). ^{1}H NMR (500 MHz, DMSO-d_6): δ 5.21 (s, 4H), 7.59–7.62 (dd, 2H, J = 4 Hz, J = 8 Hz), 8.47–8.49 (d, 2H, J = 8 Hz), 8.77–8.78 (d, 2H, J = 2.5 Hz). The proton NMR spectrum matches the literature.[6]

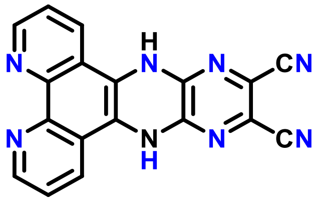

10

9,14-Dihydropyrazino[2',3':5,6]pyrazino[2,3-*f*][1,10]phenanthroline-11,12-dicarbonitrile (10)

A 5 mL round-bottom flask was charged with 1,10-phenanthroline-5,6-diamine (**7c**, 50 mg, 0.24 mmol) and 5,6-dichloropyrazine-2,3-dicarbonitrile (**13**, 47.3 mg, 0.24 mmol), and suspended in 1,4-dioxane (3 mL). The mixture was heated to 80 °C for 26 hours. After this time the reaction mixture was filtered and washed with dichloromethane (15 mL) to obtain a light brown solid (47 mg, 0.14 mmol, 59%). ^{1}H NMR (500 MHz, DMSO-d_6): δ 7.99–8.01 (dd, 2H, J = 5 Hz J = 8.5 Hz), 8.89–8.90 (d, 2H, J = 3.5 Hz), 8.93–8.95 (d, 2H, J = 8.5 Hz), 12.72 (brs, 2H); HRMS (negative ESI) calculated for $C_{18}H_8N_8$ $[M]^-$: 336.0872, found: 336.0671; $[M-2H]^-$: 334.0716, found: 334.0737.

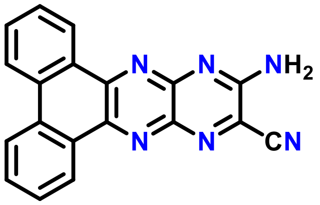

16f

12-Aminodibenzo[*f*,*h*]pyrazino[2,3-*b*]quinoxaline-11-carbonitrile (16f)

A 50 mL round-bottom flask was charged with dibenzo[*f*,*h*]pyrazino[2,3-*b*]quinoxaline-11,12-dicarbonitrile (**6a**, 64 mg, 0.20 mmol) and suspended in THF (15 mL). To the resulting red suspension was dropwise added ammonium hydroxide (15 M; 0.13 mL, 2.0 mmol) in THF (5 mL) and allowed to react overnight for 21 hours. After this time, the reaction mixture was diluted with water (15 mL) and neutralized with 1 N HCl to pH of 7. The resulting orange solid was filtered and dried in vacuo (45 mg, 0.15 mmol, 76%). ^{1}H NMR (600 MHz, DMSO-d_6): δ 7.80–7.84 (m, 2H), 7.87–7.89 (t, 1H, J = 7.2 Hz), 7.93–7.95 (t, 1H, J = 7.2 Hz), 8.24 (br s, 2H), 8.79–8.82 (t, 1H, J = 7.5 Hz), 9.07–9.09 (d, 1H, J = 7.8 Hz), 9.13–9.15 (d, 1H, J = 7.8 Hz); ^{13}C NMR (150 MHz, DMSO-d_6): δ 114.60, 123.77, 123.83, 125.04, 125.35, 126.50, 128.41, 128.60, 128.73, 129.05, 130.68, 131.00,

131.96, 132.56, 138.93, 140.57, 145.98, 146.06, 155.22; HRMS (ESI) calculated for $C_{19}H_{10}N_6$ $[M+H]^+$: 323.1045, found: 323.1025.

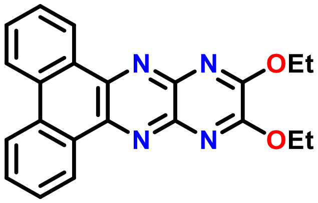

16e

11,12-Diethoxydibenzo[*f*,*h*]pyrazino[2,3-*b*]quinoxaline (16e)

A 50 mL round-bottom flask was charged with dibenzo[*f*,*h*]pyrazino[2,3-*b*]quinoxaline-11,12-dicarbonitrile (**6a**, 55 mg, 0.17 mmol) and the solid was suspended in ethanol (20 mL). To the resulting red suspension was added trimethylamine (0.7 mL, 5 mmol). The mixture was heated to 50 °C for 24 hours after which a bright yellow precipitate formed. The solid was collected via filtration and dried in vacuo (53 mg, 0.14 mmol, 87%). ^1H NMR (500 MHz, DMSO-d_6): δ 1.50–1.52 (t, 6H, J = 7 Hz), 4.66–4.71 (q, 4H, J = 6 Hz), 7.81–7.84 (t, 2H, J = 7.5 Hz), 7.87–7.90 (t, 2H, J = 8 Hz), 8.84–8.86 (d, 2H, J = 8.5 Hz), 9.16–9.18 (t, 2H, J = 7.5 Hz); HRMS (ESI) calculated for $C_{22}H_{18}N_4O_2$ $[M+H]^+$: 371.1508, found: 371.1495.

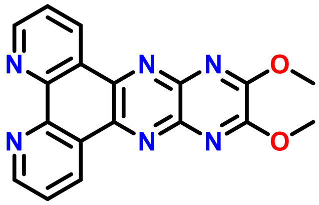

16c

11,12-Dimethoxypyrazino[2',3':5,6]pyrazino[2,3-*f*][1,10]phenanthroline (16c)

A 25 mL round-bottom flask was charged with 5,6-diaminopyrazine-2,3-dicarbonitrile (**12**, 55 mg, 0.34 mmol) and 1,10-phenanthroline-5,6-dione (**7e**, 72.5 mg, 0.312 mmol), and the mixture was dissolved in methanol (10 mL). To the resulting light orange solution was dropwise added a solution of trimethylamine (3.0 mL, 26 mmol), which turned the solution red. The reaction was gently heated to 50 °C for 16 hours at which point a yellow precipitate became apparent. The solids were collected via filtration and washed with copious amounts of methanol and dried in vacuo to obtain pale yellow material (56 mg, 0.20 mmol, 52%). ^1H NMR (500 MHz, DMSO-d_6): δ 4.26 (s, 6H) 7.96–7.98 (dd, 2H, J = 4.5 Hz, J = 8 Hz), 9.24–9.25 (d, 2H, J = 4.5 Hz), 9.51–9.52 (d, 2H, J = 8 Hz); ^{13}C NMR (125 MHz, $CDCl_3$): δ 56.17, 124.14, 126.95, 134.08, 140.24, 142.72, 148.07, 152.67, 154.37; HRMS (ESI) calculated for $C_{18}H_{12}N_6O_2$ $[M+Na]^+$: 367.0919, found: 367.0907.

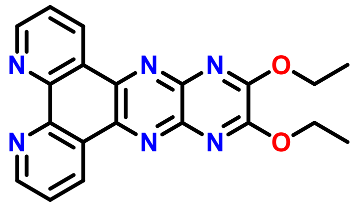

16b

11,12-Diethoxypyrazino[2',3':5,6]pyrazino[2,3-*f*][1,10]phenanthroline (16b)

A 50 mL round-bottom flask was charged with 5,6-diaminopyrazine-2,3-dicarbonitrile (**12**, 100 mg, 0.62 mmol) and 1,10-phenanthroline-5,6-dione (**7e**, 84 mg, 0.42 mmol), and the solids were dissolved in methanol (24 mL). To the resulting orange solution was added trimethylamine (6 mL, 100 mmol) and the solution was left to react at room temperature for 72 hours. After this time, the reaction mixture was filtered to obtain a pale yellow solid (70 mg, 0.19 mmol, 45%). ^{1}H NMR (500 MHz, DMSO-d_6): δ 1.51–1.53 (t, 6H, J = 7 Hz), 4.70–4.73 (q, 4H, J = 7 Hz), 7.94–7.97 (dd, 2H, J = 4 Hz, J = 7.5 Hz), 9.22–9.23 (d, 2H, J = 3.5 Hz), 9.48–9.50 (d, 2H, J = 8 Hz); ^{13}C NMR (125 MHz, $CDCl_3$): δ 14.63, 65.54, 124.33, 127.28, 134.30, 140.22, 143.01, 148.22, 152.76, 154.40; HRMS (DART) calculated for $C_{20}H_{16}N_6O_2$ [M+H]$^+$: 373.1413, found: 373.1424.

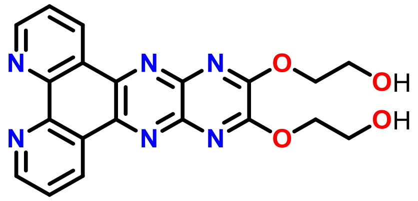

16a

2,2'-(Pyrazino[2',3':5,6]pyrazino[2,3-*f*][1,10]phenanthroline-11,12-diylbis(oxy))bis(ethan-1-ol) (16a)

A 25 mL round-bottom flask was charged with 5,6-diaminopyrazine-2,3-dicarbonitrile (**12**, 55 mg, 0.34 mmol), 1,10-phenanthroline-5,6-dione (**7e**, 73 mg, 0.31 mmol), and ethylene glycol (10 mL). The suspension was warmed to 50 °C before addition of triethylamine (0.65 mL, 4.7 mmol), and heated at this temperature for 9 hours. After this time the reaction mixture was allowed to cool and filtered to obtain a solid material which was washed with methanol to obtain an off-white solid **16a** (74 mg, 59%).

^{1}H NMR (500 MHz, DMSO-d_6): δ 3.92–3.95 (q, 4H, J = 5 Hz), 4.70–4.72 (br s, 4H), 5.06–5.08 (t, 2H, J = 5.5 Hz), 7.93–7.96 (dd, 2H), 9.22–9.23 (br s, 2H), 9.41–9.46 (d, 2H); ^{13}C NMR (125 MHz, DMSO-d_6): δ 58.63, 69.90, 125.29, 126.40, 132.86, 138.58, 142.33, 146.94, 151.70, 153.72; HRMS (ESI) calculated for $C_{20}H_{16}N_6O_4$ [M+H]$^+$: 405.1311, found: 405.1323.

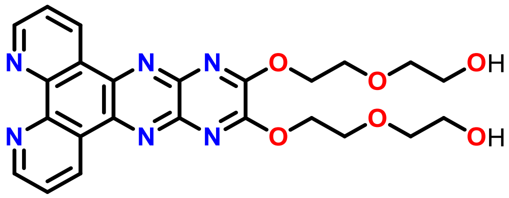

16d

2,2'-(((Pyrazino[2',3':5,6]pyrazino[2,3-*f*][1,10]phenanthroline-11,12-diylbis(oxy))bis(ethane-2,1-diyl))bis(oxy))bis(ethan-1-ol) (16d)

A 25 mL round-bottom flask was charged with 5,6-diaminopyrazine-2,3-dicarbonitrile (**12**, 55 mg, 0.34 mmol) and 1,10-phenanthroline-5,6-dione (**7e**, 72.5 mg, 0.312 mmol), and the solids were suspended in diethylene glycol (10 mL). The suspension was warmed to 50 °C before adding triethylamine (0.65 mL, 4.70 mmol) and heating was continued at this temperature for 16 hours. After this time the reaction mixture was cooled and filtered to obtain a solid material which was washed with copious amounts of methanol. An off-white solid was obtained (67 mg, 0.13 mmol, 43%). ^{1}H NMR (300 MHz, $CDCl_3$): δ 1.81 (br s, 2H), 3.74–3.76 (dd, 4H, *J* = 5.0 Hz, *J* = 3.1 Hz), 3.80–3.83 (dt, 4H, *J* = 5.3 Hz, *J* = 2.9 Hz), 4.04–4.07 (m, 4H), 4.93–4.96 (m, 4H), 7.78–7.82 (dd, 2H, *J* = 8.2 Hz, *J* = 4.4 Hz), 9.29–9.30 (d, 2H, *J* = 4.4. Hz), 9.61–9.65 (d, 2H, *J* = 8.2 Hz); ^{13}C NMR (75 MHz, $CDCl_3$): δ 61.78, 68.39, 68.63, 72.95, 124.16, 126.95, 134.08, 140.39, 142.55, 148.13, 152.72, 153.73.

THERMOGRAVIMETRIC ANALYSIS (TGA)
TGA was conducted on a TA Instruments TGA Q5000 V3.8 Build 256 using 1 mg of sample in a 100 μL platinum pan.

Table S4. Thermogravimetric analysis of **1a**-**6a** and **1b**-**7b**

compound	5% mass loss (°C)
AnthCN (**3a**)	**353**
AnthHB (**3b**)	**332**
BenzCN (**1a**)	**232**
BenzHB (**1b**)	**331**
BenzilCN (**5a**)	**238**
BenzilHB (**5b**)	**288**
NaphCN (**4a**)	**304**
NaphHB (**4b**)	**324**
PhenCN (**6a**)	**312**
PhenHB (**6b**)	**305**
PhenNNHB (**7b**)	**361**
XylCN (**2a**)	**244**
XylHB (**2b**)	**297**

ELECTROCHEMISTRY

Cyclic Voltammetry (CV) measurements were performed on **DCPQs 4a** and **6a** and **DPQDs 4b** and **6b**, using a single-compartment three-electrode cell with a gold counter electrode, an Ag/Ag^{+} reference electrode, and a platinum disk (0.02 cm^2) as the working electrode (Figure S1). Electrodes were purchased from CH Instruments. Tetrabutylammonium hexafluorophosphate ($TBAPF_6$) was purchased from Aldrich and kept dry under vacuum. DMF was collected from an Innovative Technologies solvent system, sparged with Ar and passed over two columns of 5 Å activated molecular sieves. The compounds were dissolved to a concentration of 1 mM in a 0.1 M $TBAPF_6$/DMF electrolyte. Potential sweeps were controlled by a Princeton Applied Research VersaSTATII potentiostat. The scan rate for CV was 100 mV/s. The boxed equation in Figure S1 was used to estimate LUMO levels from the CV data.[7] A reported CV of **6a** in benzonitrile exhibits two reversible reduction peaks[8] instead of one observed in DMF (Figure S1, in red).

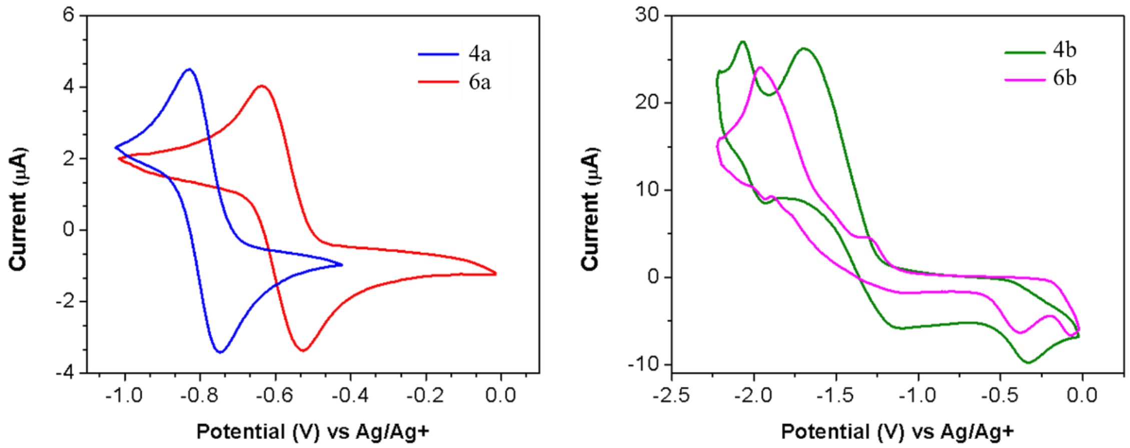

$$E_{LUMO} = -(E_{red}^{onset} + 5.1\ eV)$$

4a

$E_{red}^{onset} = -0.716$ V

$E_{LUMO} = -4.38$ eV

6a

$E_{red}^{onset} = -0.506$ V

$E_{LUMO} = -4.60$ eV

4b

$E_{red}^{onset} = -1.25$V

$E_{LUMO} = -\ 3.85$ eV

6b

$E_{red}^{onset} = -1.15$ V

$E_{LUMO} = -3.95$ eV

Figure S1. CV of **DCPQs 4a** and **6a** (on left) and **DPQDs 4b** and **6b** (on right).

MASS SPECTROMETRY

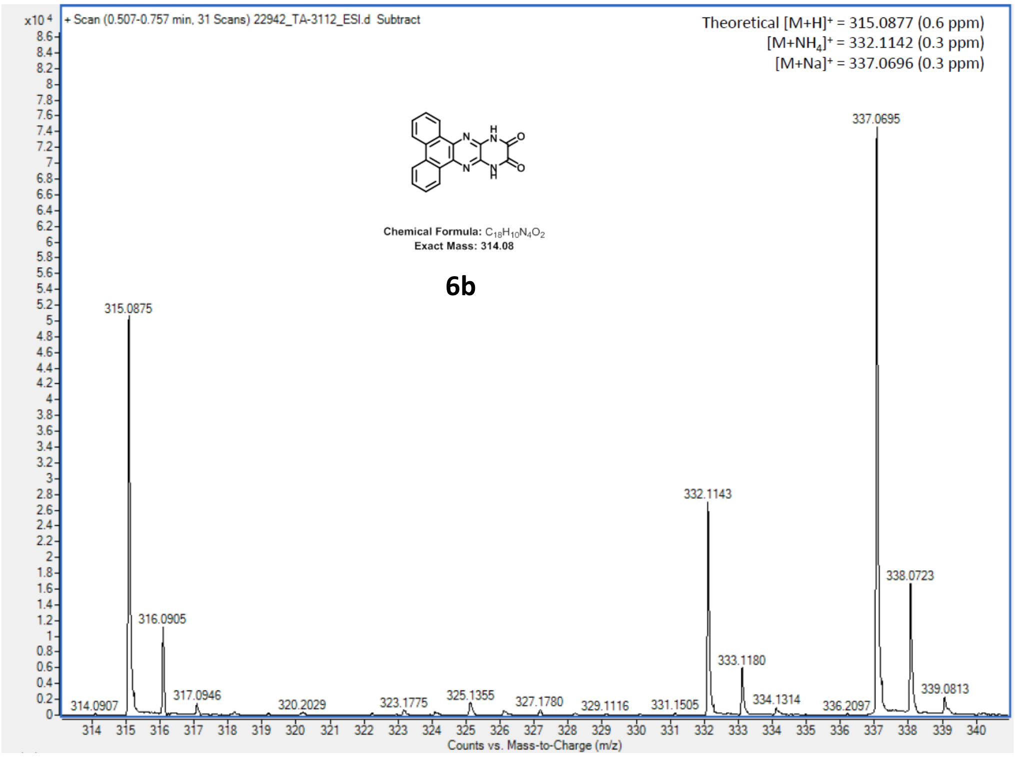

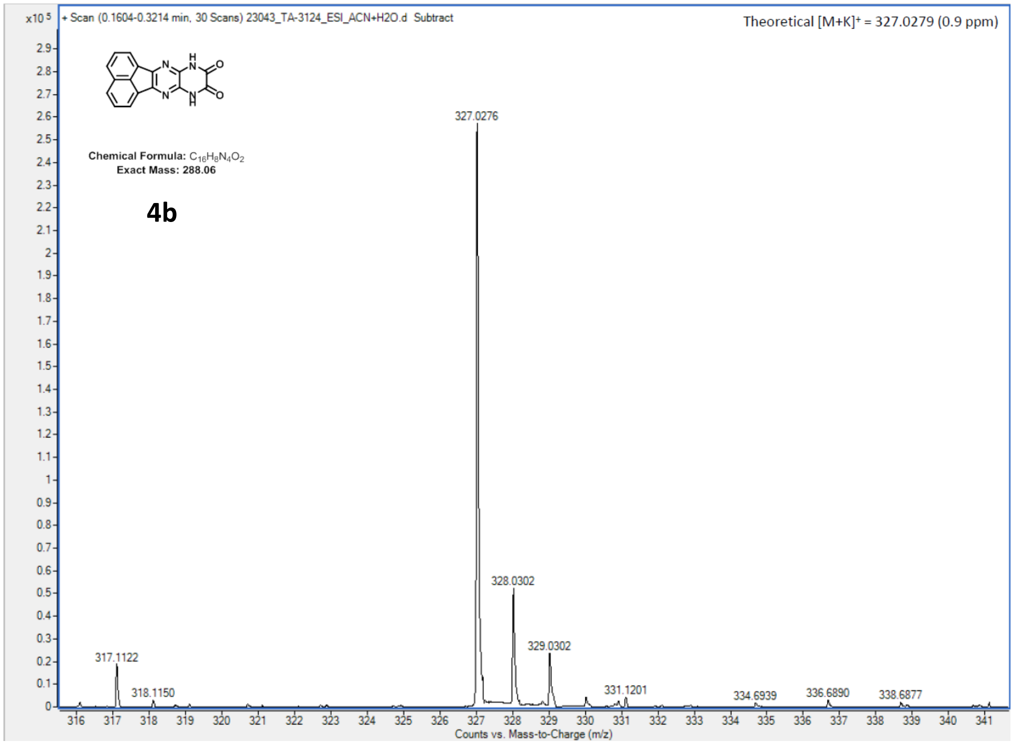

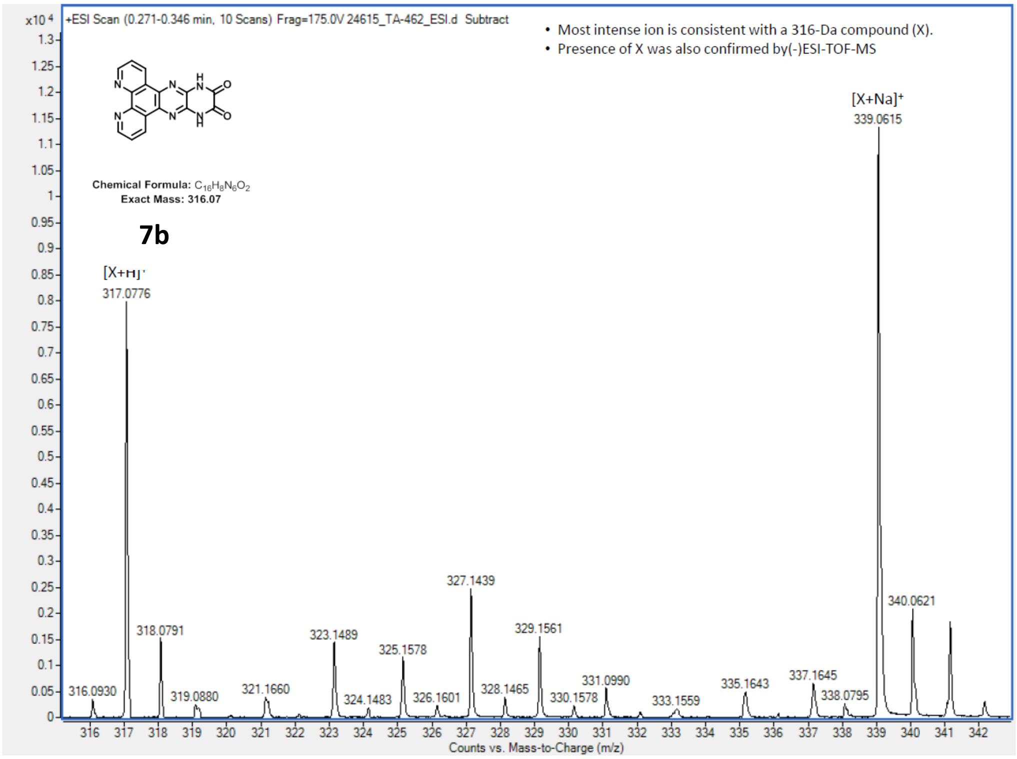
+ESI Scan (0.271-0.346 min, 10 Scans) Frag=175.0V 24615_TA-462_ESI.d Subtract
• Most intense ion is consistent with a 316-Da compound (X).
• Presence of X was also confirmed by(-)ESI-TOF-MS
Chemical Formula: $C_{16}H_8N_6O_2$
Exact Mass: 316.07
7b
[X+Na]+
339.0615
317.0776
340.0621
327.1439
329.1561
318.0791
323.1489
325.1578
Counts vs. Mass-to-Charge (m/z)

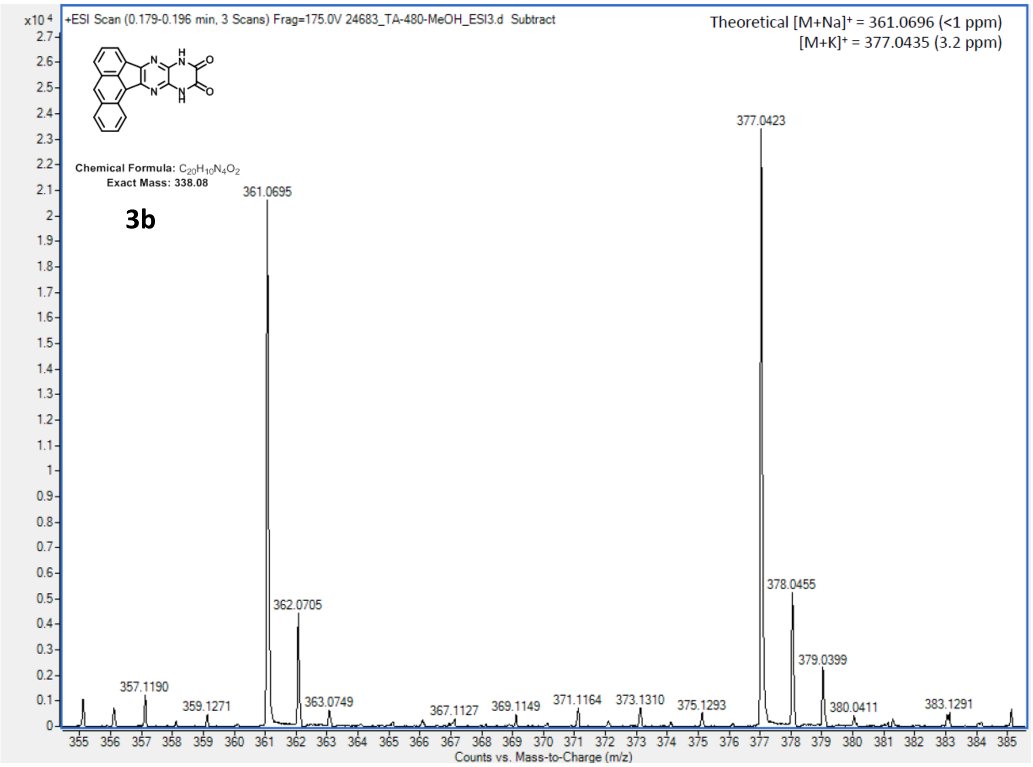
+ESI Scan (0.179-0.196 min, 3 Scans) Frag=175.0V 24683_TA-480-MeOH_ESI3.d Subtract
Theoretical [M+Na]+ = 361.0696 (<1 ppm)
[M+K]+ = 377.0435 (3.2 ppm)
Chemical Formula: $C_{20}H_{10}N_4O_2$
Exact Mass: 338.08
3b
377.0423
361.0695
378.0455
362.0705
379.0399
Counts vs. Mass-to-Charge (m/z)

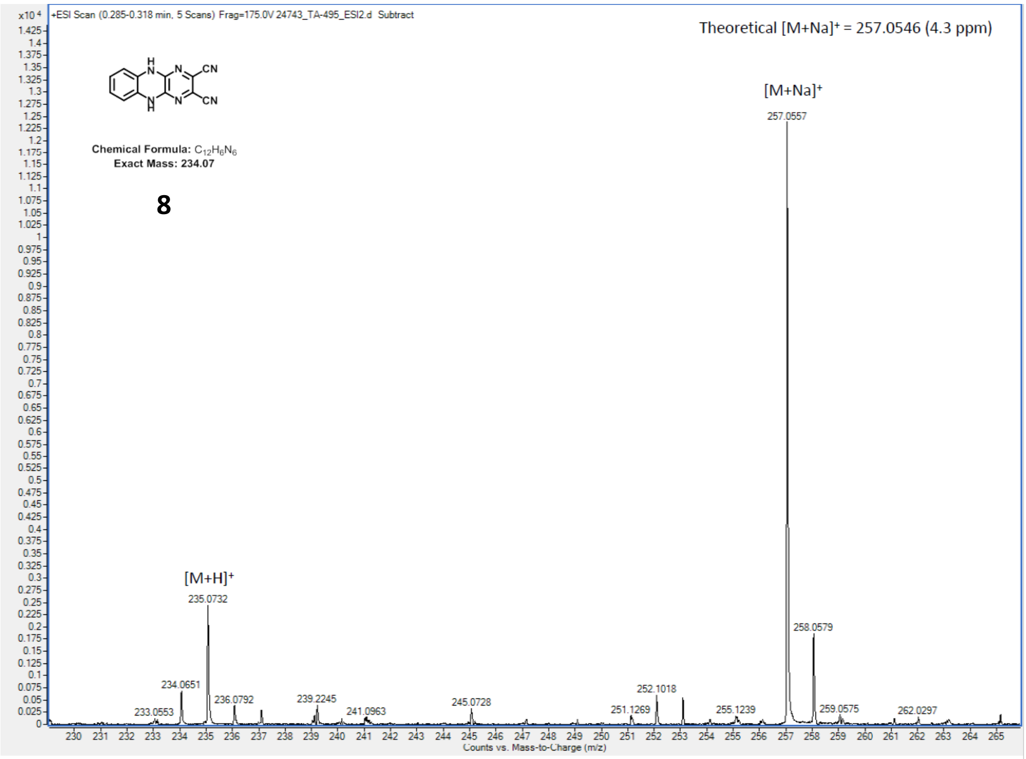
+ESI Scan (0.285-0.318 min, 5 Scans) Frag=175.0V 24743_TA-495_ESI2.d Subtract
Theoretical [M+Na]+ = 257.0546 (4.3 ppm)
Chemical Formula: C12H6N6
Exact Mass: 234.07
8
[M+Na]+
257.0557
[M+H]+
235.0732
258.0579
234.0651
233.0553
236.0792
239.2245
241.0963
245.0728
251.1269
252.1018
255.1239
259.0575
262.0297
Counts vs. Mass-to-Charge (m/z)

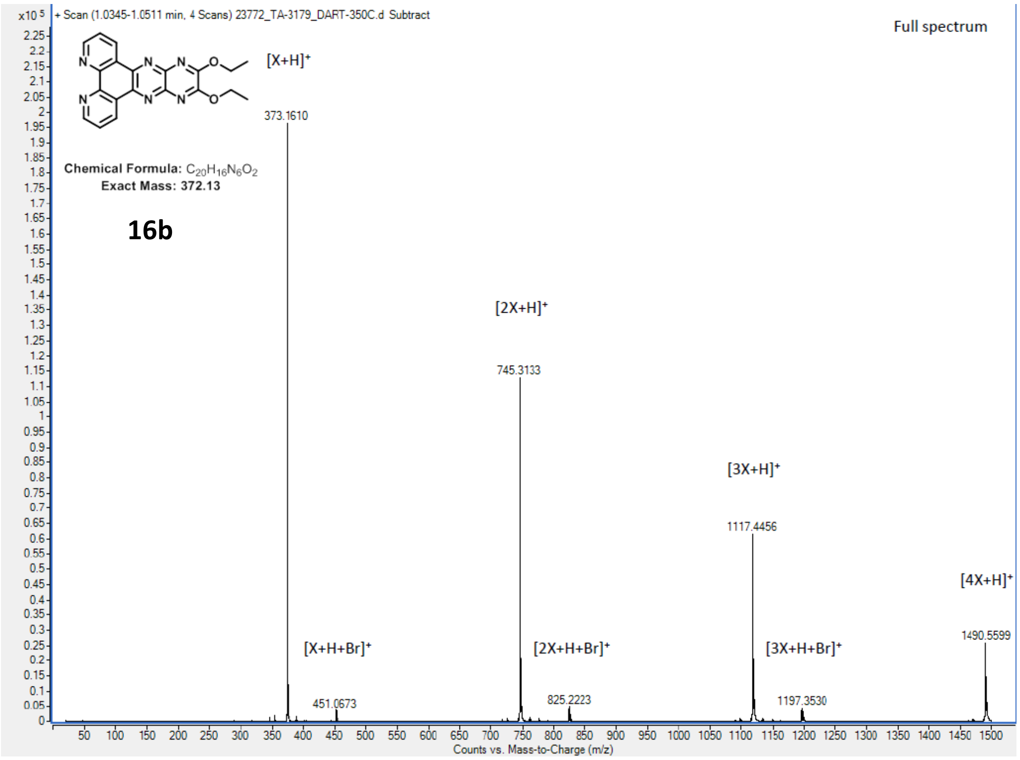
+ Scan (1.0345-1.0511 min, 4 Scans) 23772_TA-3179_DART-350C.d Subtract
Full spectrum
[X+H]+
373.1610
Chemical Formula: C20H16N6O2
Exact Mass: 372.13
16b
[2X+H]+
745.3133
[3X+H]+
1117.4456
[4X+H]+
1490.5599
[X+H+Br]+
451.0673
[2X+H+Br]+
825.2223
[3X+H+Br]+
1197.3530
Counts vs. Mass-to-Charge (m/z)

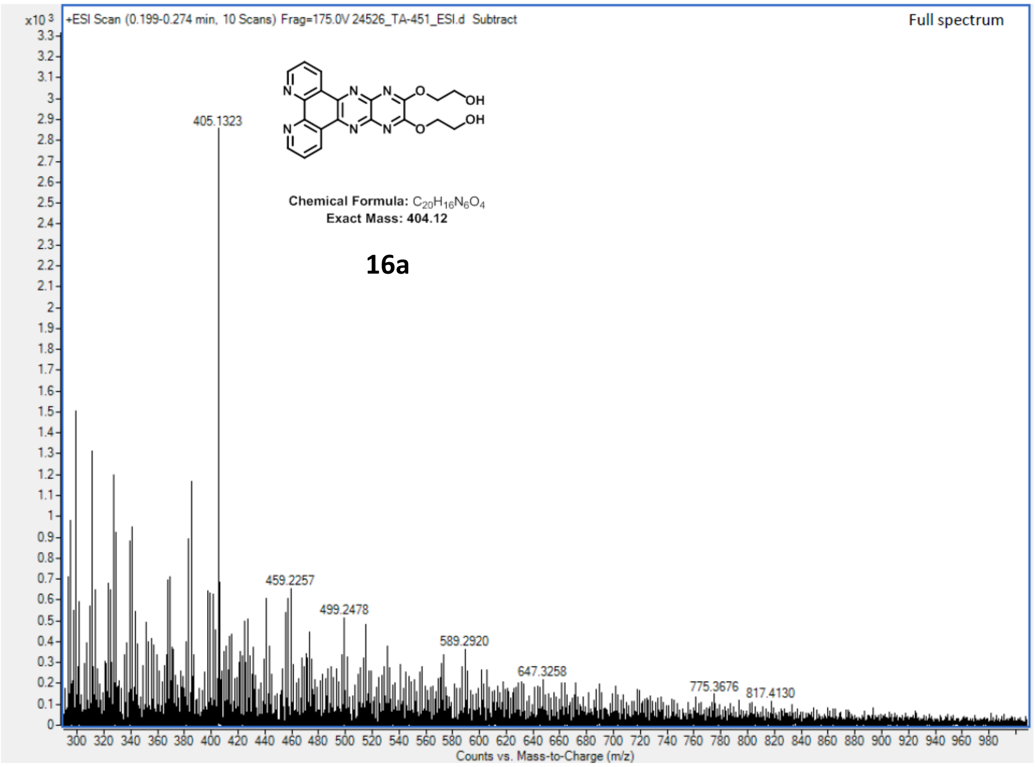
x10 3
+ESI Scan (0.199-0.274 min, 10 Scans) Frag=175.0V 24526_TA-451_ESI.d Subtract
Full spectrum
405.1323
Chemical Formula: $C_{20}H_{16}N_6O_4$
Exact Mass: 404.12
16a
459.2257
499.2478
589.2920
647.3258
775.3676
817.4130
Counts vs. Mass-to-Charge (m/z)

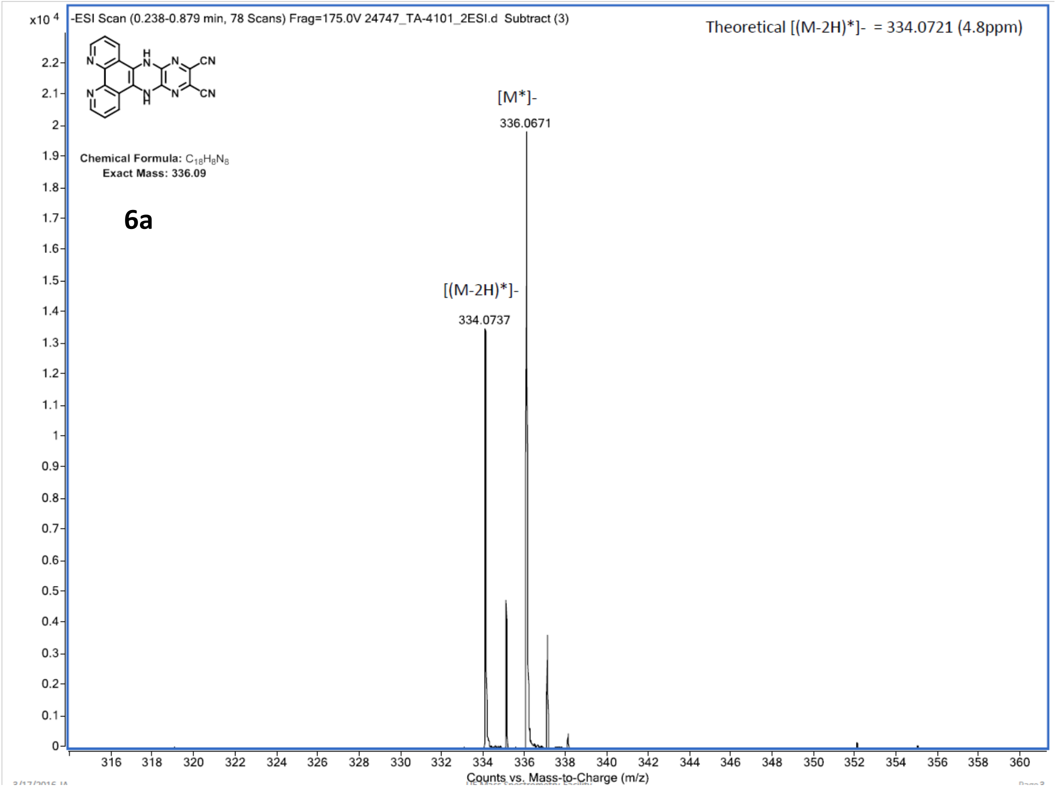
x10 4
-ESI Scan (0.238-0.879 min, 78 Scans) Frag=175.0V 24747_TA-4101_2ESI.d Subtract (3)
Theoretical [(M-2H)*]- = 334.0721 (4.8ppm)
Chemical Formula: $C_{18}H_8N_8$
Exact Mass: 336.09
6a
[M*]-
336.0671
[(M-2H)*]-
334.0737
Counts vs. Mass-to-Charge (m/z)

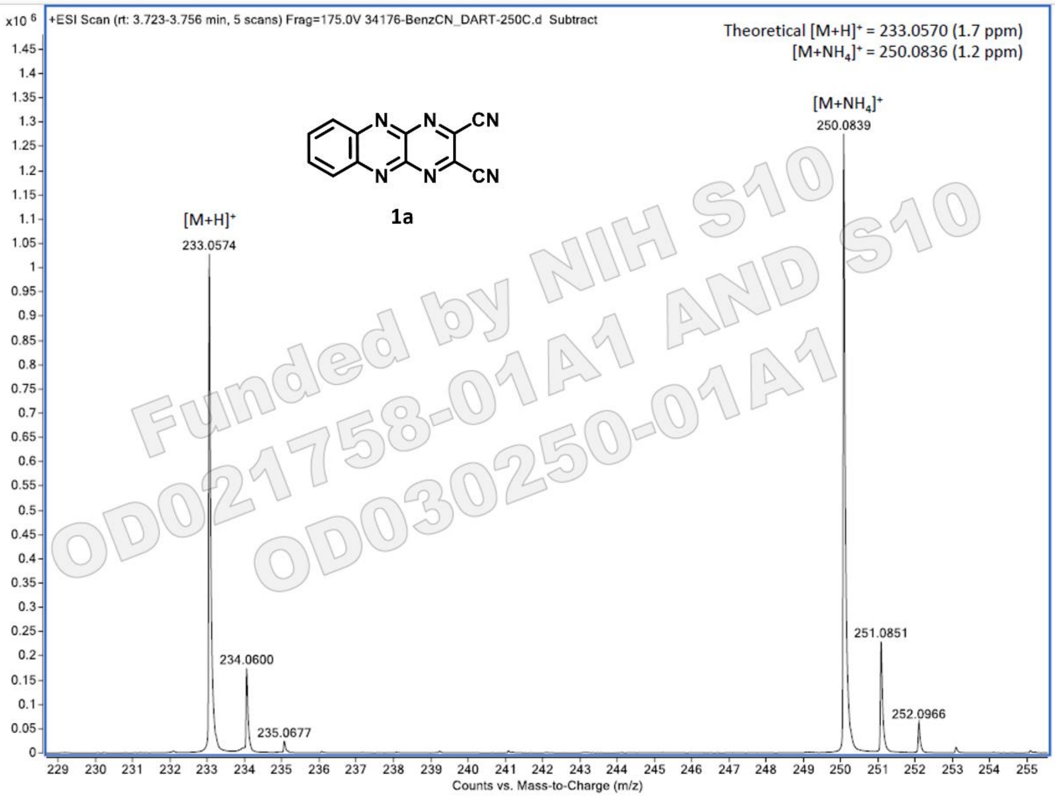
+ESI Scan (rt: 3.723-3.756 min, 5 scans) Frag=175.0V 34176-BenzCN_DART-250C.d Subtract
Theoretical [M+H]+ = 233.0570 (1.7 ppm)
[M+NH4]+ = 250.0836 (1.2 ppm)
1a
[M+H]+
233.0574
234.0600
235.0677
[M+NH4]+
250.0839
251.0851
252.0966
Counts vs. Mass-to-Charge (m/z)
Funded by NIH S10
OD021758-01A1 AND S10
OD030250-01A1

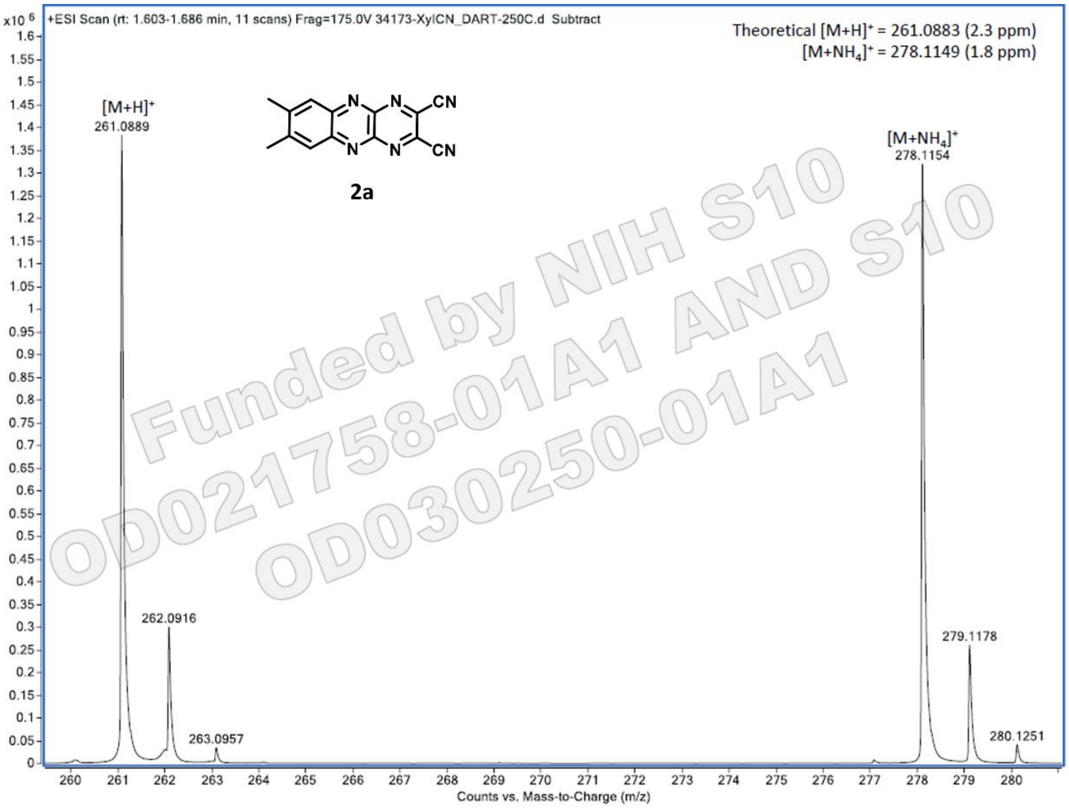
+ESI Scan (rt: 1.603-1.686 min, 11 scans) Frag=175.0V 34173-XylCN_DART-250C.d Subtract
Theoretical [M+H]+ = 261.0883 (2.3 ppm)
[M+NH4]+ = 278.1149 (1.8 ppm)
2a
[M+H]+
261.0889
262.0916
263.0957
[M+NH4]+
278.1154
279.1178
280.1251
Counts vs. Mass-to-Charge (m/z)
Funded by NIH S10
OD021758-01A1 AND S10
OD030250-01A1

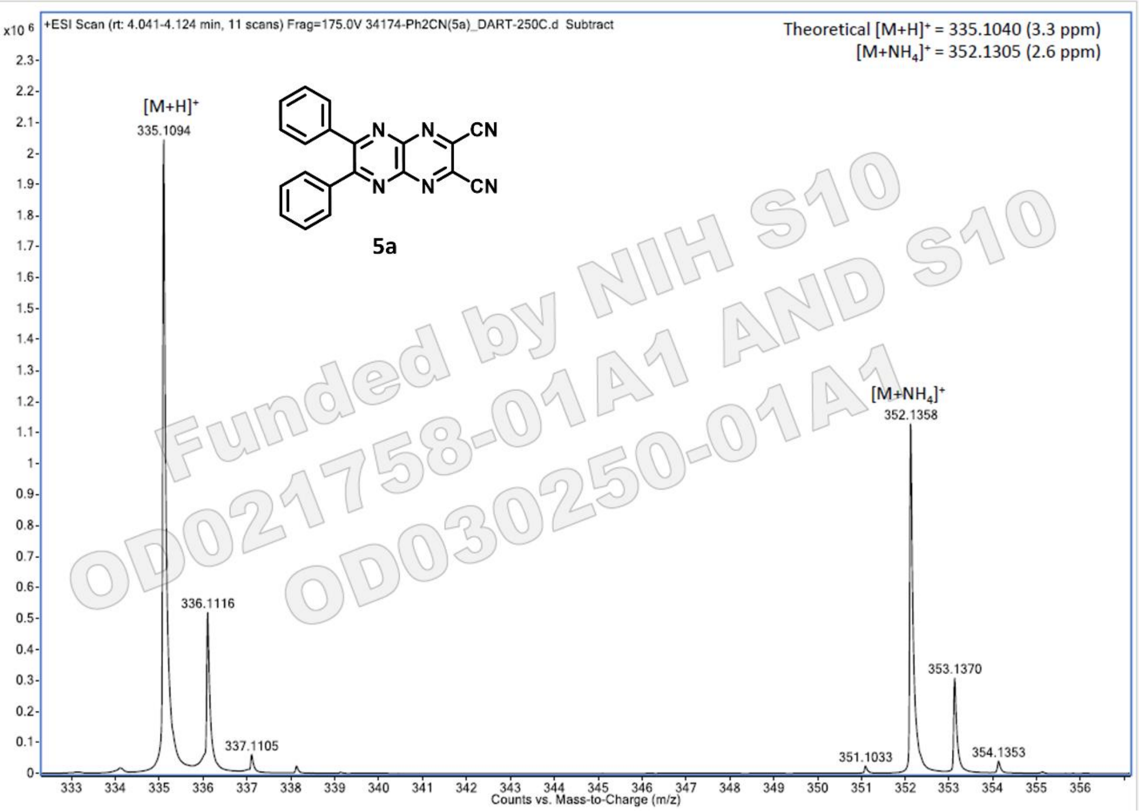

Funded by NIH S10 OD021758-01A1 AND S10 OD030250-01A1

NMR SPECTROSCOPY

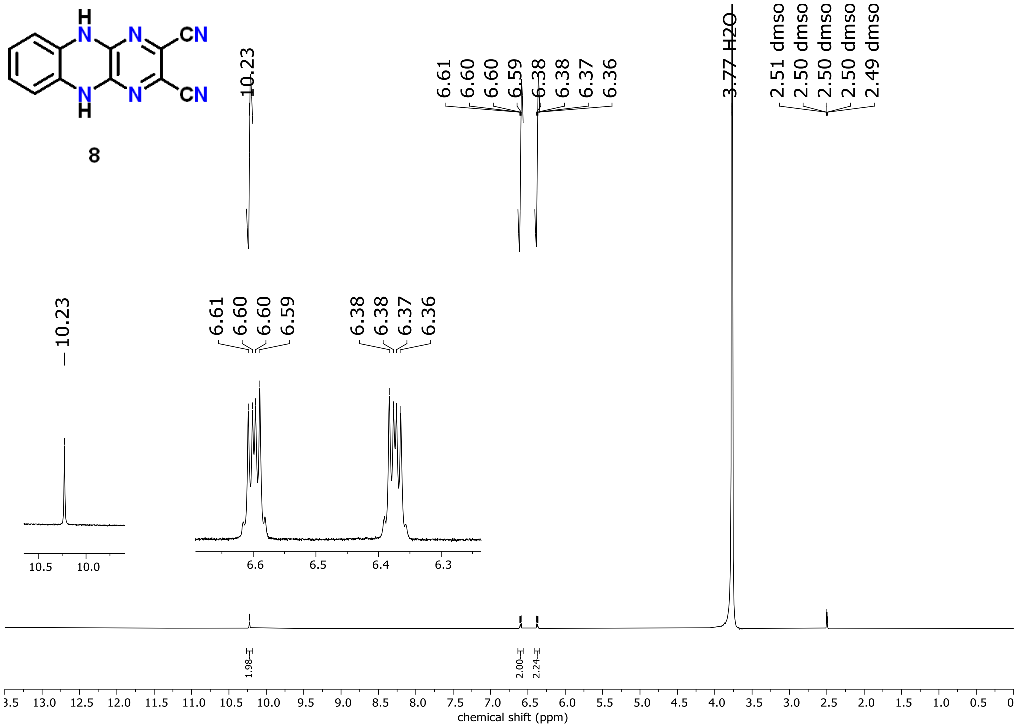

Figure S2. ^{1}H NMR spectrum of **8** in DMSO-d_6.

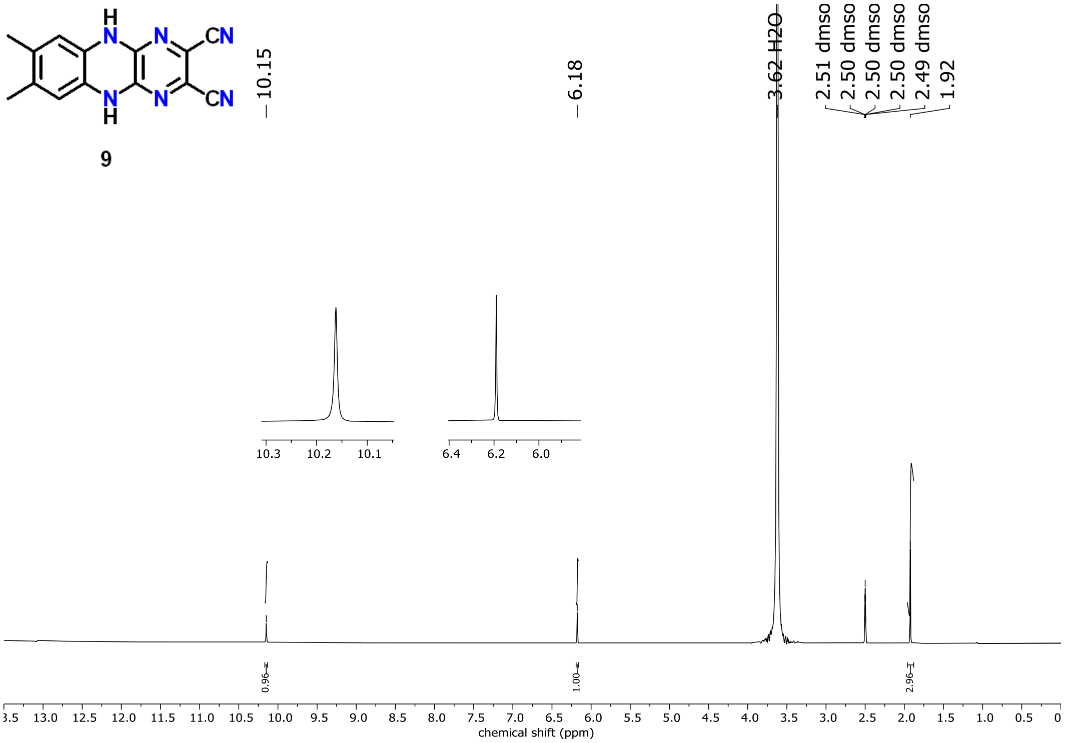

Figure S3. ^{1}H NMR spectrum of **9** in DMSO-d_6.

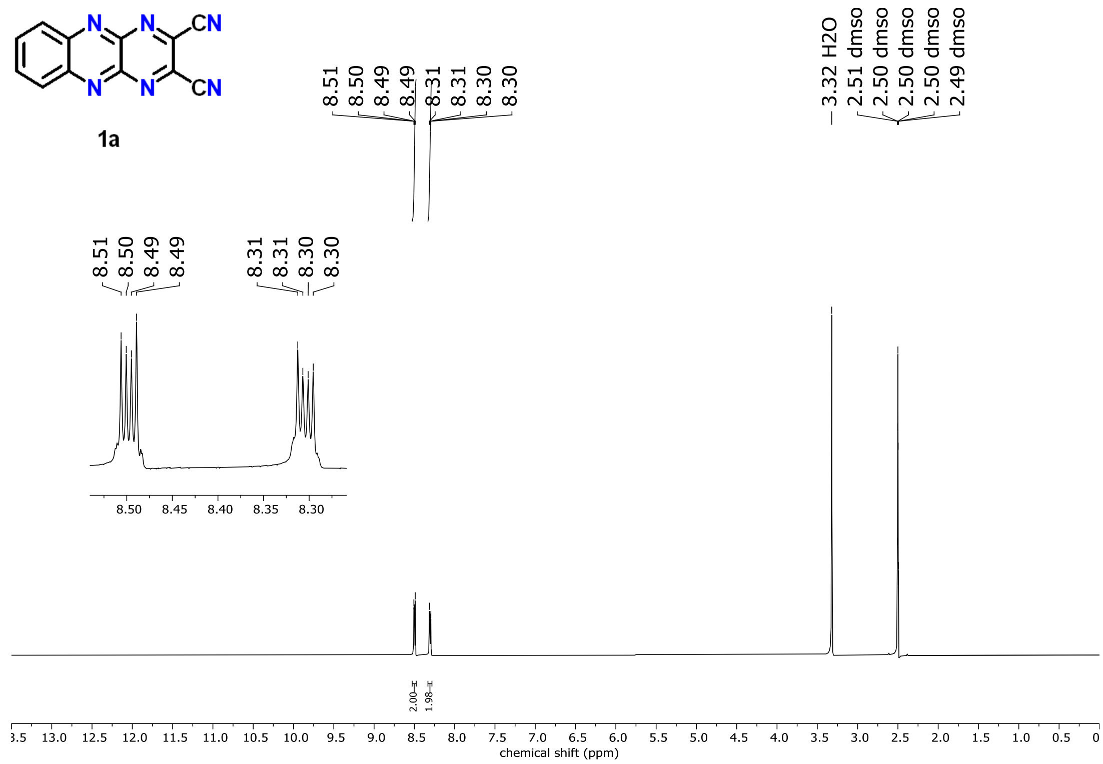

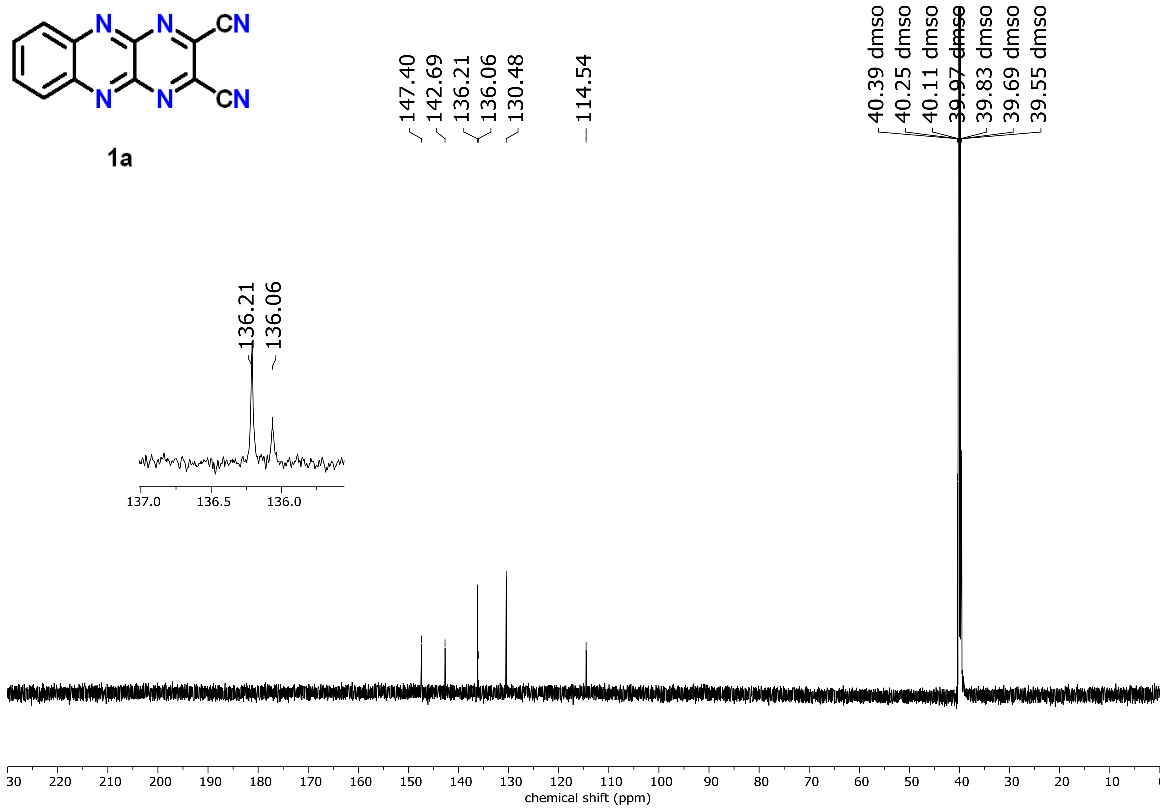

Figure S4. ^{1}H and ^{13}C NMR spectra of **1a** in DMSO-d_6.

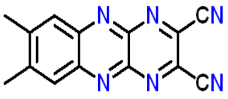

2a

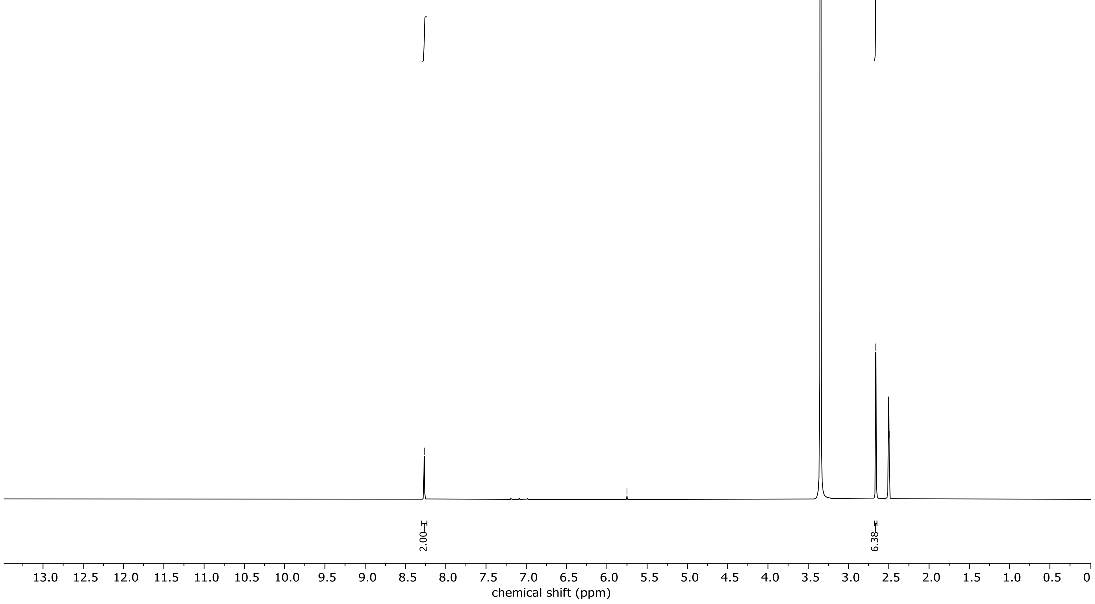

8.27
5.75 CH2Cl2
3.35 H2O
2.66
2.50 dmso
2.50 dmso
2.50 dmso
2.50 dmso
2.00
6.38
13.0 12.5 12.0 11.5 11.0 10.5 10.0 9.5 9.0 8.5 8.0 7.5 7.0 6.5 6.0 5.5 5.0 4.5 4.0 3.5 3.0 2.5 2.0 1.5 1.0 0.5 0
chemical shift (ppm)

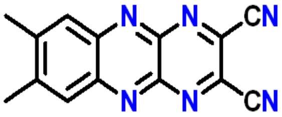

2a

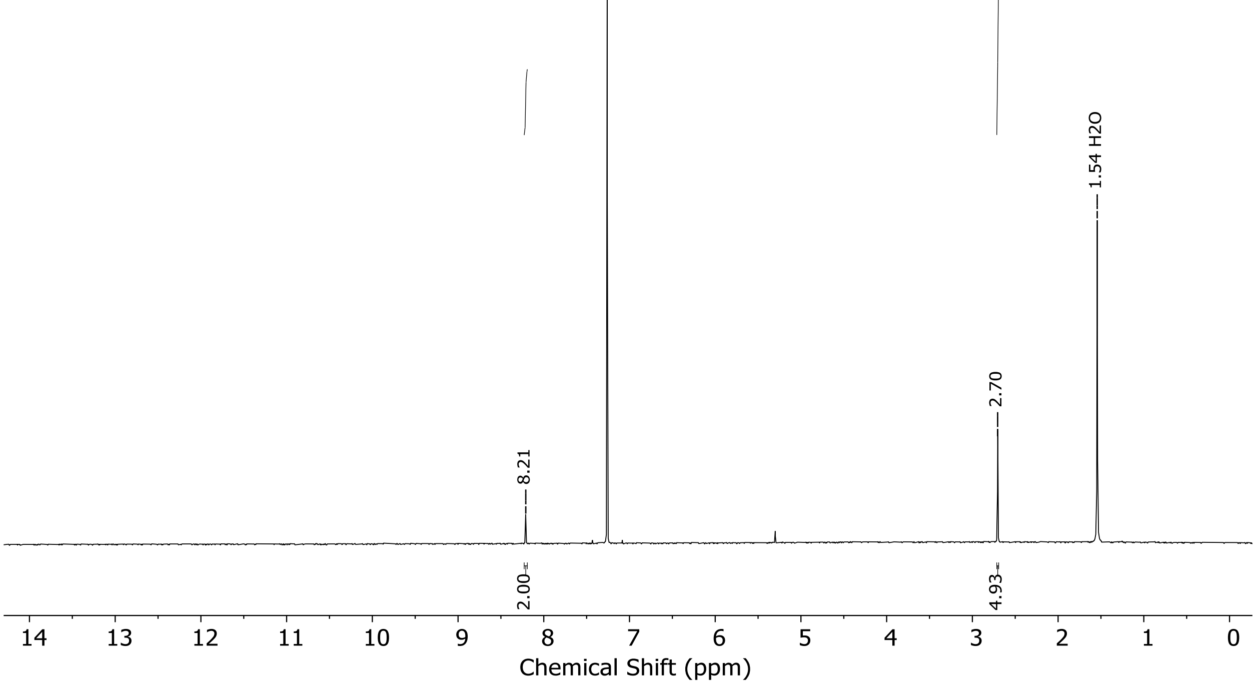

7.26 CDCl3
1.54 H2O
2.70
8.21
2.00
4.93
14 13 12 11 10 9 8 7 6 5 4 3 2 1 0
Chemical Shift (ppm)

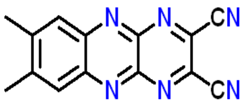

2a

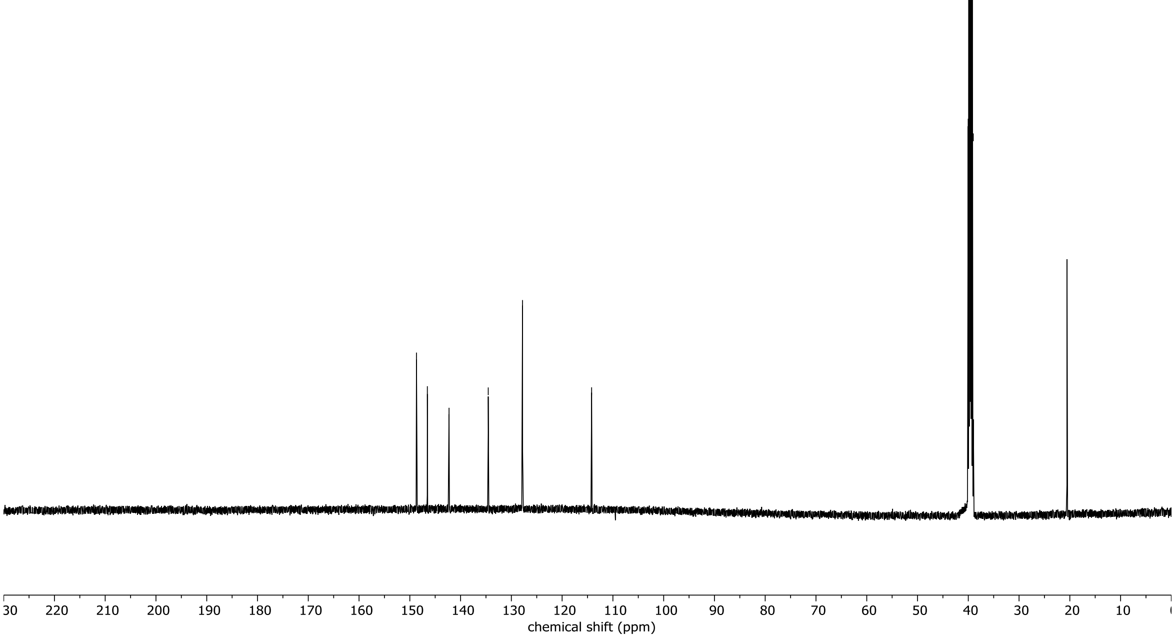

Figure S5. ^{1}H in DMSO-d_6, 1H in $CDCl_3$ and ^{13}C NMR spectra of **2a** in DMSO-d_6, respectively.

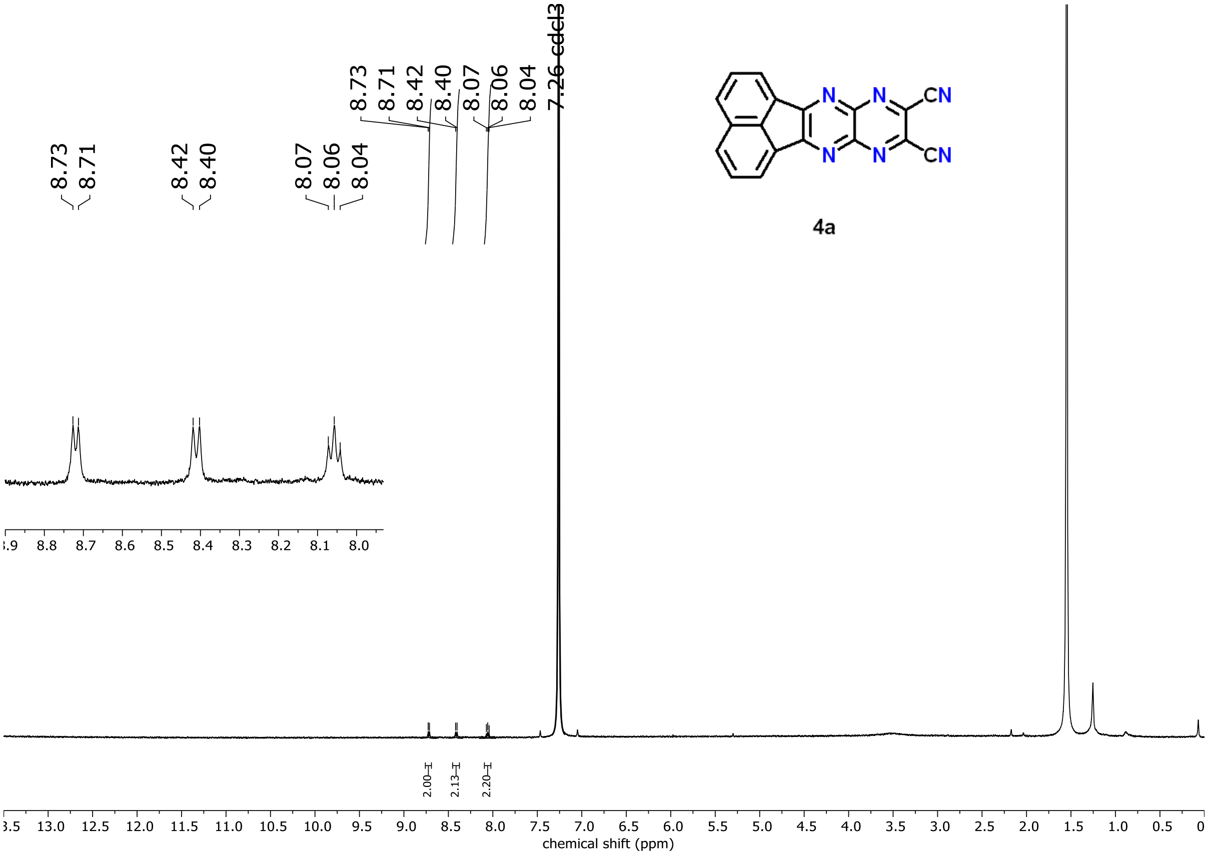

Figure S6. ^{1}H NMR spectrum of **4a** in $CDCl_3$.

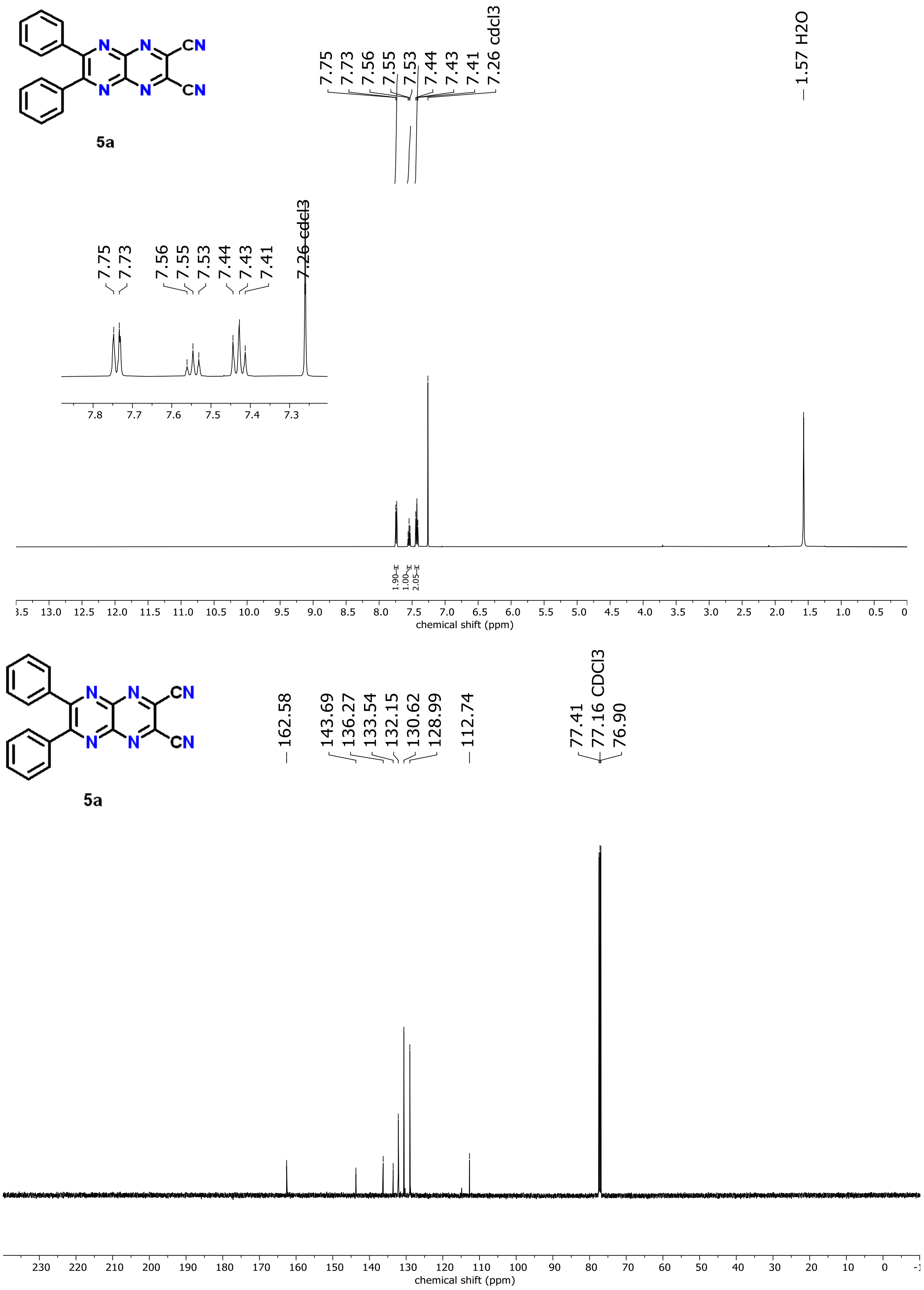

Figure S7. ^{1}H NMR and ^{13}C NMR spectra of **5a** in $CDCl_3$.

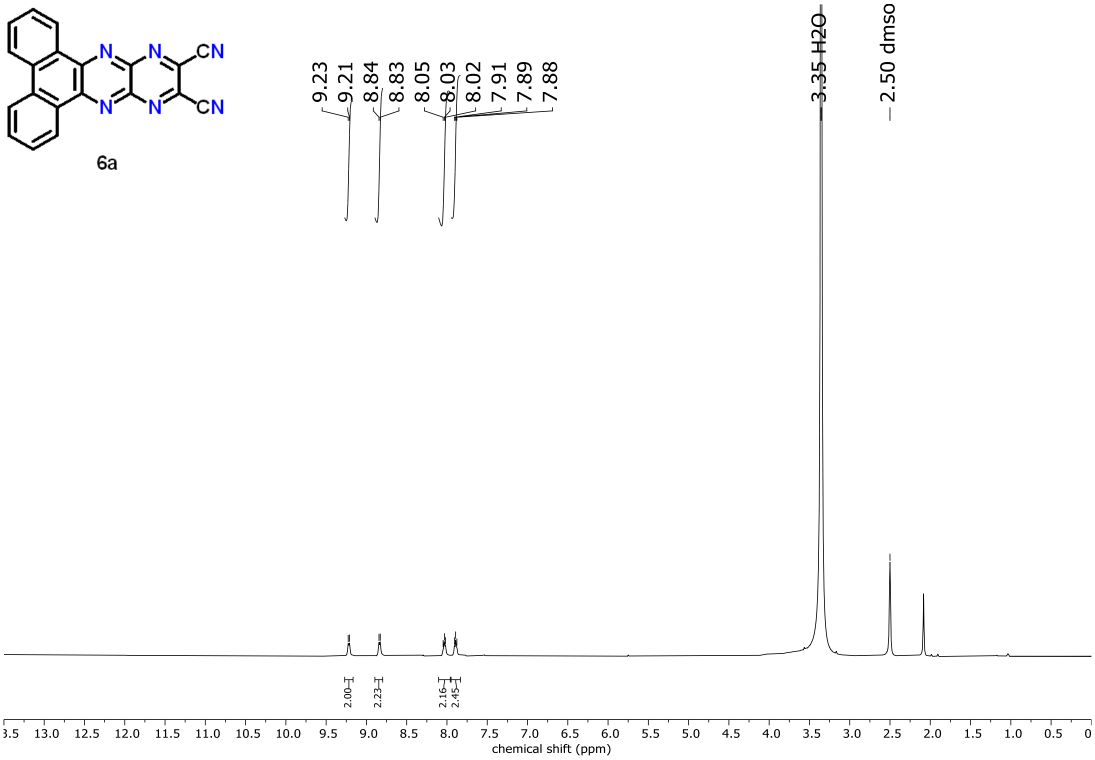

Figure S8. ^{1}H NMR spectrum of **6a** in $CDCl_3$.

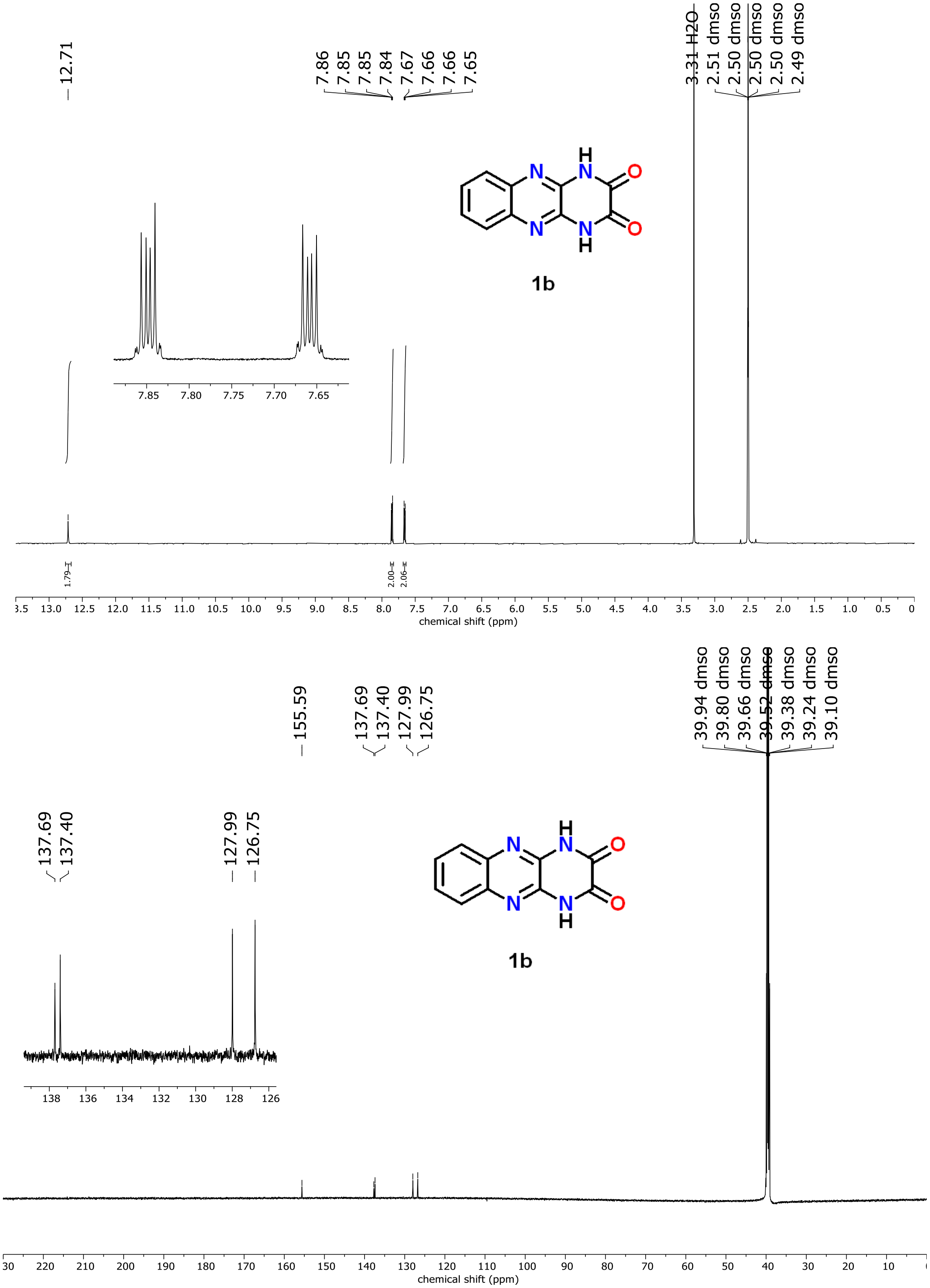

Figure S9. ^{1}H and ^{13}C NMR spectra of **1b** in DMSO-d_6.

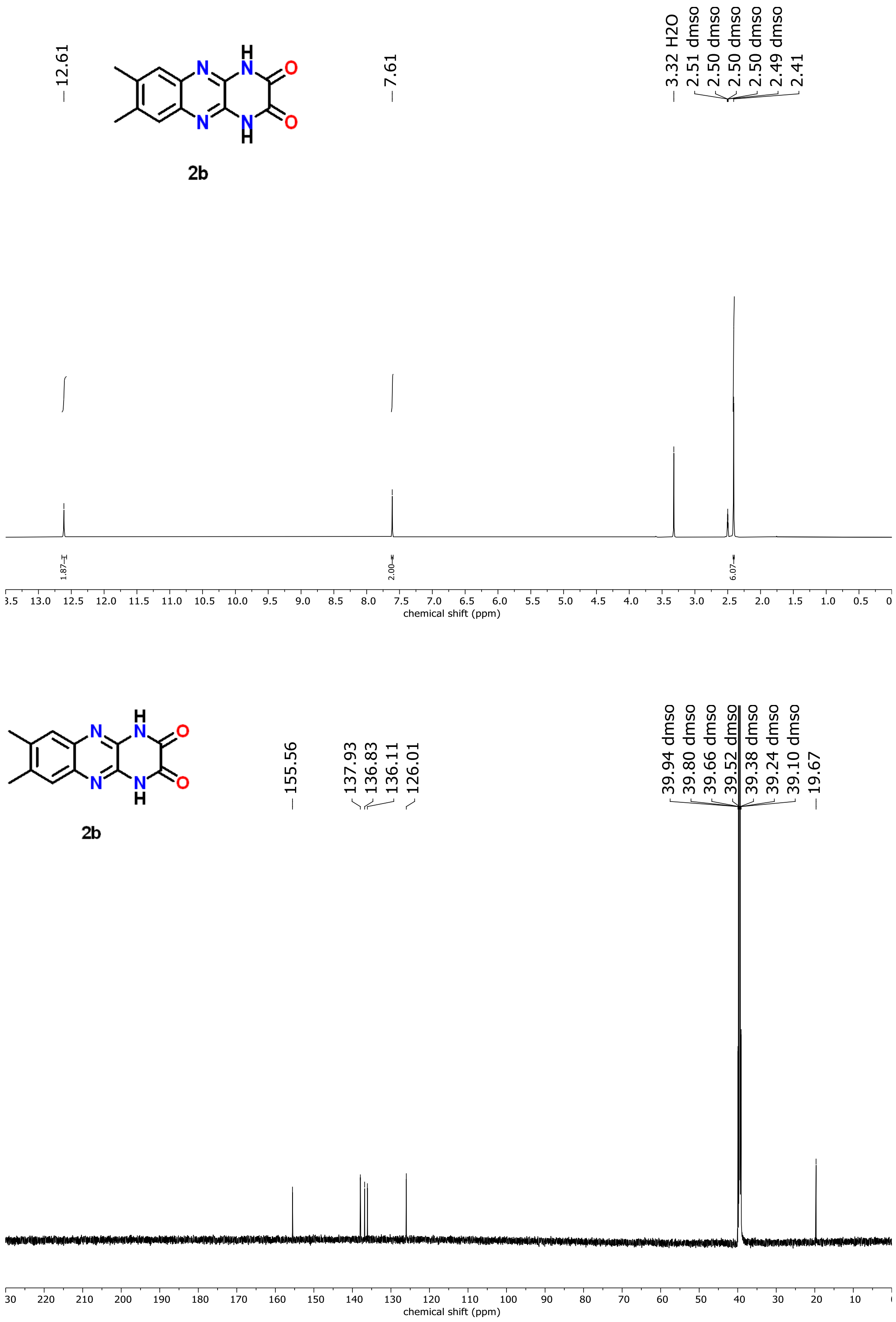

Figure S10. ^{1}H and ^{13}C NMR spectra of **2b** in DMSO-d_6.

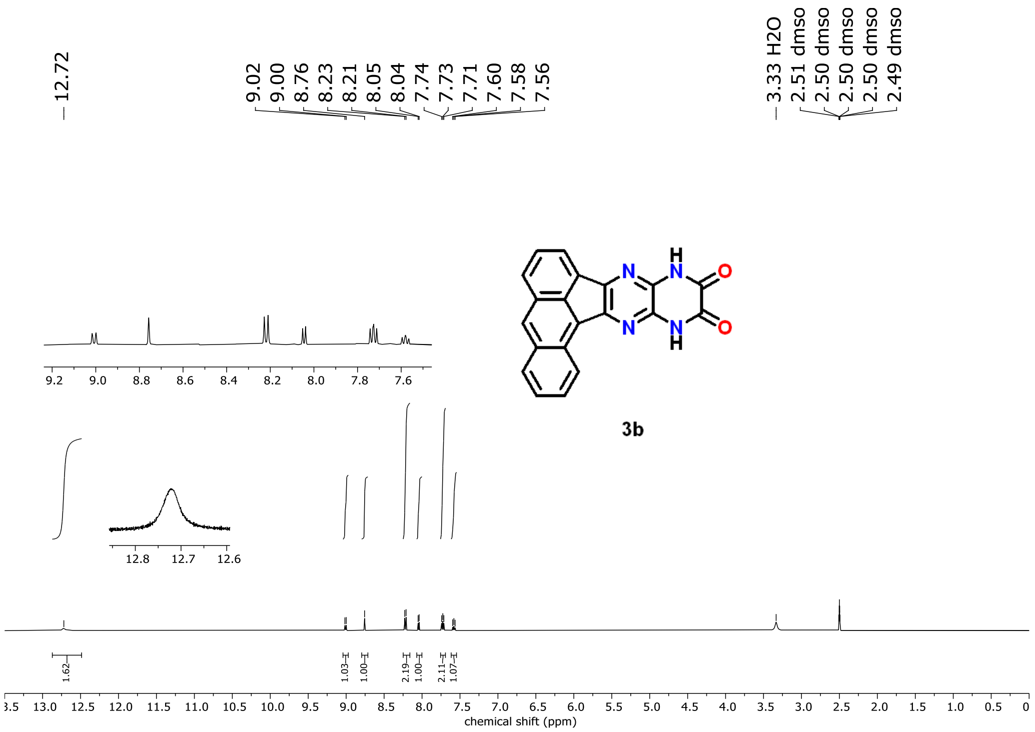

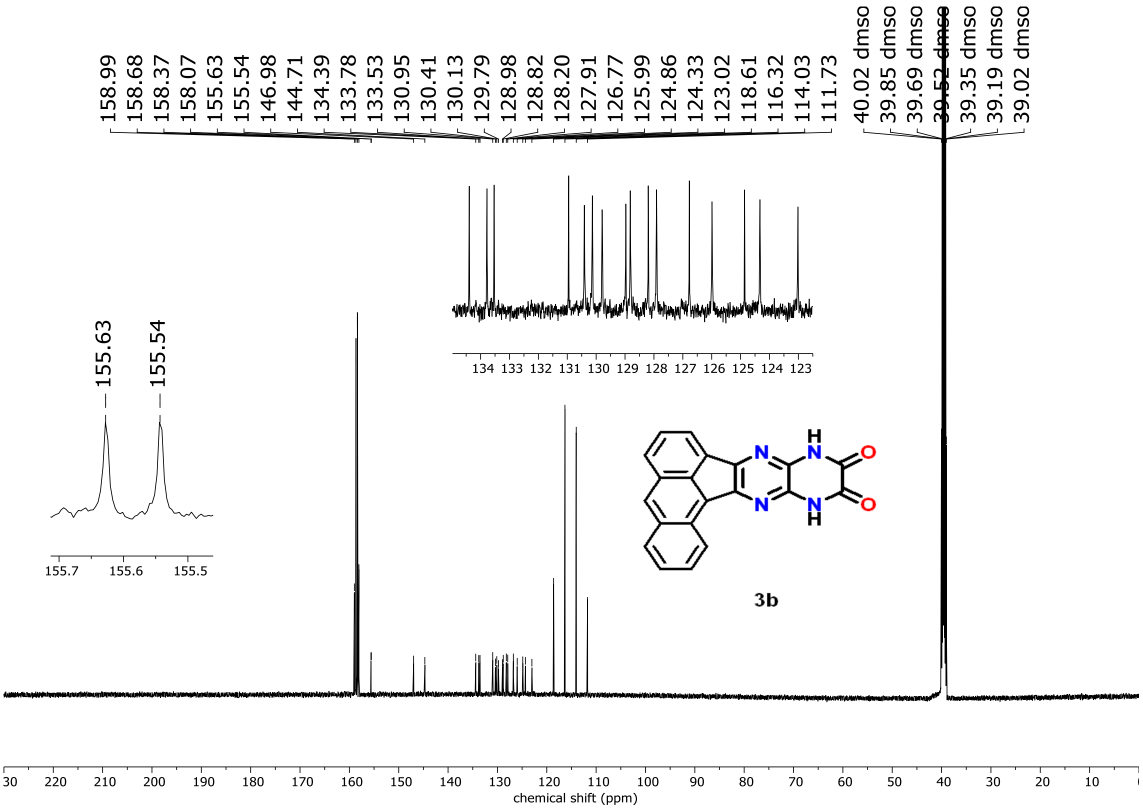

Figure S11. ^{1}H and ^{13}C NMR spectra of **3b** in DMSO-d_6.

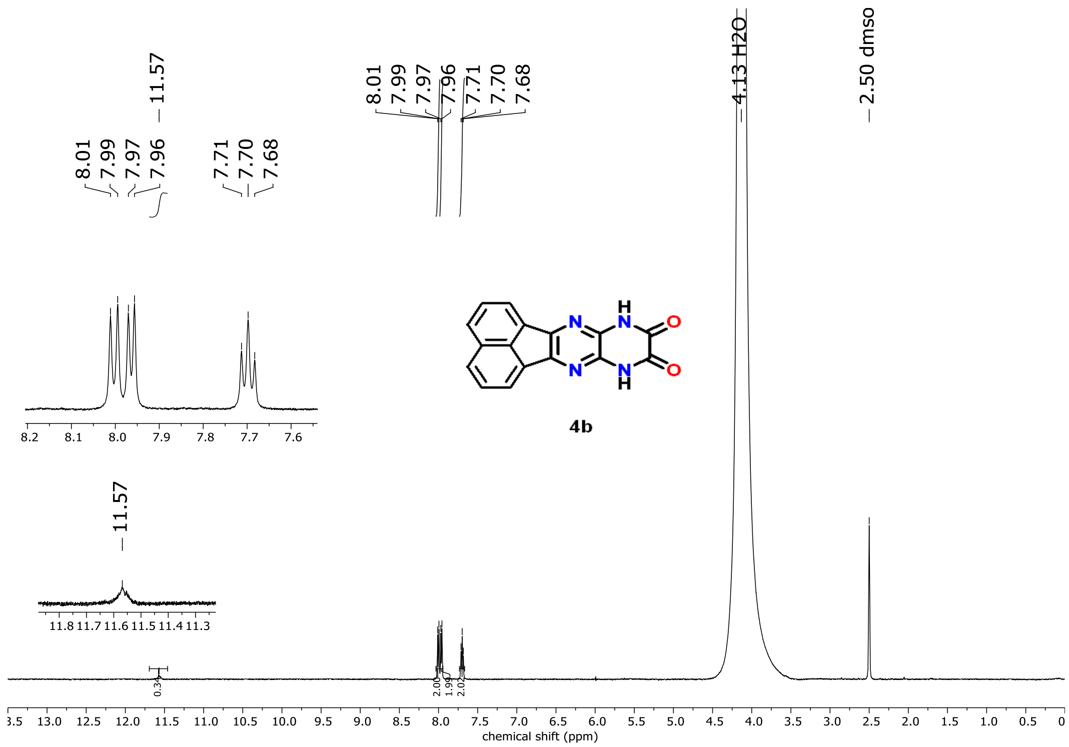

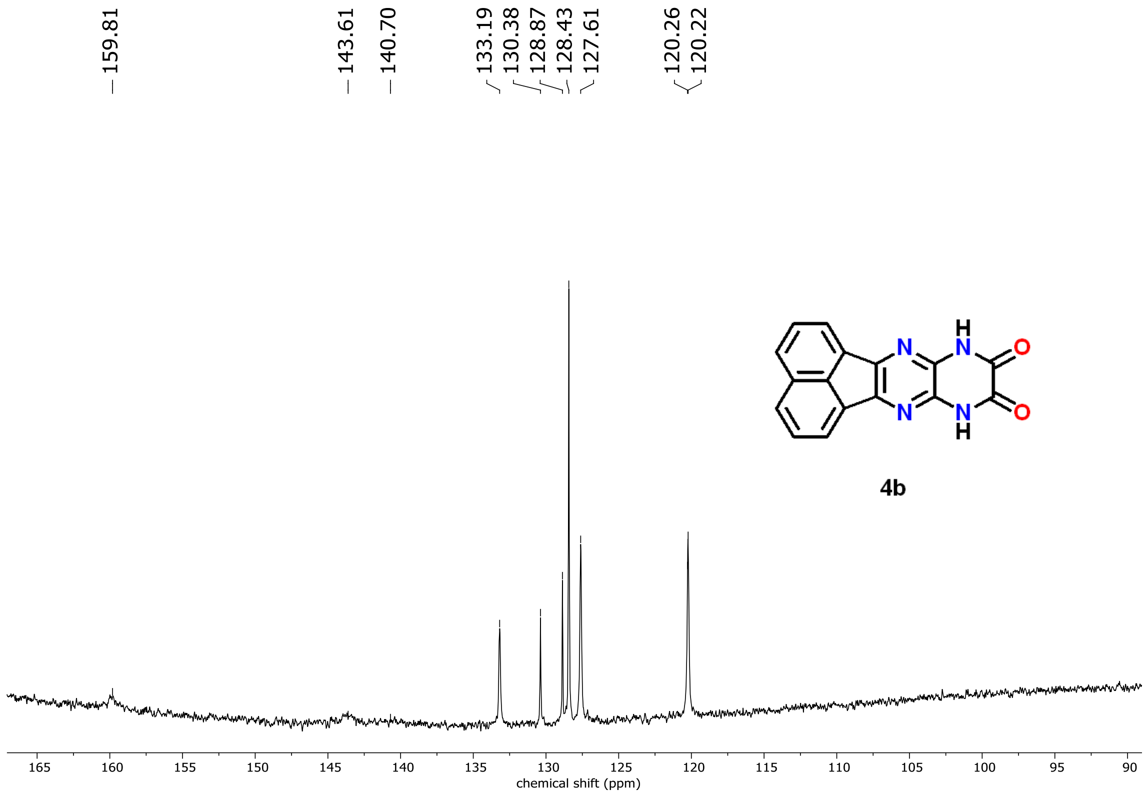

Figure S12. ^{1}H and ^{13}C NMR spectra of **4b** in DMSO-d_6.

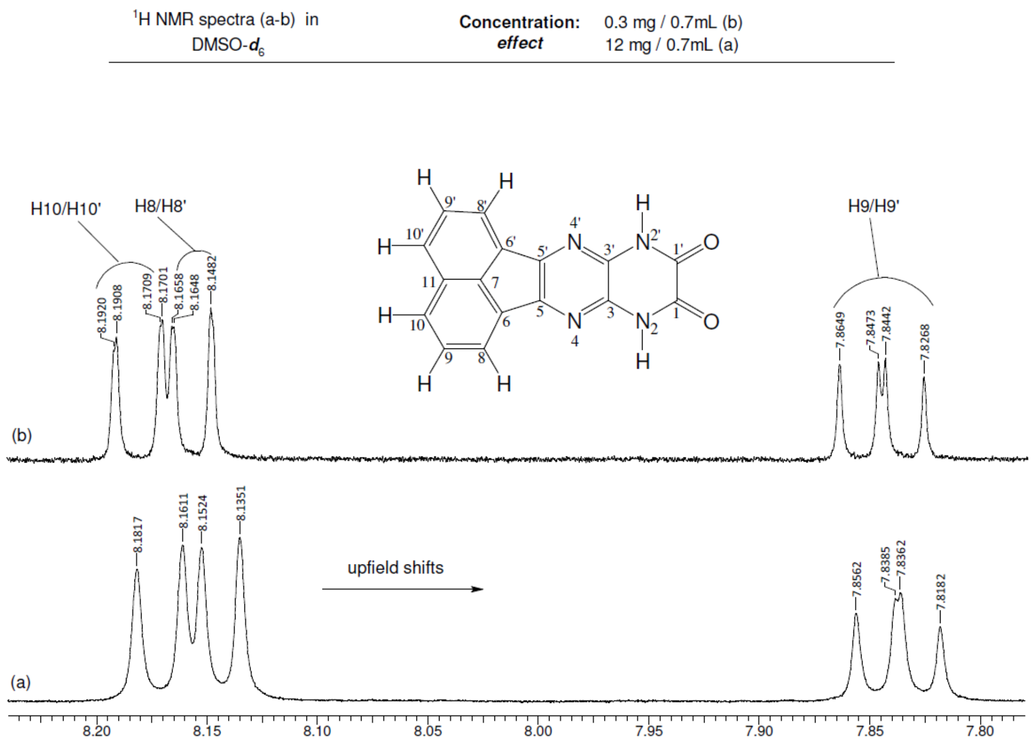

Figure S13. ^{1}H NMR spectrum of **4b** in DMSO-d_6 after heating to 200 °C for 7 days.

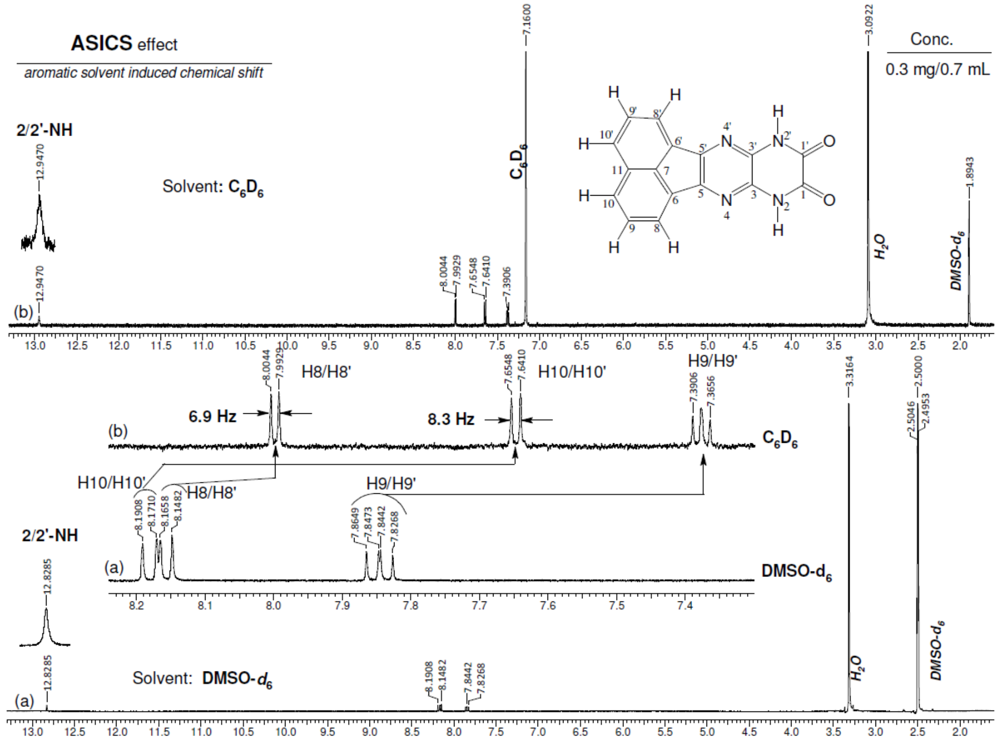

Figure S14. ^{1}H NMR ASICS of **4b** in DMSO-d_6 after heating to 200 °C for 7 days.

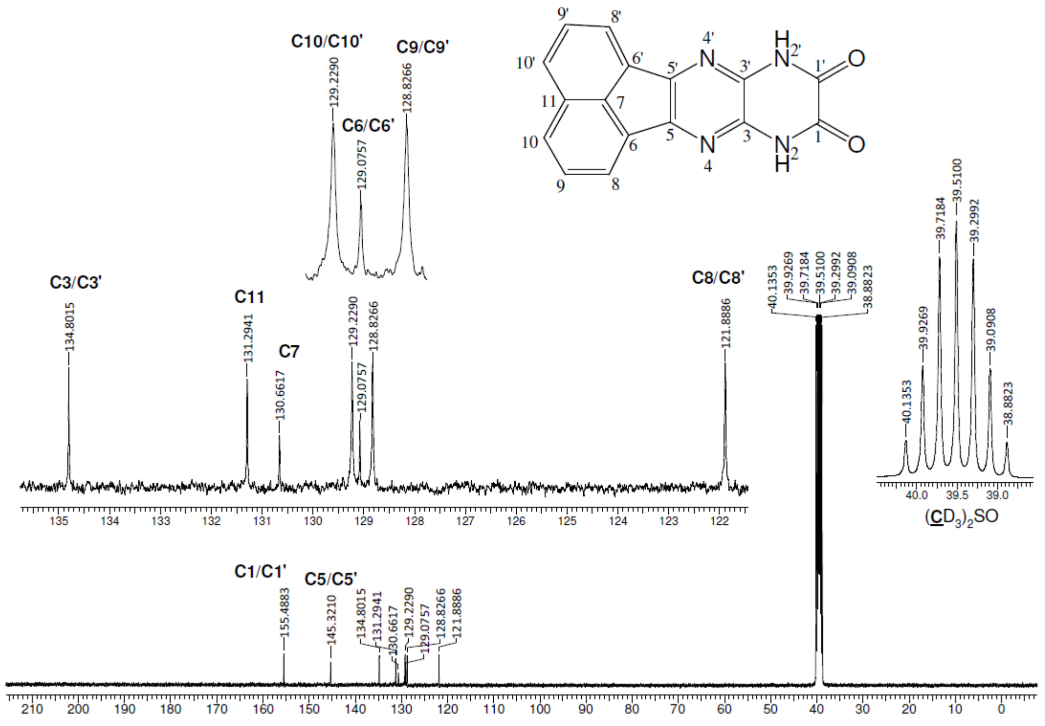

Figure S15. ^{13}C NMR spectrum of **4b** in DMSO-d_6 after heating to 200 °C for 7 days.

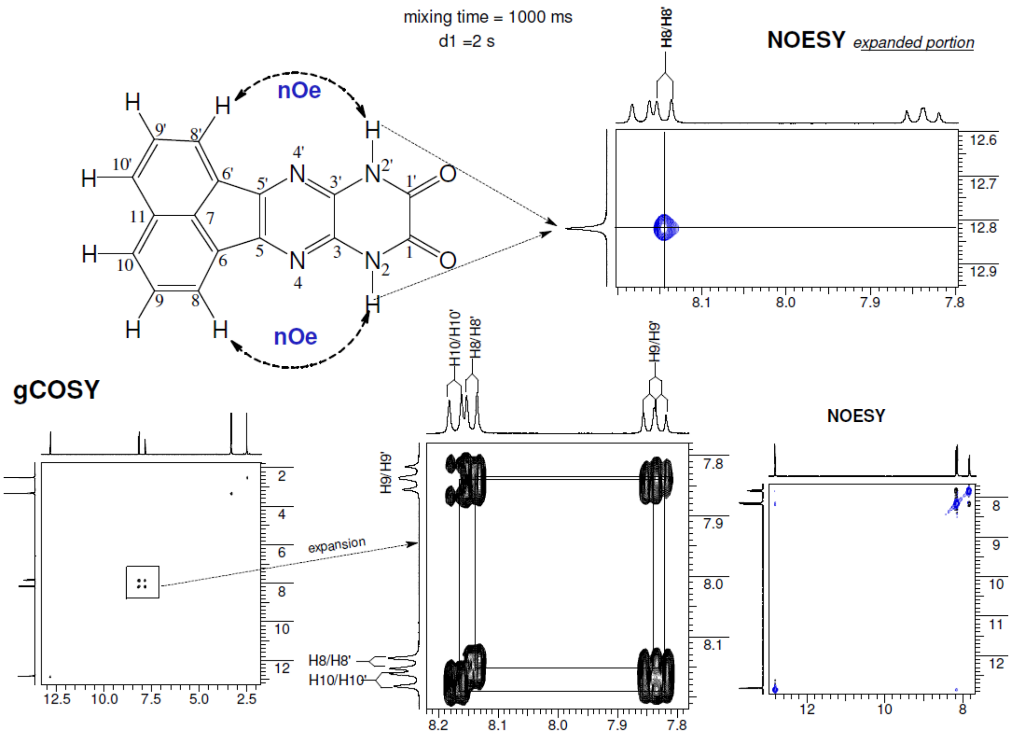

Figure S16. NOESY spectrum of **4b** in DMSO-d_6 after heating to 200 °C for 7 days.

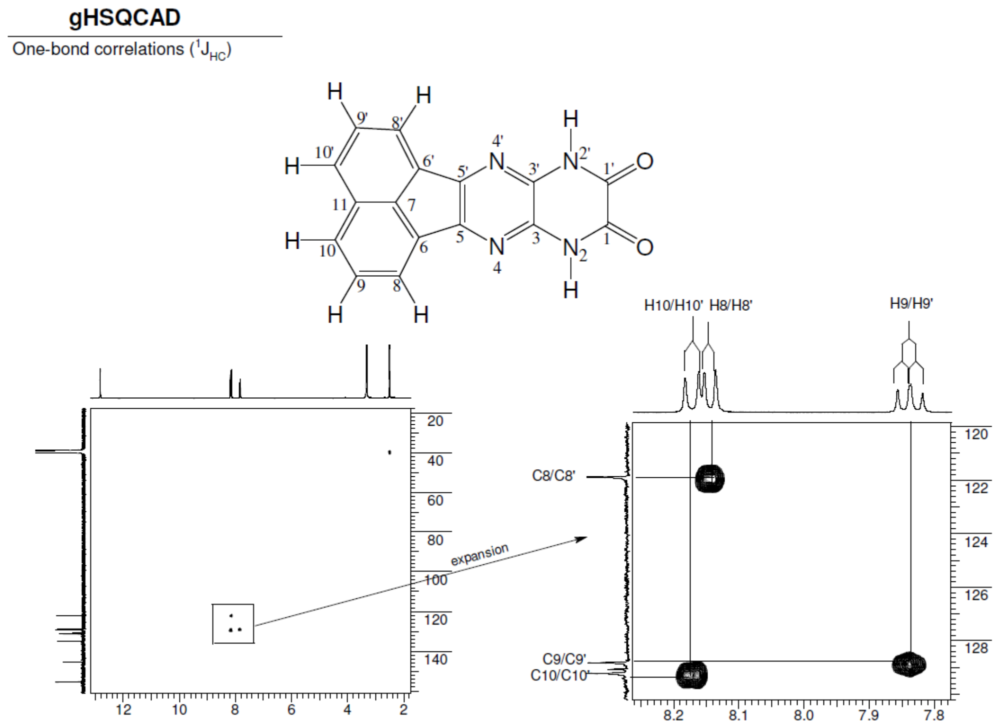

Figure S17. gHSQCAD spectrum of **4b** in DMSO-d_6 after heating to 200 °C for 7 days.

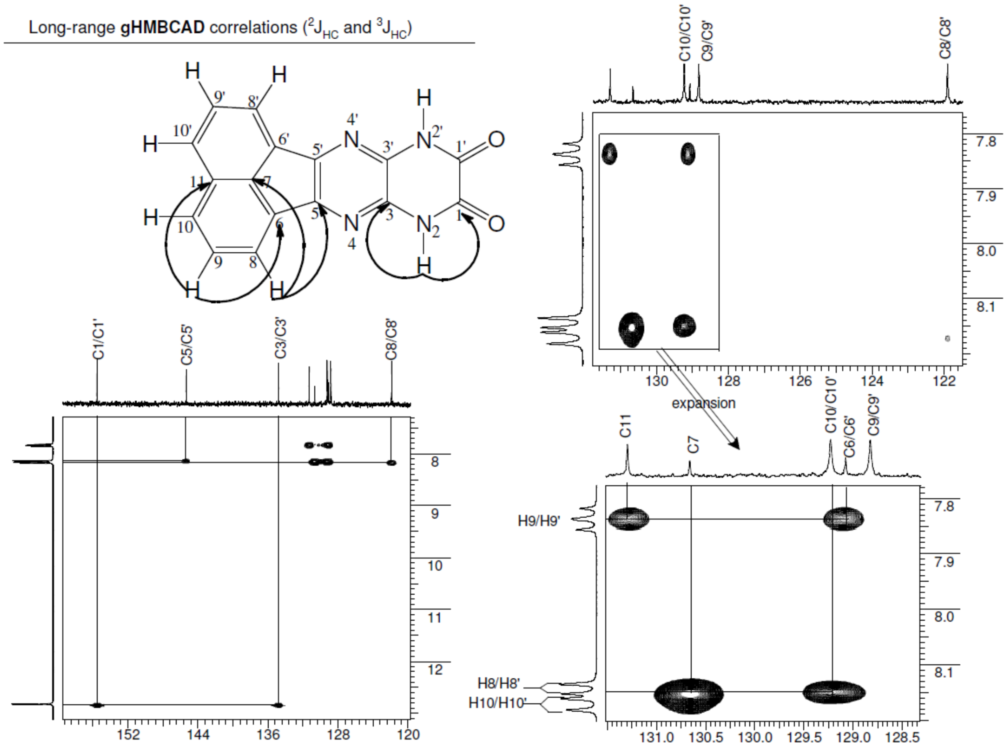

Figure S18. gHMBCAD spectrum of **4b** in DMSO-d_6 after heating to 200 °C for 7 days.

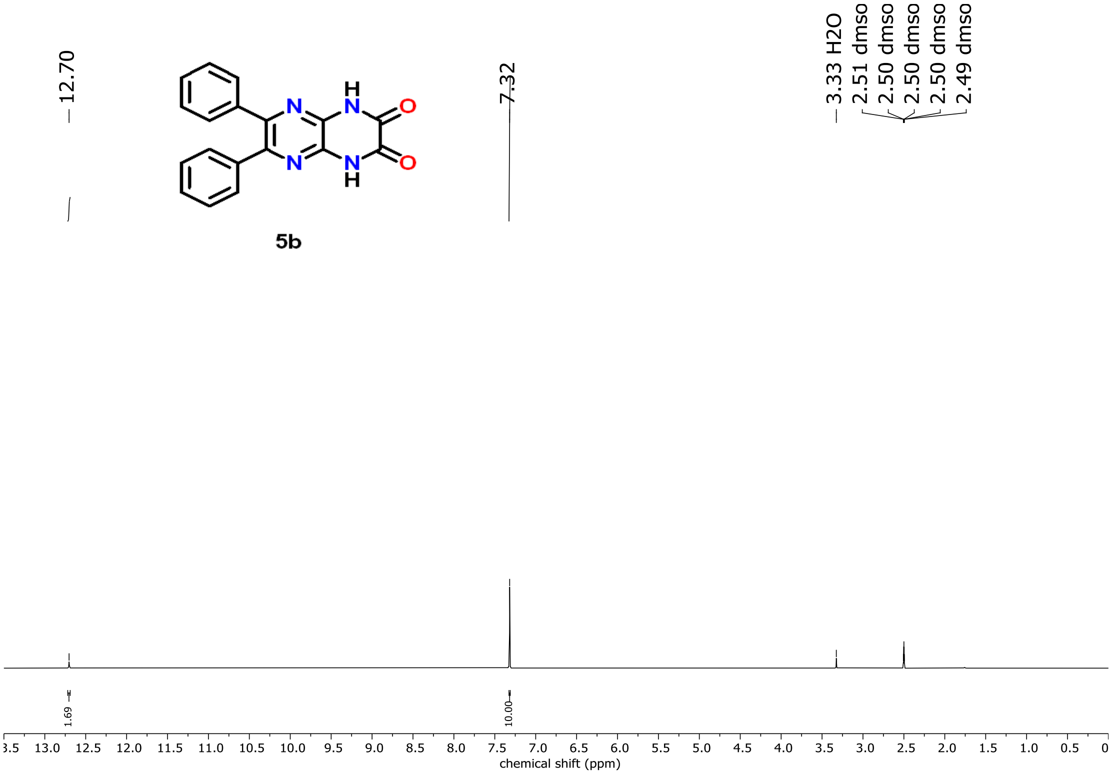

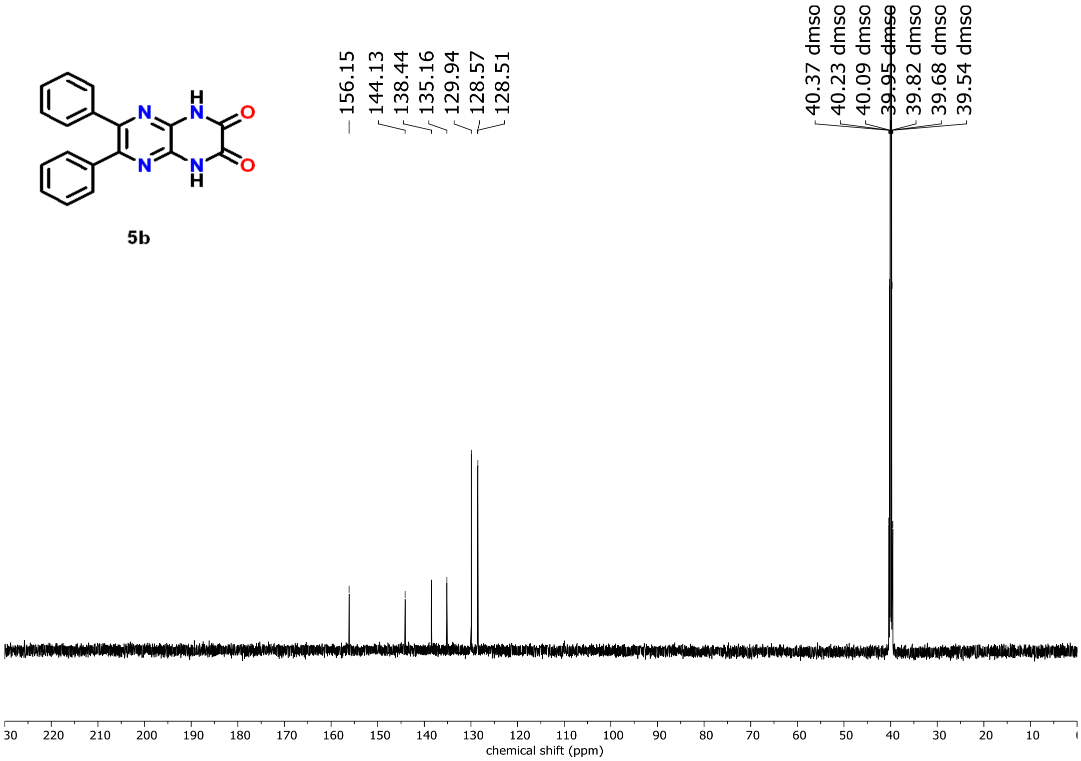

Figure S19. ^{1}H and ^{13}C NMR spectra of **5b** in DMSO-d_6.

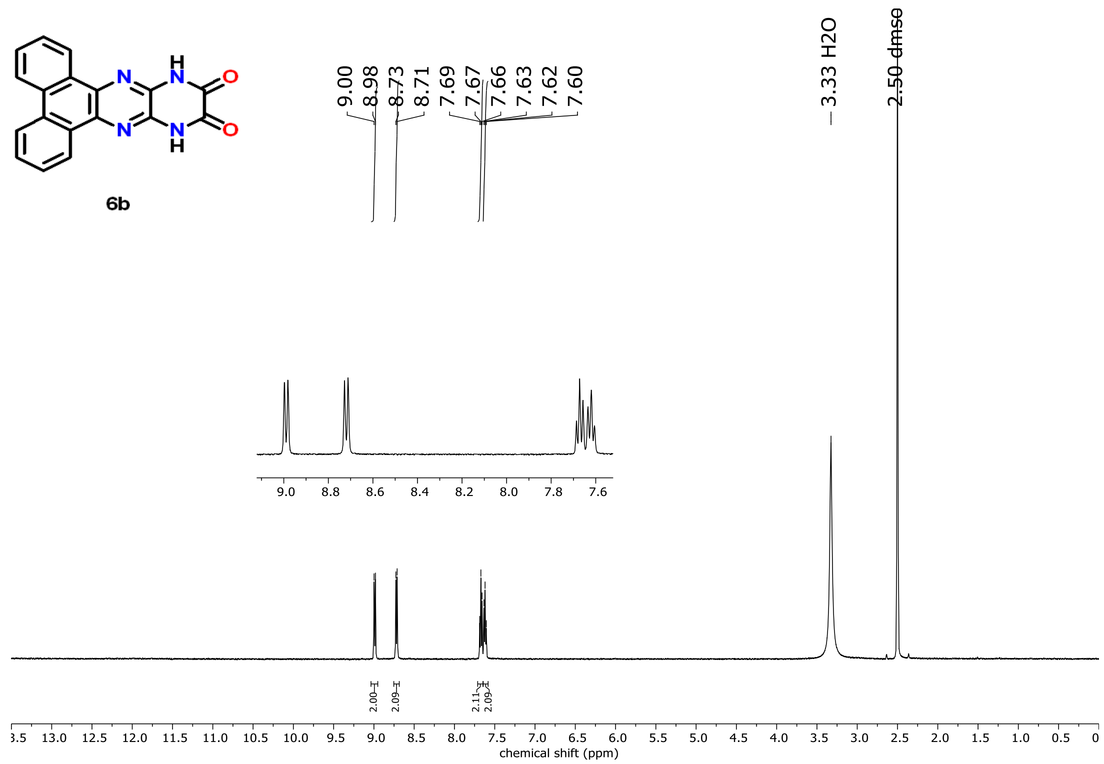

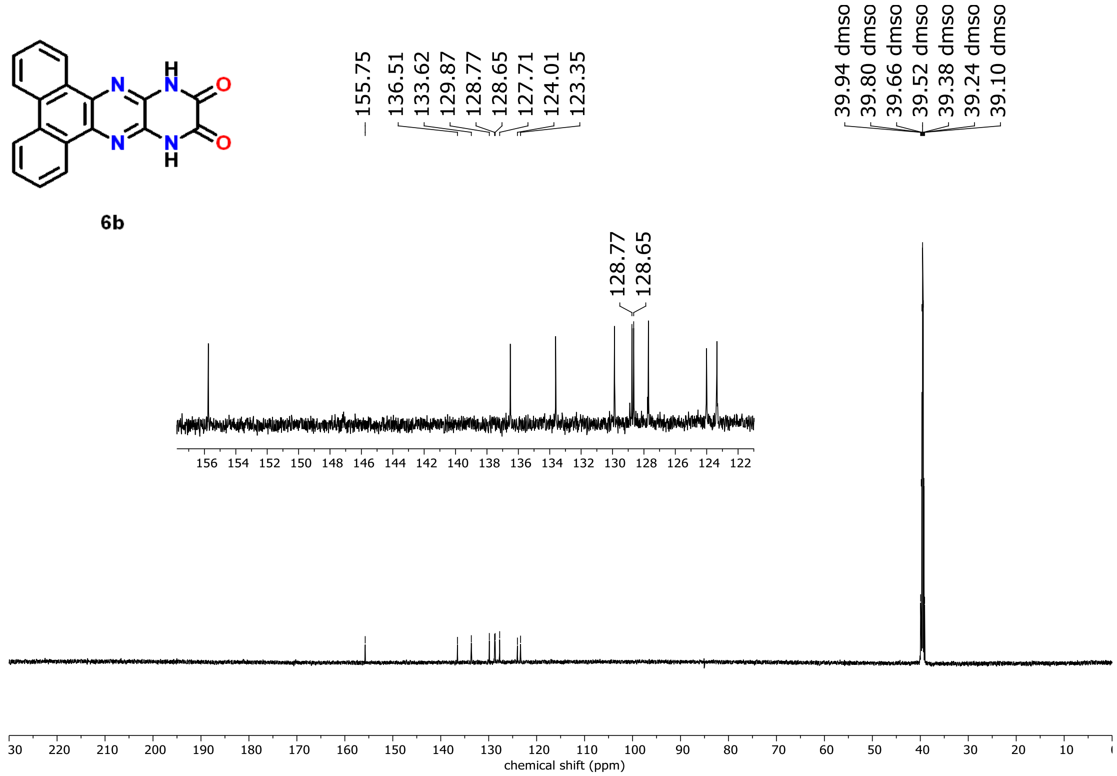

Figure S20. ^{1}H and ^{13}C NMR spectra of **6b** in DMSO-d_6.

^{1}H-^{1}H gCOSY spectrum

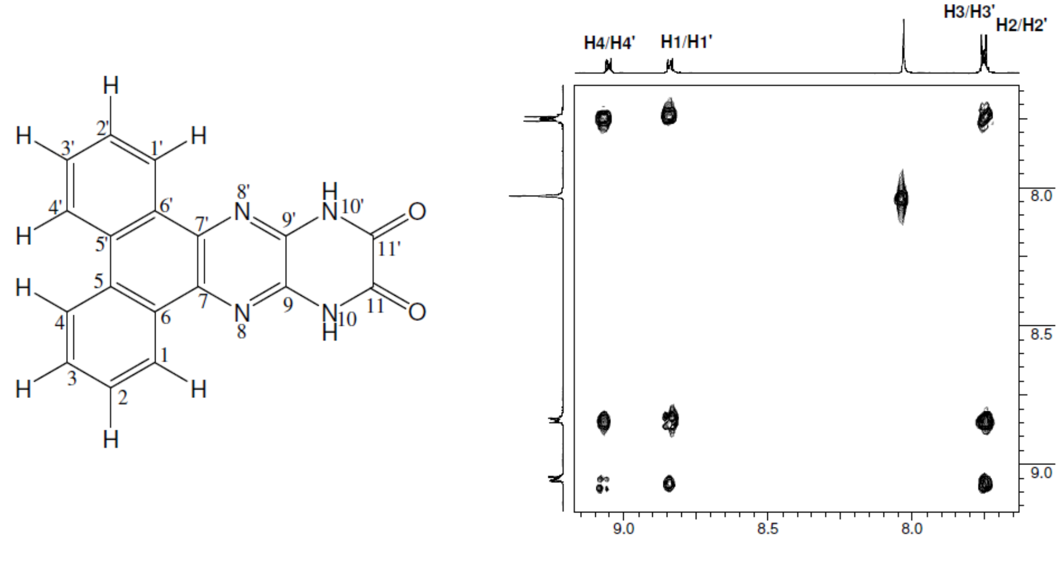

Figure S21. gCOSY spectrum of **6b** in DMSO-d_6.

One-bond ($^{1}J_{HC}$) gHSQCAD correlations

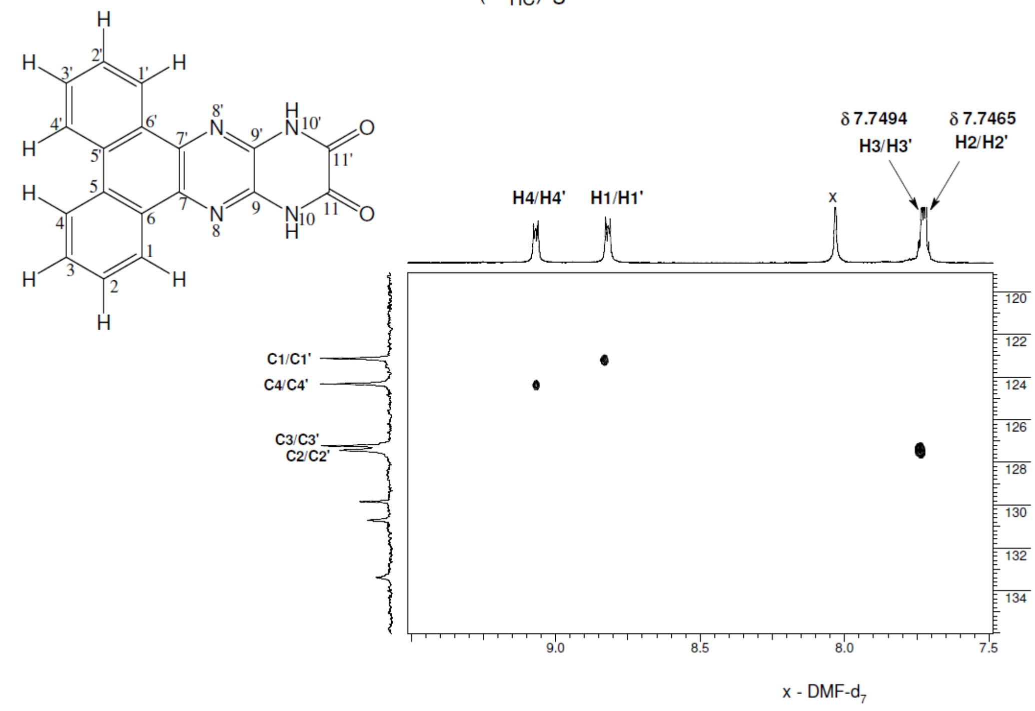

Figure S22. gHSQCAD spectrum of **6b** in DMSO-d_6.

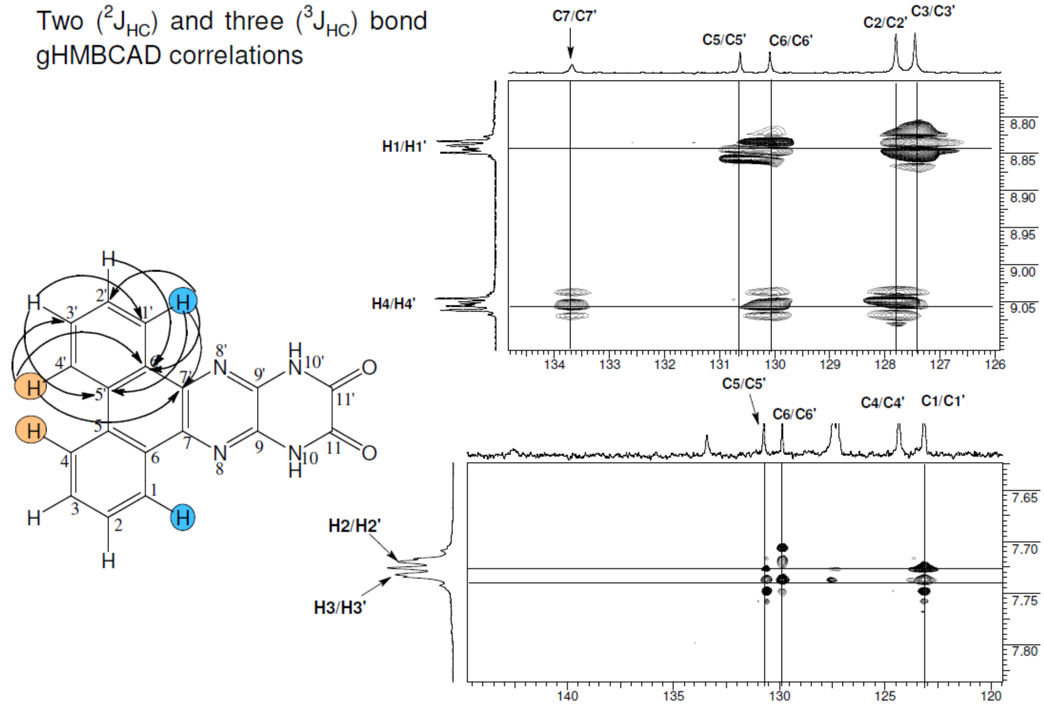

Figure S23. gHMBCAD spectrum of **6b** in DMSO-d_6.

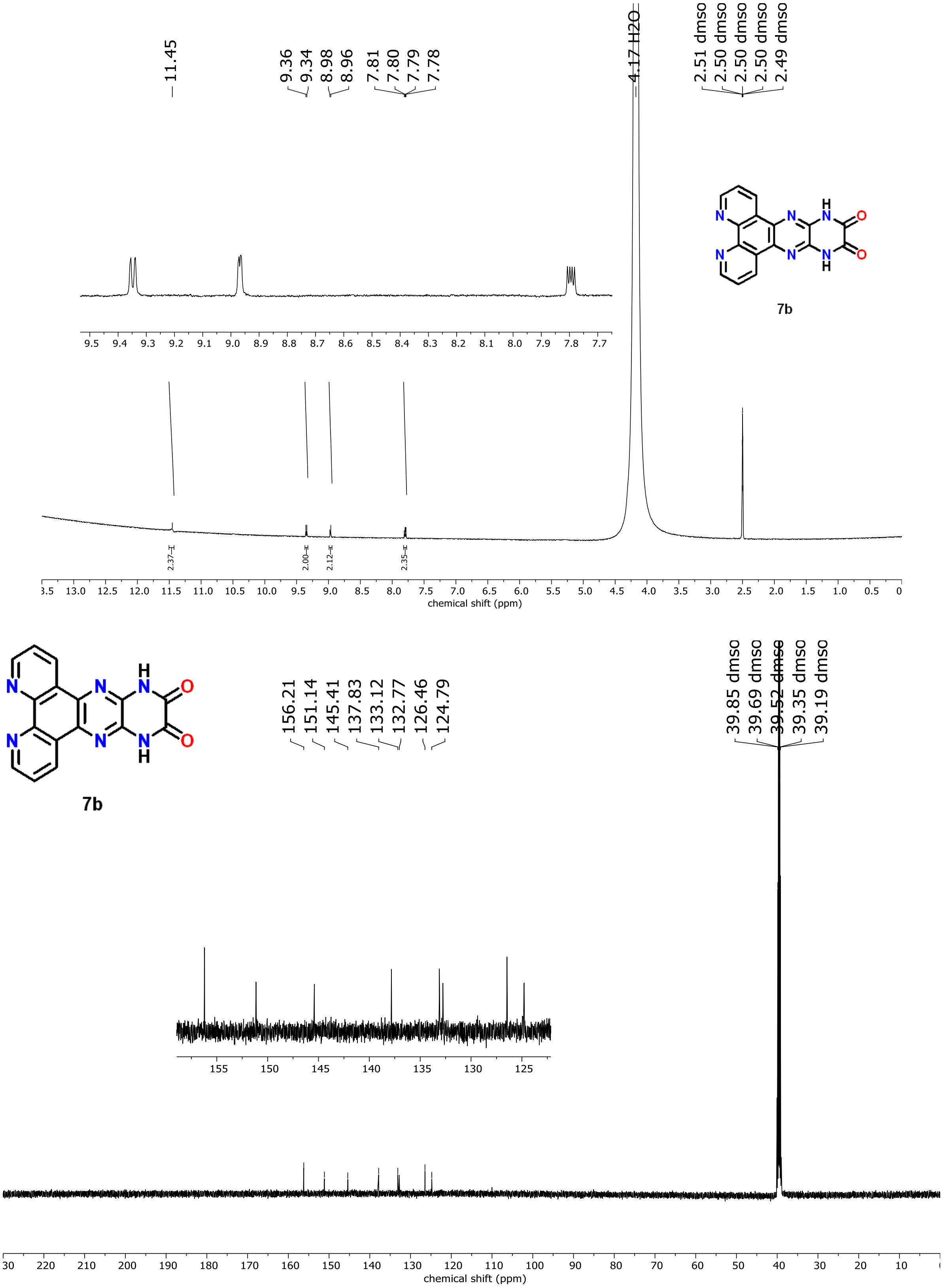

Figure S24. ^{1}H and ^{13}C NMR spectra of **7b** in DMSO-d_6.

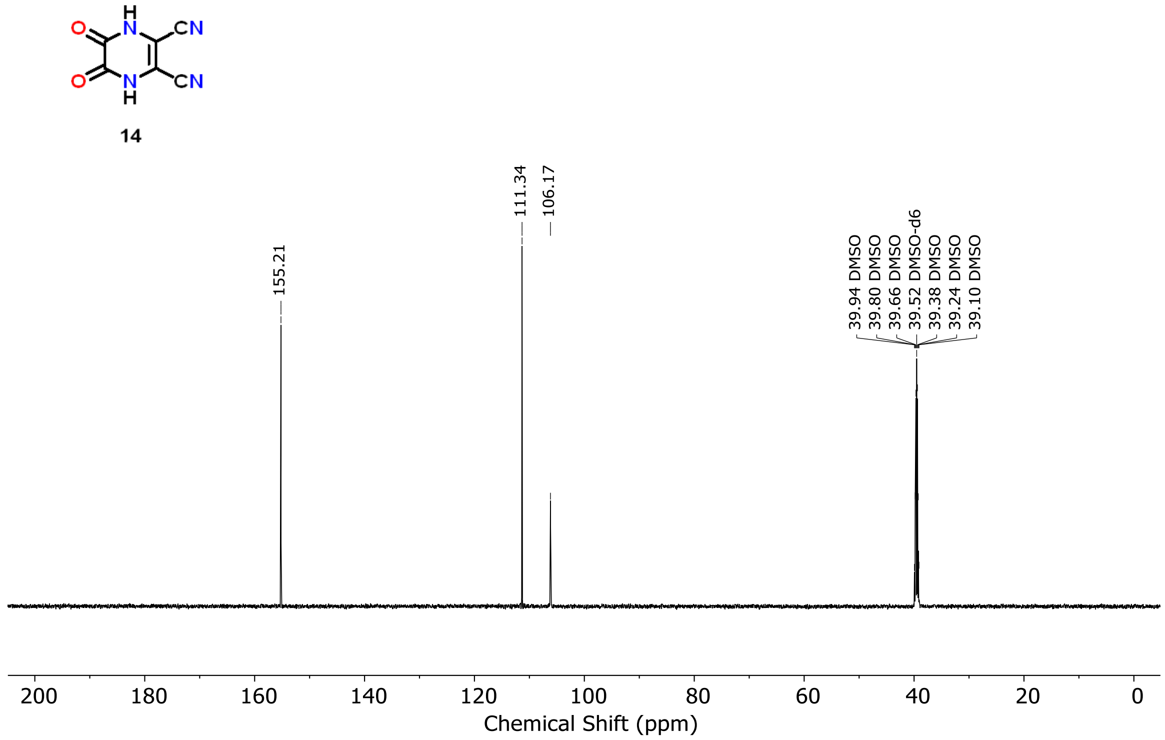

Figure S25. ^{13}C NMR spectrum of **14** in DMSO-d_6.

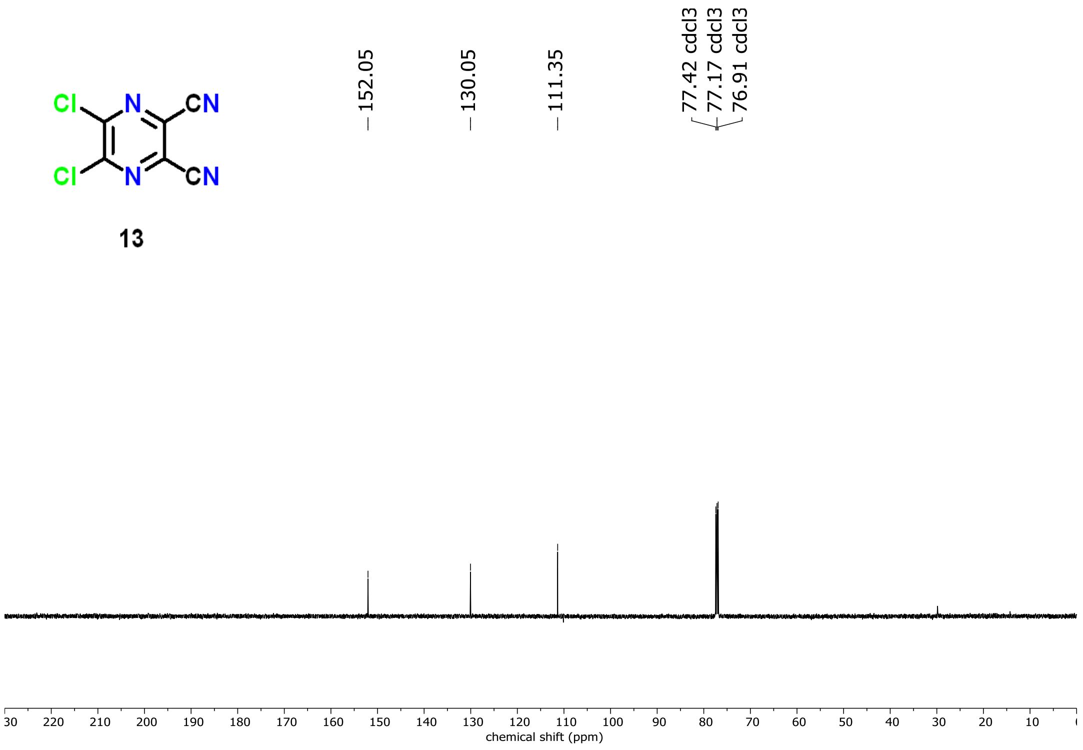

Figure S26. ^{13}C NMR spectrum of **13** in $CDCl_3$.

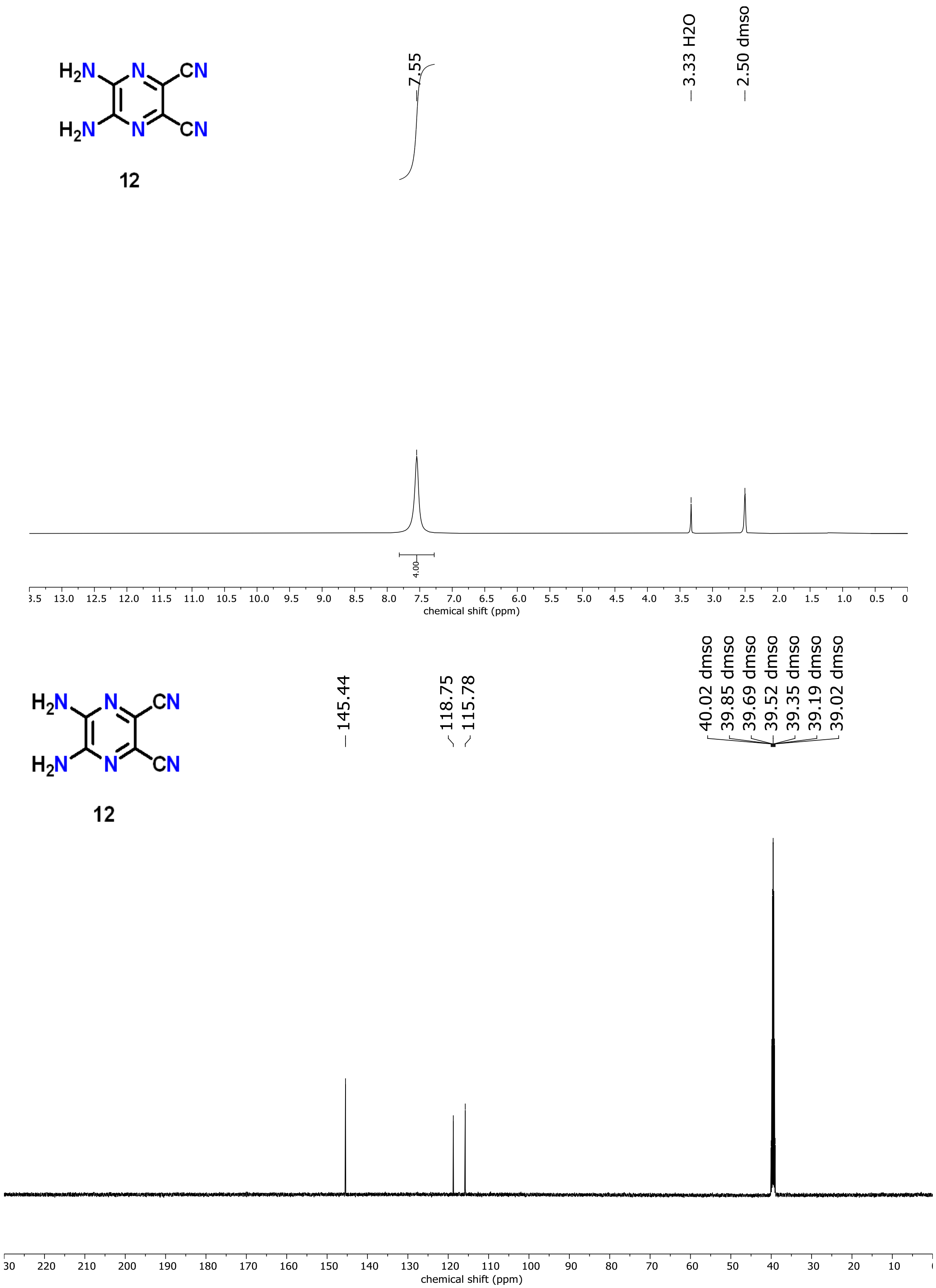

Figure S27. ^{1}H and ^{13}C NMR spectra of **12** in DMSO-d_6.

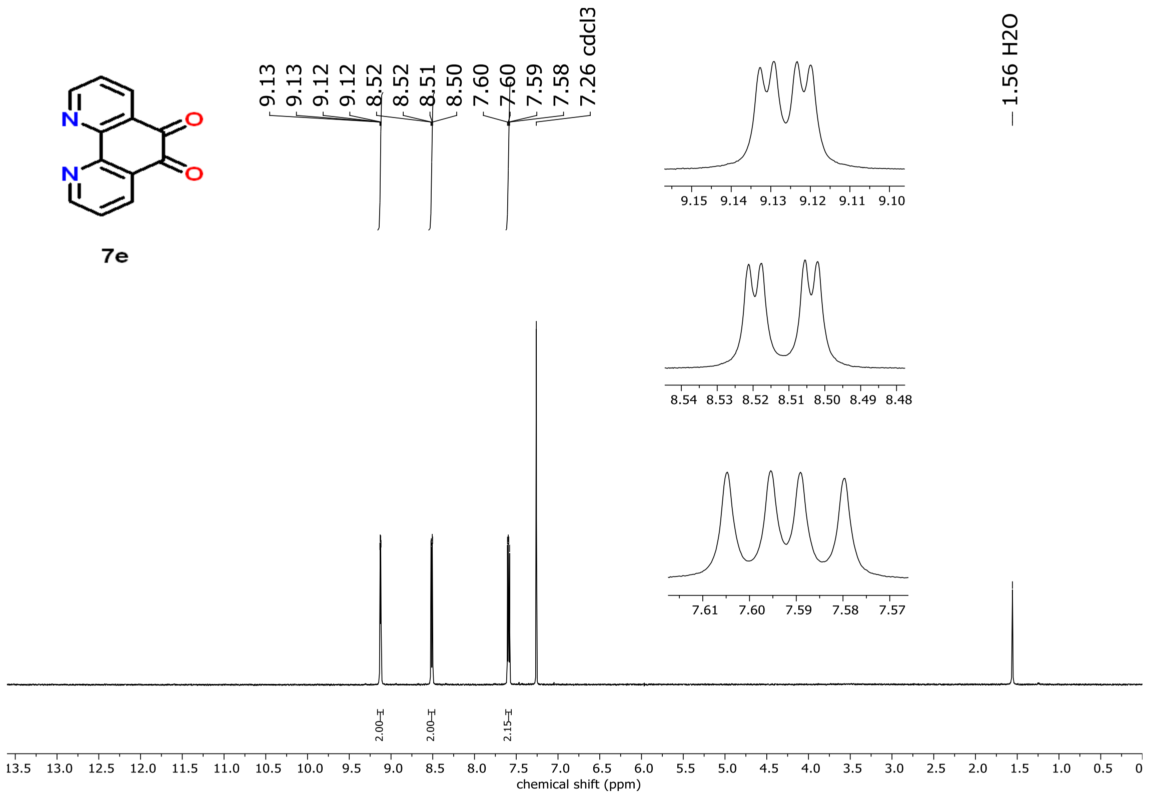

Figure S28. ^{1}H NMR spectrum of **7e** in $CDCl_3$

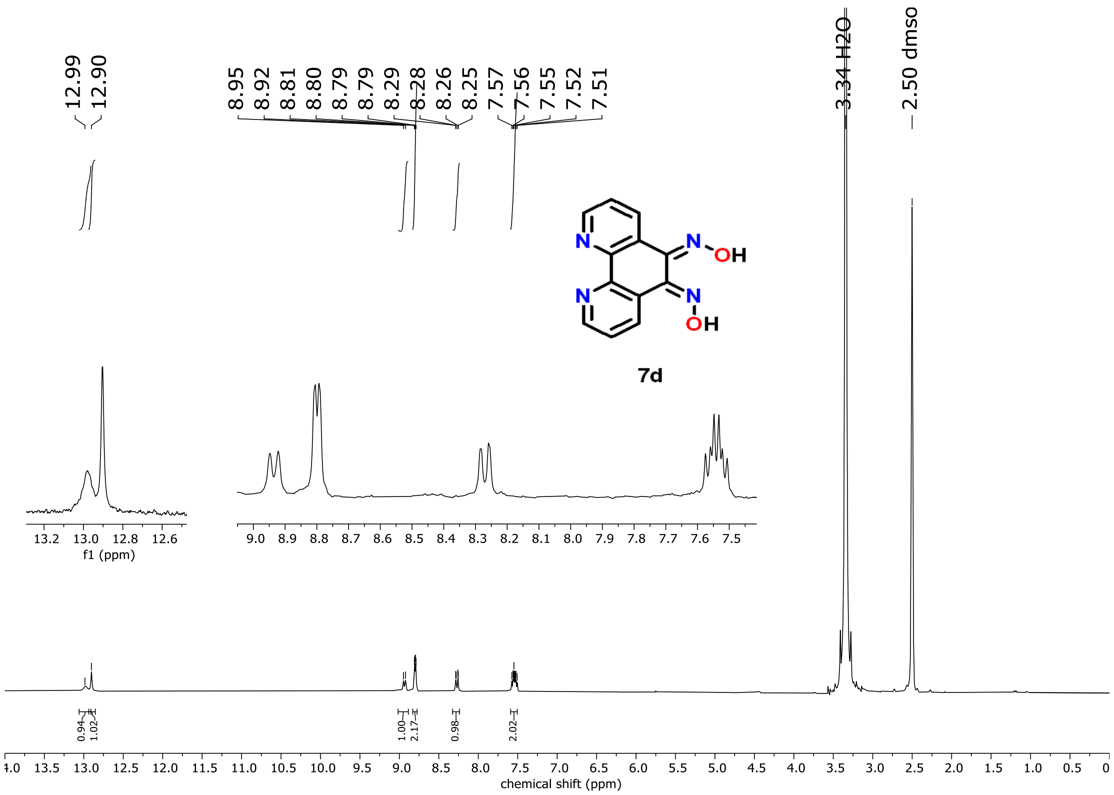

Figure S29. ^{1}H NMR spectrum of **7d** in DMSO-d_6.

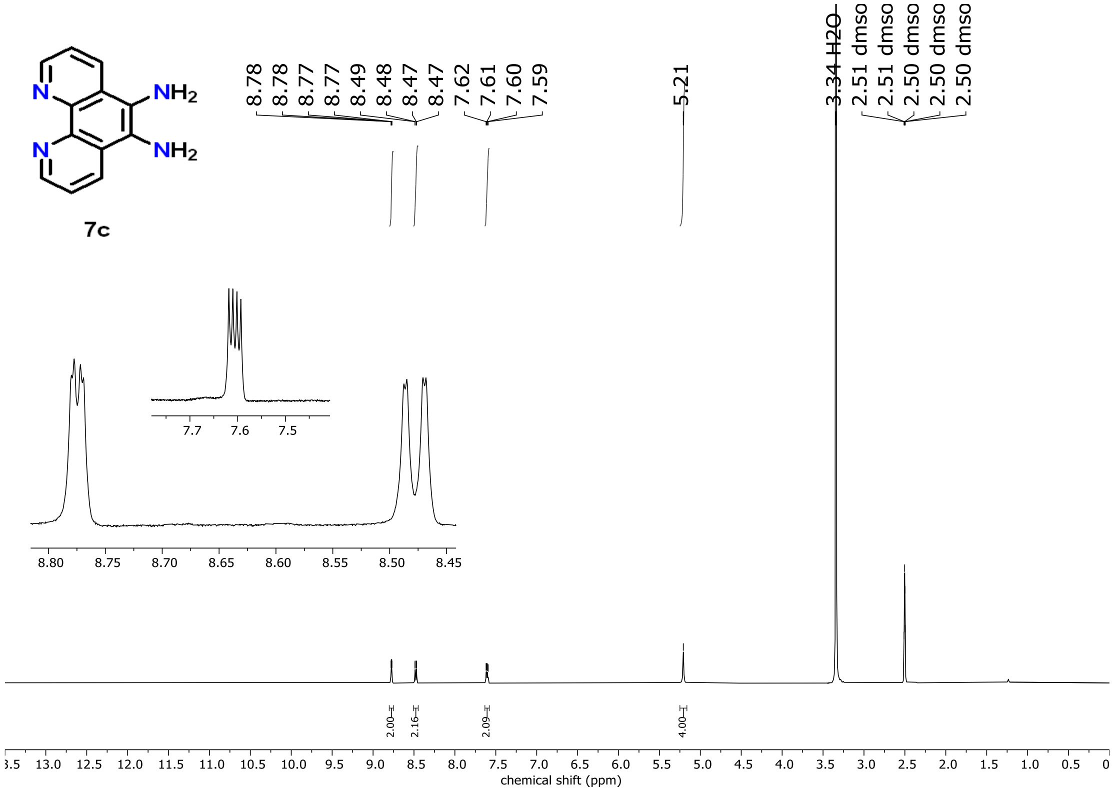

Figure S30. ^{1}H NMR spectrum of **7c** in DMSO-d_6.

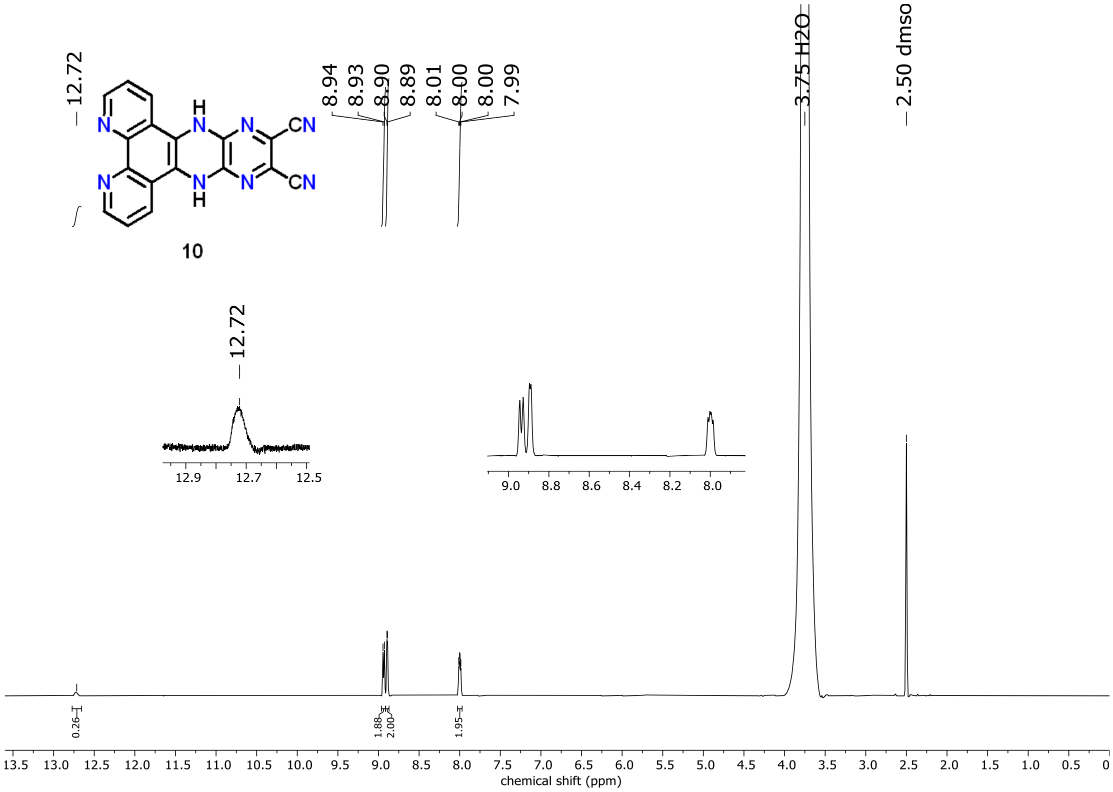

Figure S31. ^{1}H NMR spectrum of **10** in DMSO-d_6.

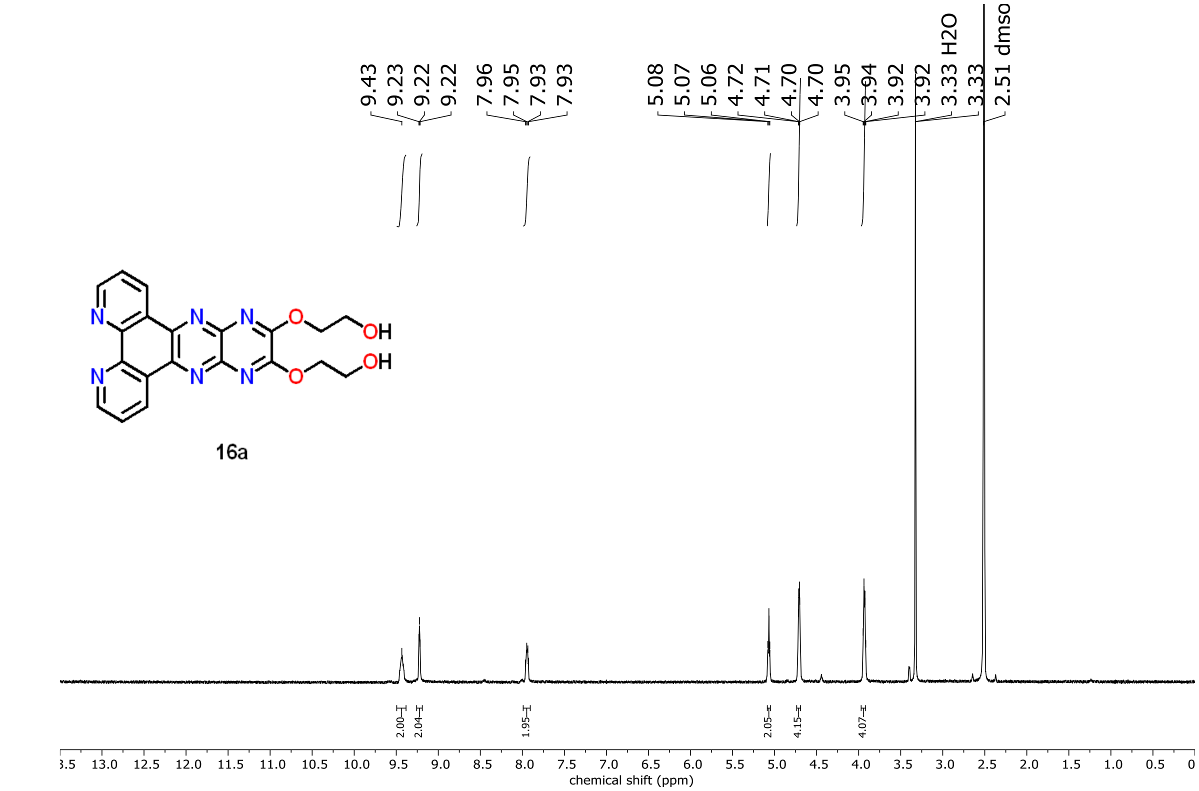

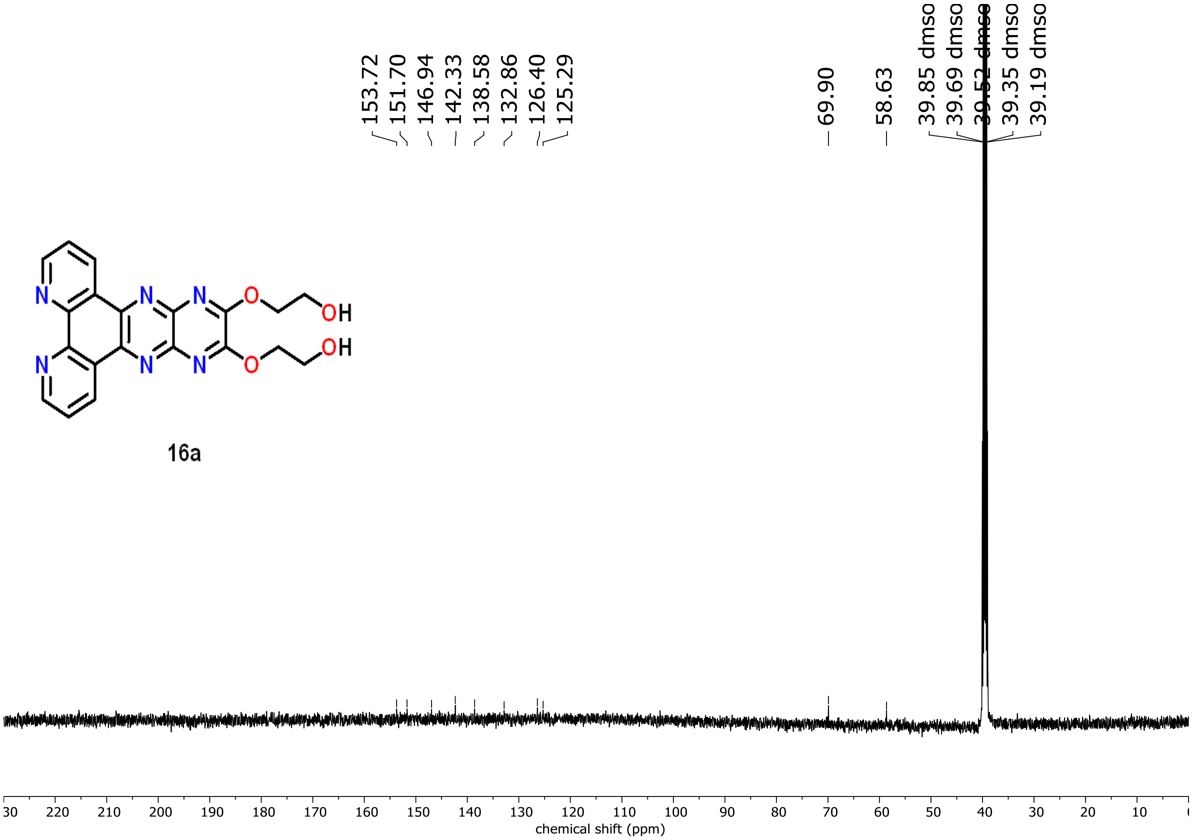

Figure S32. ^{1}H and ^{13}C NMR spectra of **16a** in DMSO-d_6.

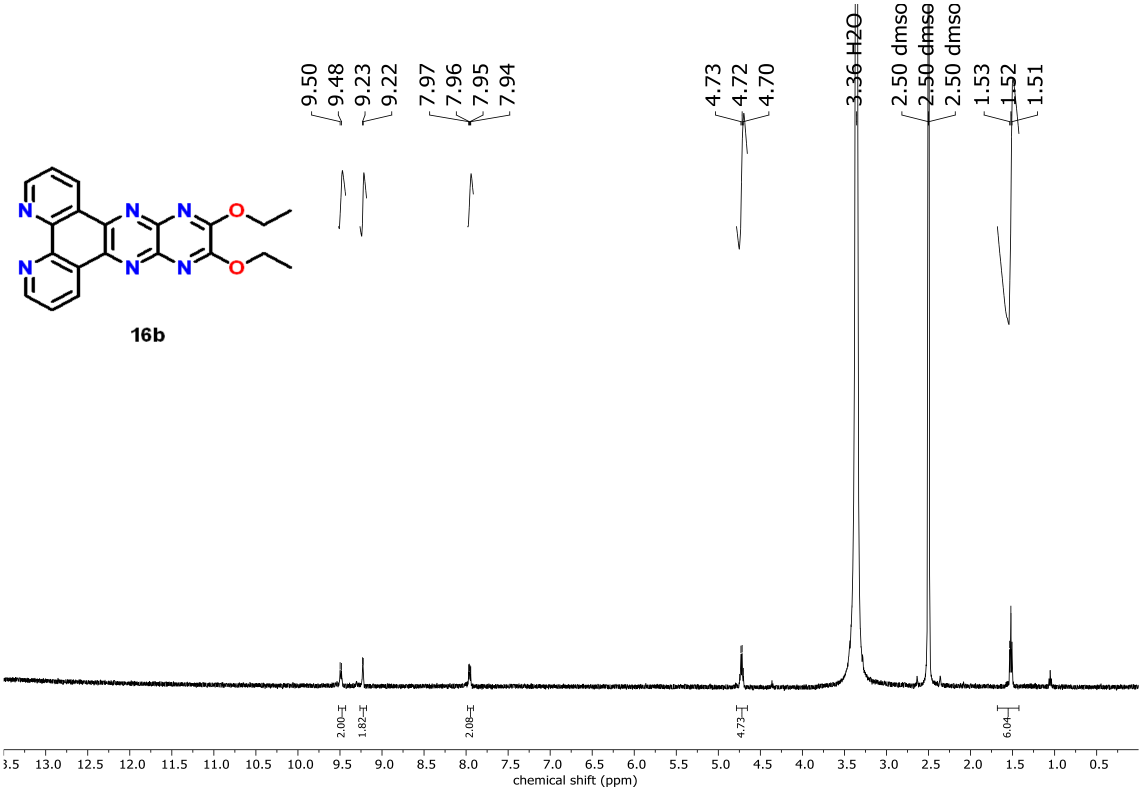

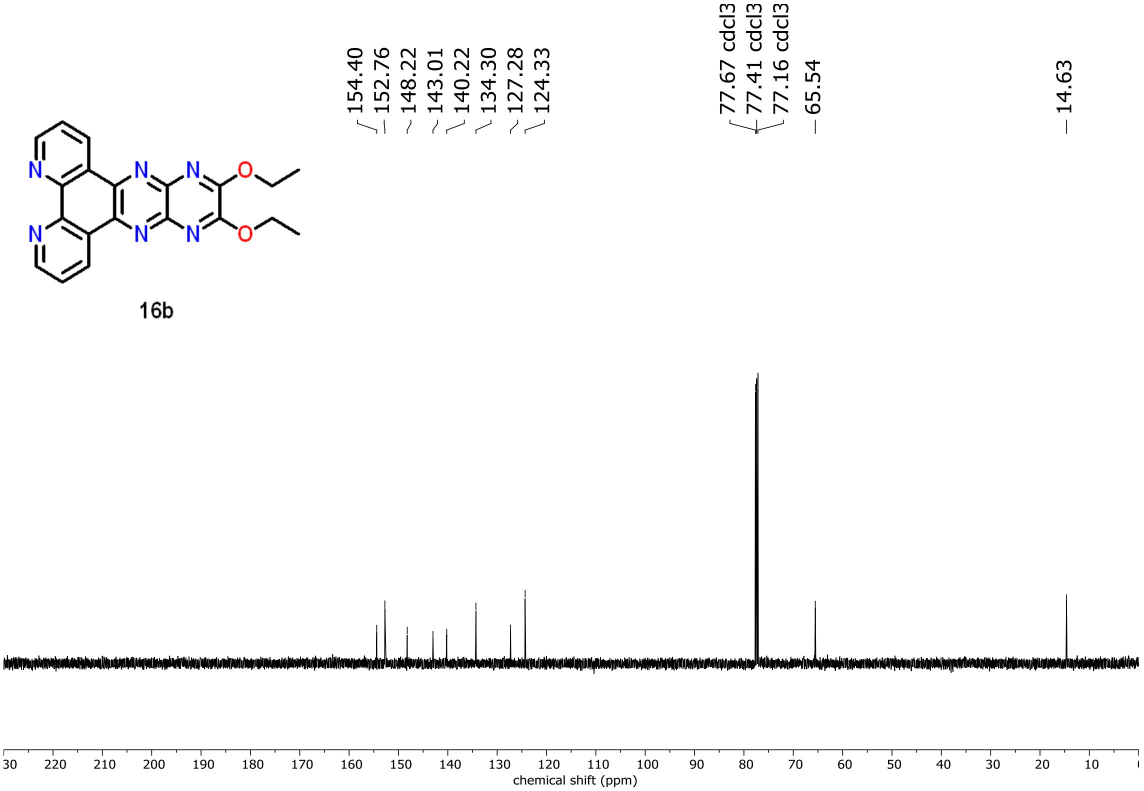

Figure S33. ^{1}H and ^{13}C NMR spectra of **16b** in DMSO-d_6 and $CDCl_3$, respectively.

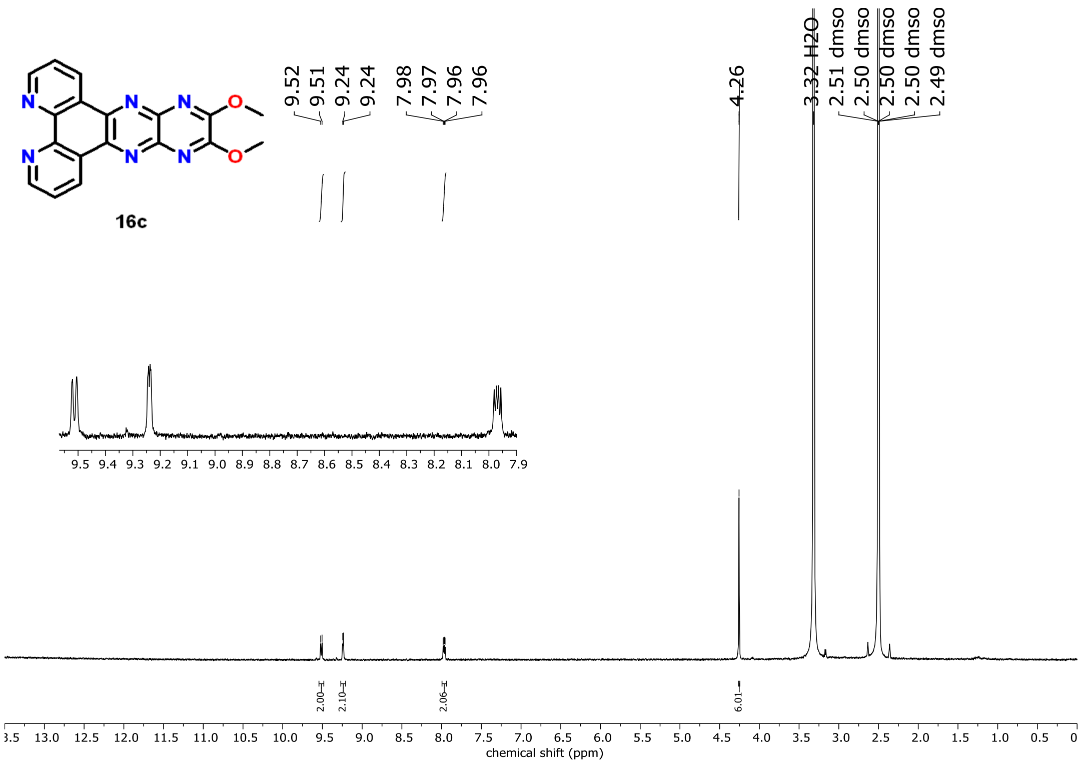

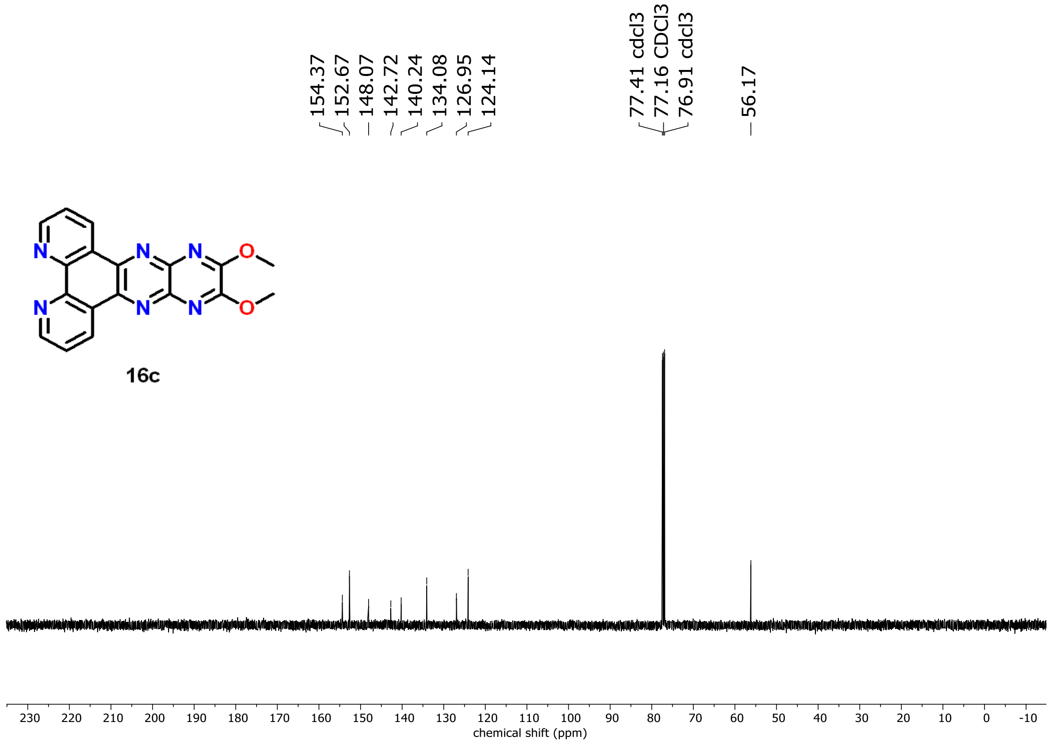

Figure S34. ^{1}H and ^{13}C NMR spectra of **16c** in DMSO-d_6 and $CDCl_3$, respectively.

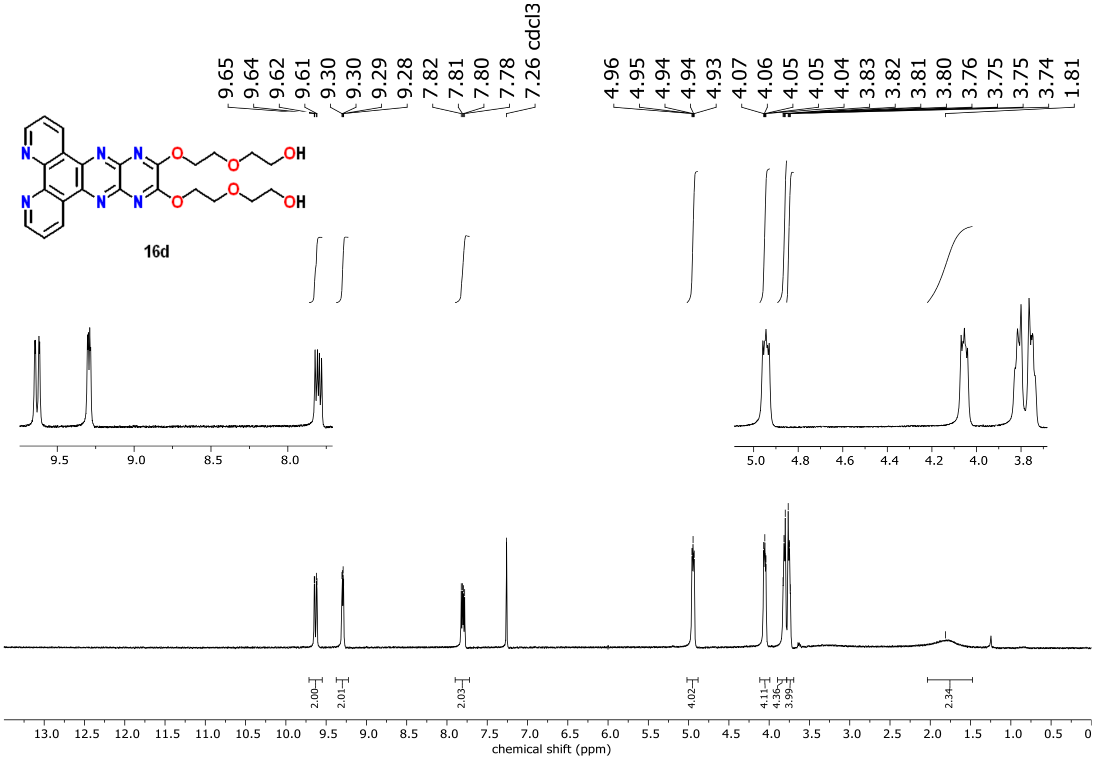

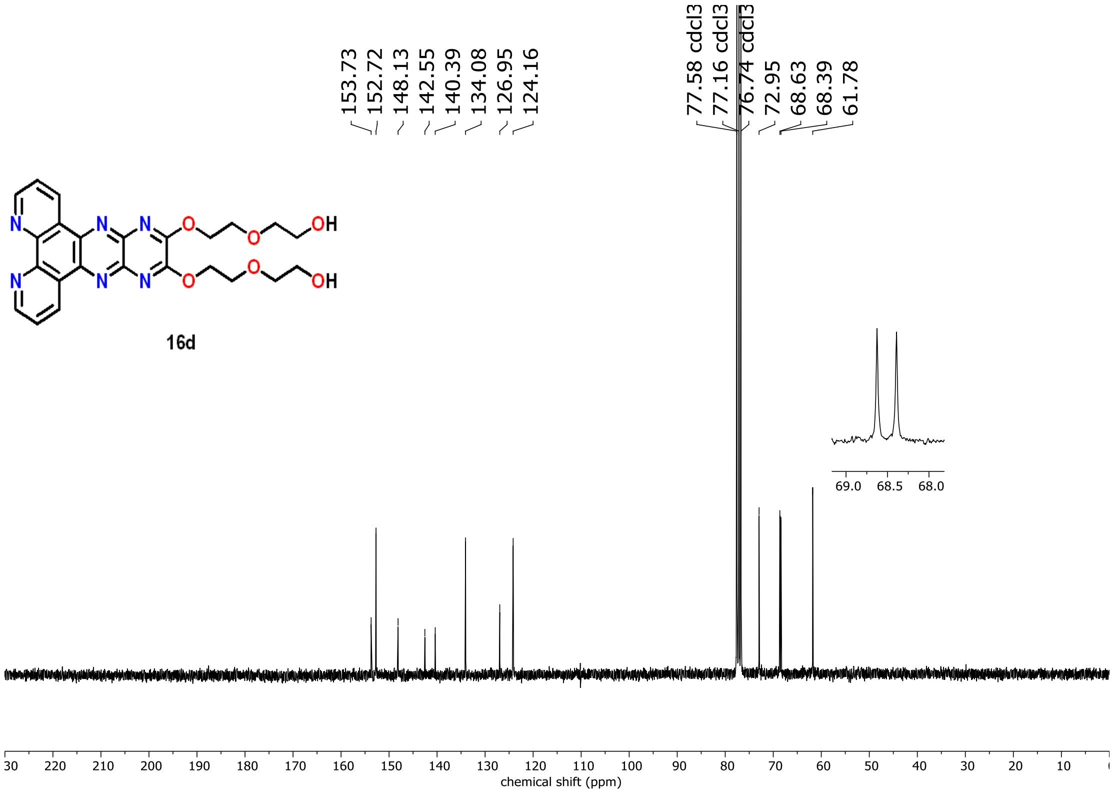

Figure S35. ^{1}H and ^{13}C NMR spectra of **16d** in $CDCl_3$.

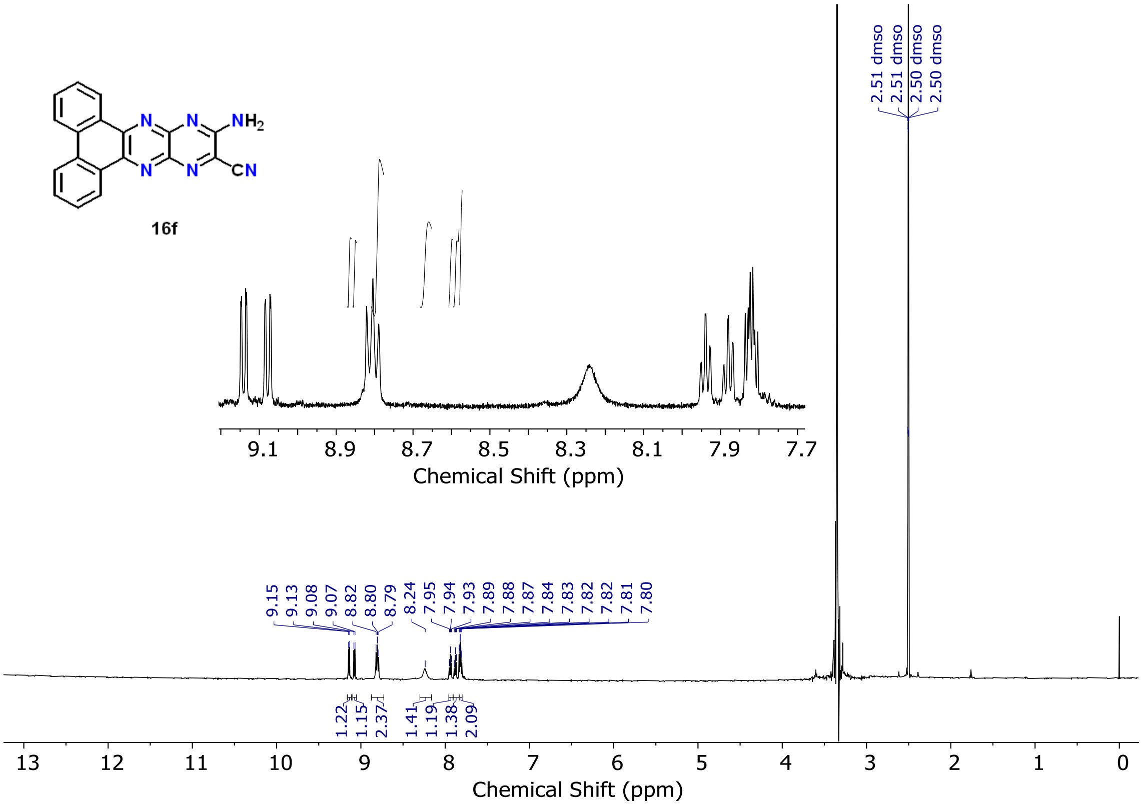

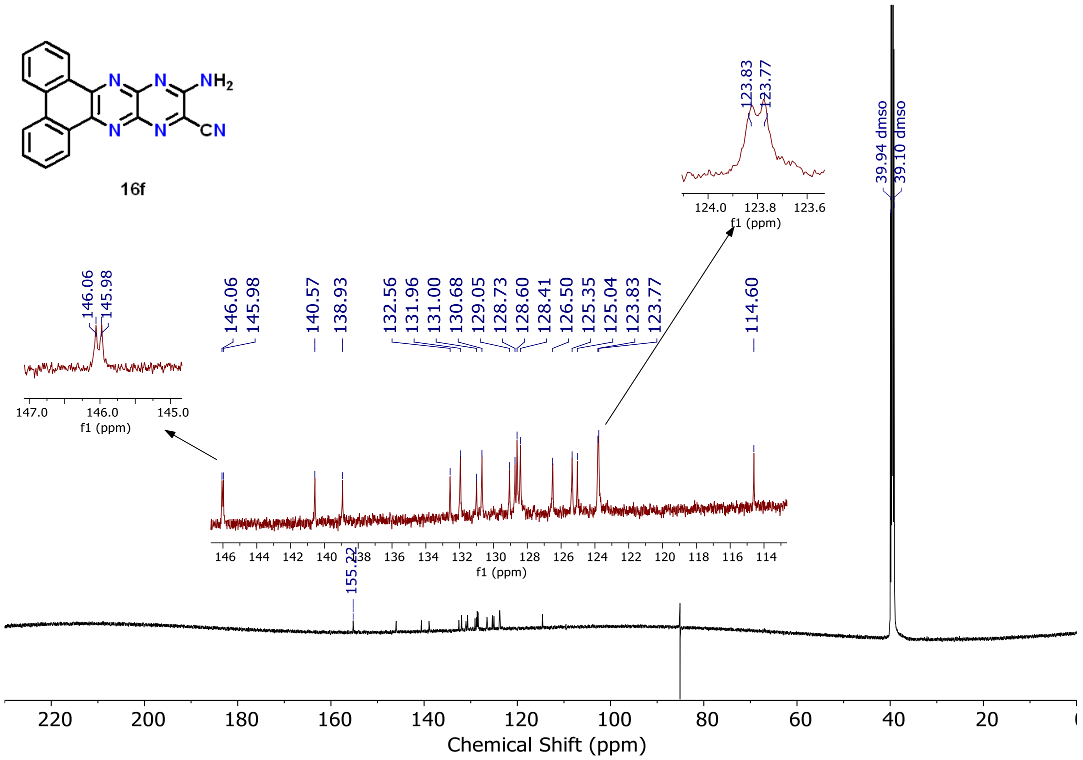

Figure S36. ^1H and ^{13}C NMR spectra of **16f** in $CDCl_3$.

UV-VISIBLE SPECTROSCOPY

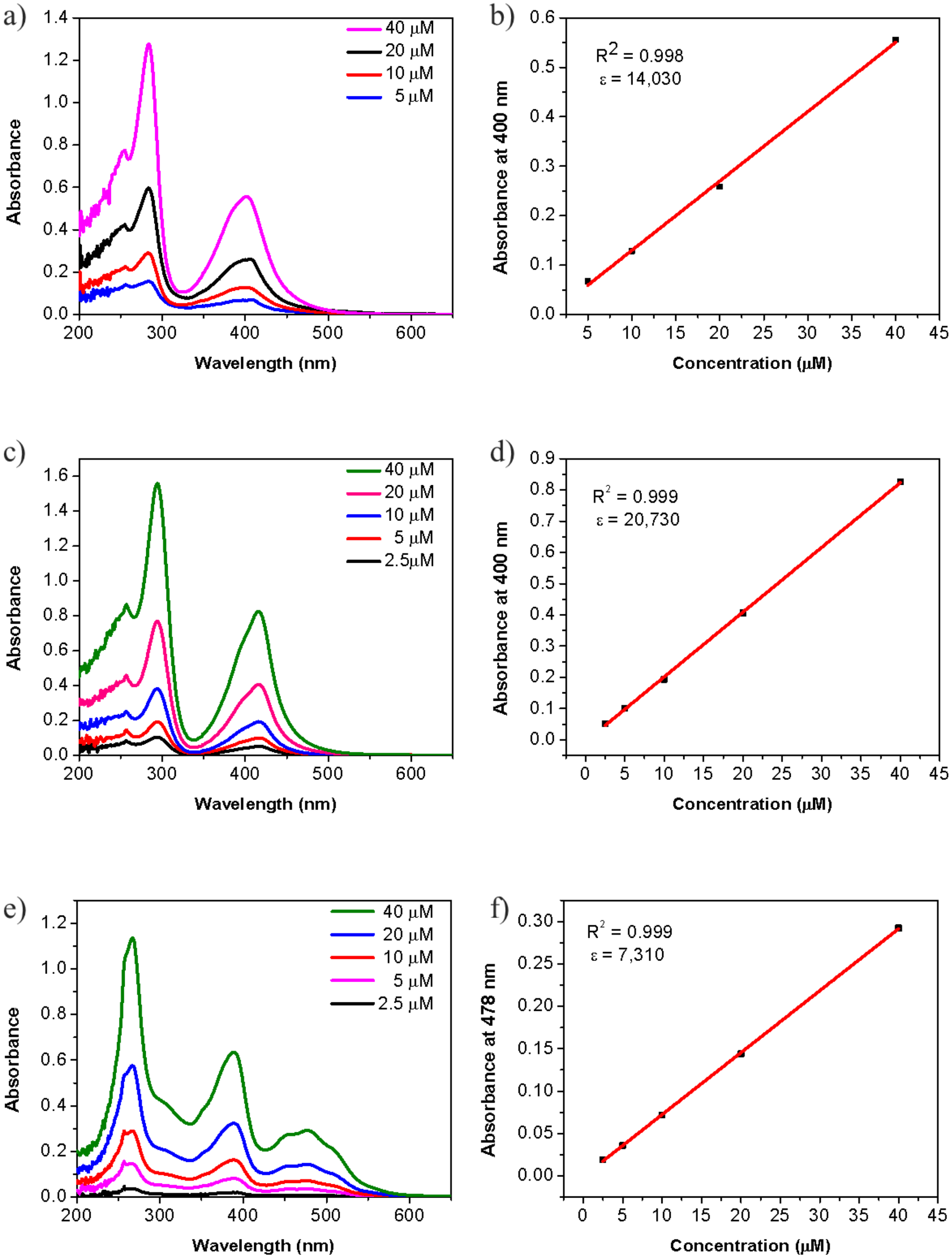

Figure S37. Absorbance spectra and Beer-Lambert plots for ab) **1a**; cd) **2a**; and ef) **3a**.

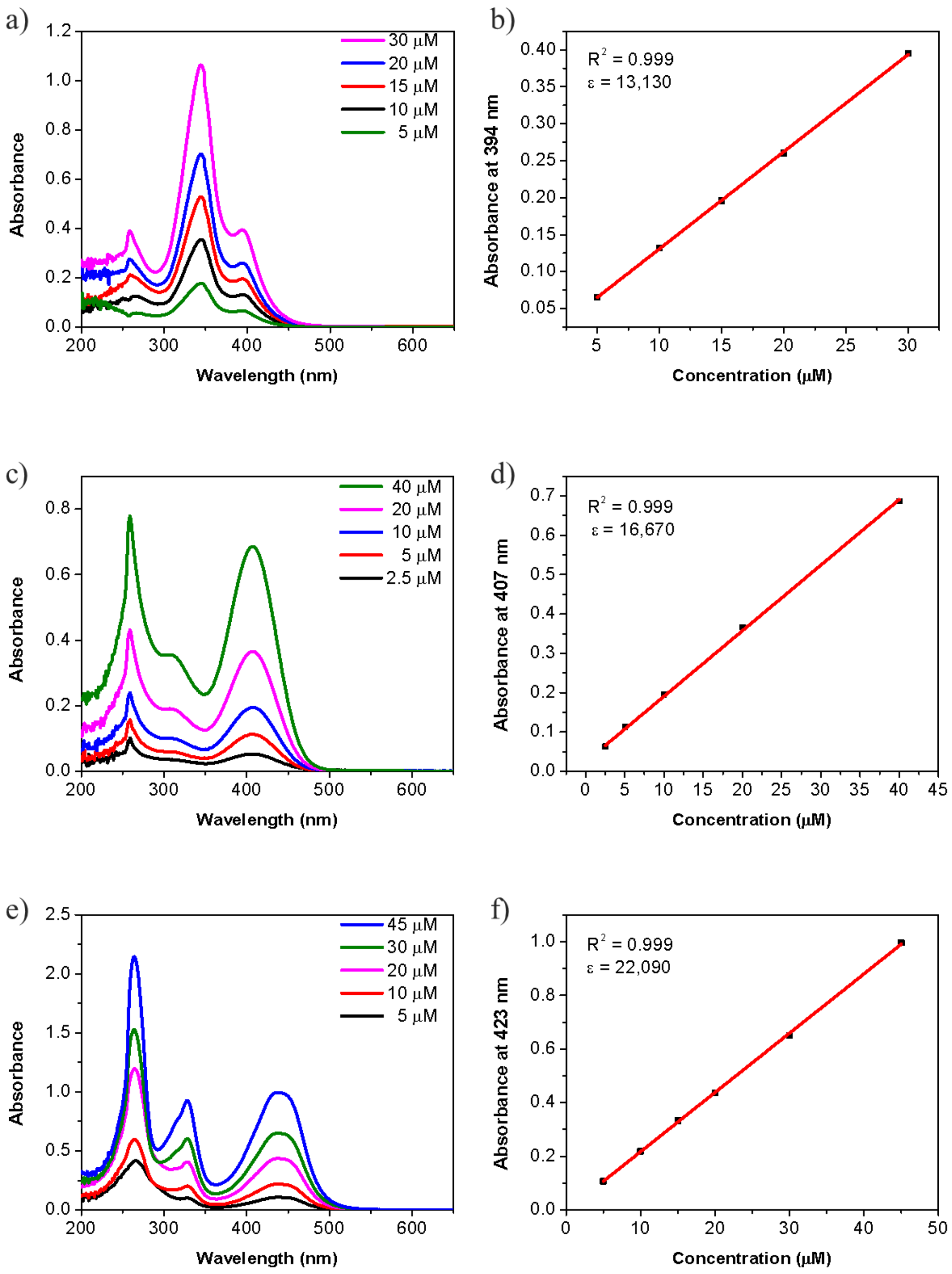

Figure S38. Absorbance spectra and Beer-Lambert plots for ab) **4a**; cd) **5a**; and ef) **6a**.

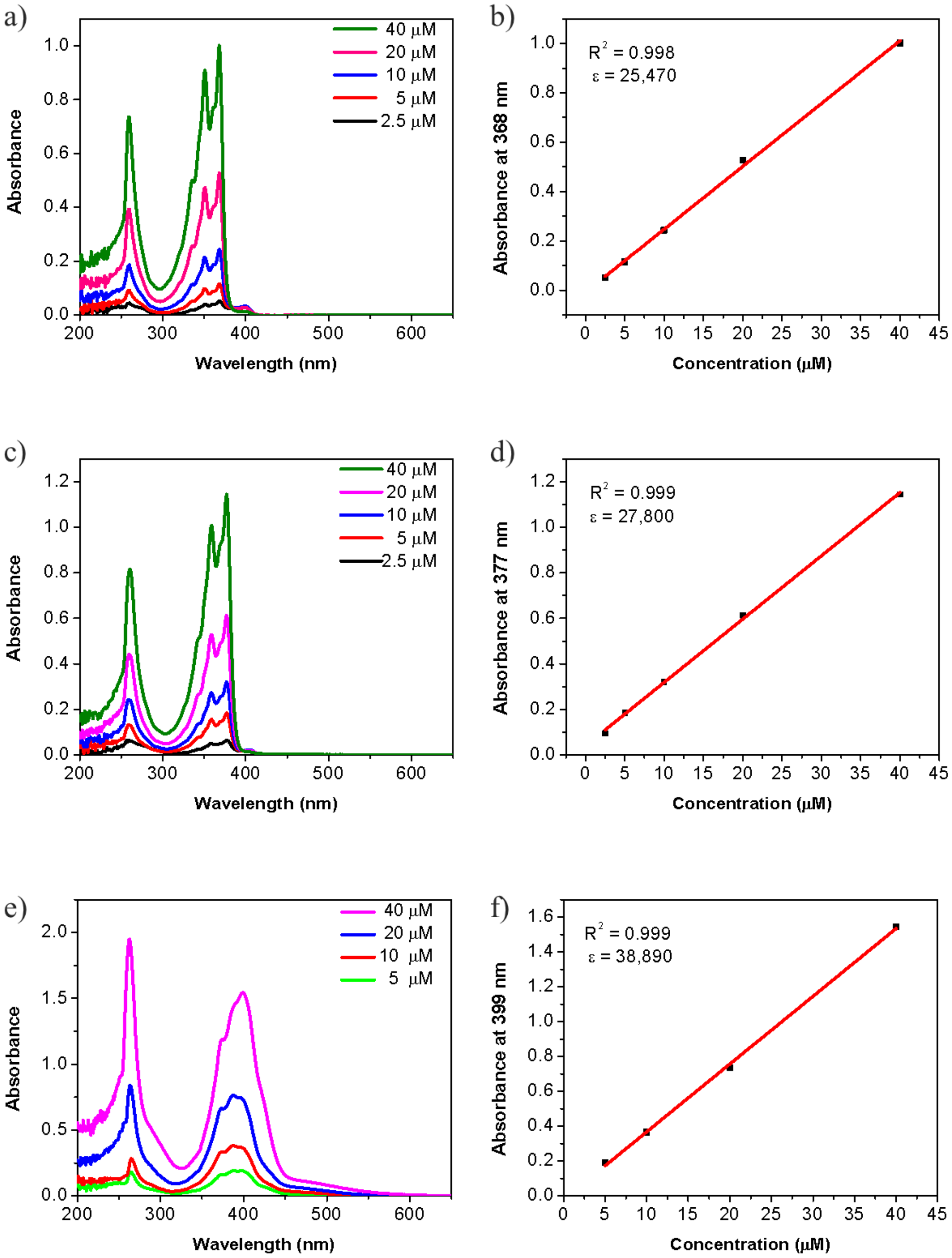

Figure S39. Absorbance spectra and Beer-Lambert plots for ab) **1b**; cd) **2b**; and ef) **3b**.

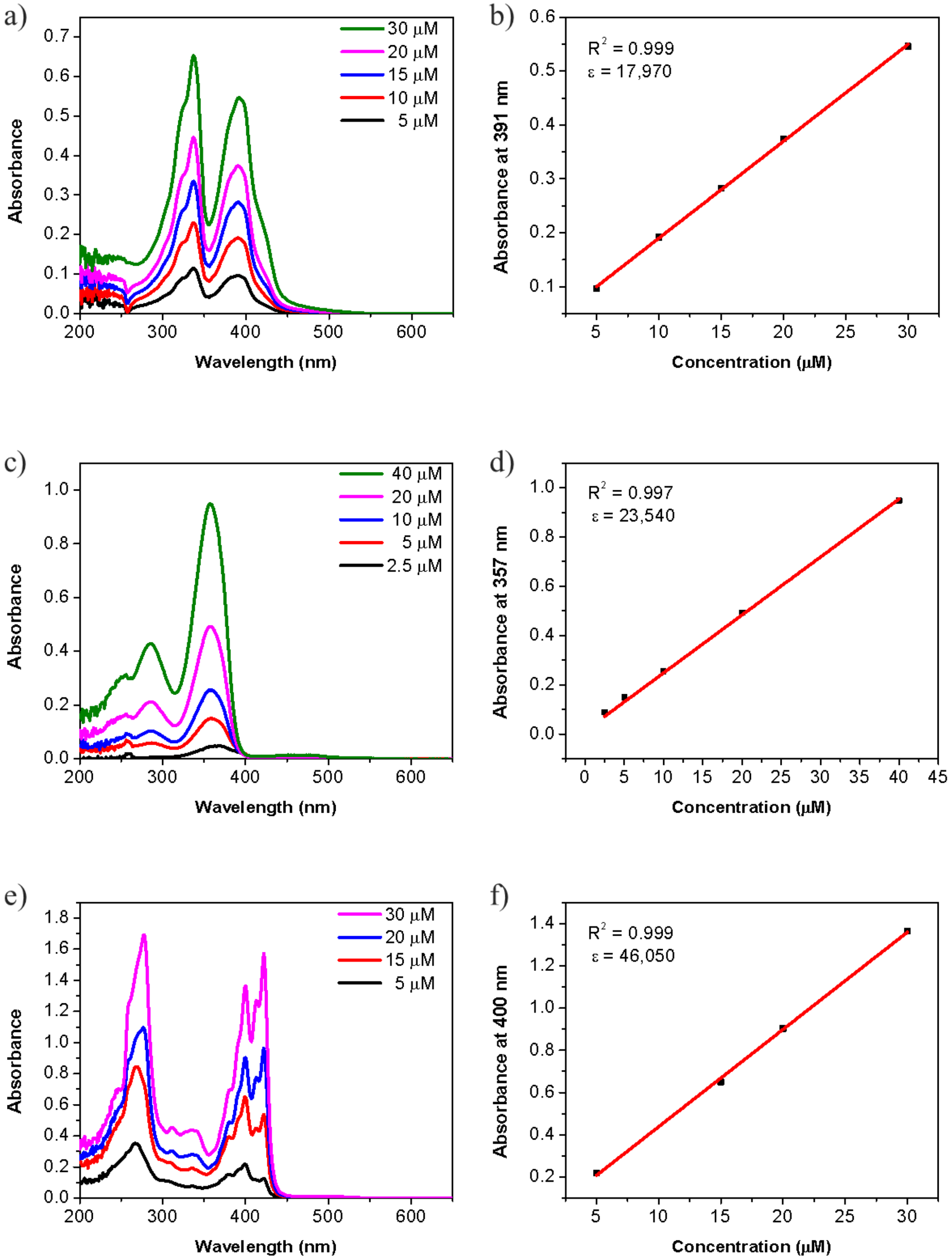

Figure S40. Absorbance spectra and Beer-Lambert plots for ab) **4b**; cd) **5b**; and ef) **6b**.

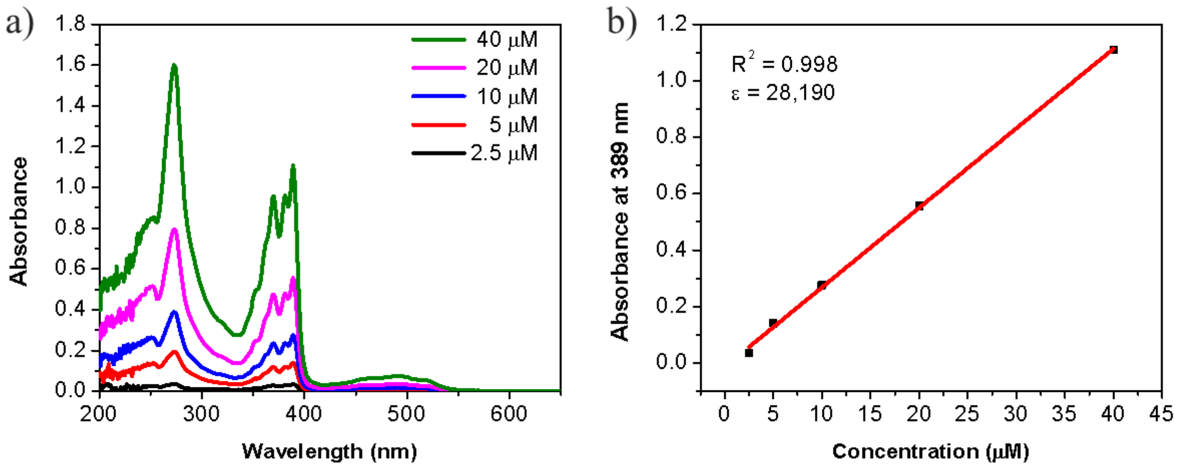

Figure S41. Absorbance spectra a) and Beer-Lambert plots b) for **7b**.

X-RAY DIFFRACTION

X-ray experimental of 6b: X-Ray intensity data were collected at 100 K on a Bruker **DUO** diffractometer using MoKα radiation (λ = 0.71073 Å) and an APEXII CCD area detector.

Raw data frames were read by the program SAINT and integrated using 3D profiling algorithms. The resulting data were reduced to produce hkl reflections and their intensities and estimated standard deviations. The data were corrected for Lorentz and polarization effects and numerical absorption corrections were applied based on indexed and measured faces.

The structure was solved and refined in *SHELXTL2014*, using full-matrix least-squares refinement. The non-H atoms were refined with anisotropic thermal parameters. All O-bound and N-bound protons were obtained from a Difference Fourier map and refined freely. All C-bound hydrogen atoms were calculated in idealized positions and refined riding on their parent atoms. The non-coordinated ethylene glycol molecule was disordered and was refined in two parts. Their site occupation factors were fixed to 0.600(2) and 0.400(2) for the major and minor parts, respectively. All protons of the disordered solvent molecule including those bound to the oxygen atoms were calculated in idealized positions. The nitrogen bound protons of the main molecule were obtained from a Difference Fourier maps and refined freely. In the final cycle of refinement, 3818 reflections (of which 1826 are observed with $I > 2\sigma(I)$) were used to refine 273 parameters and the resulting R_1, wR_2 and S (goodness of fit) were 5.70%, 11.42% and 0.914, respectively. The refinement was carried out by minimizing the wR_2 function using F^2 rather than F values. R_1 is calculated to provide a reference to the conventional R value but its function is not minimized.

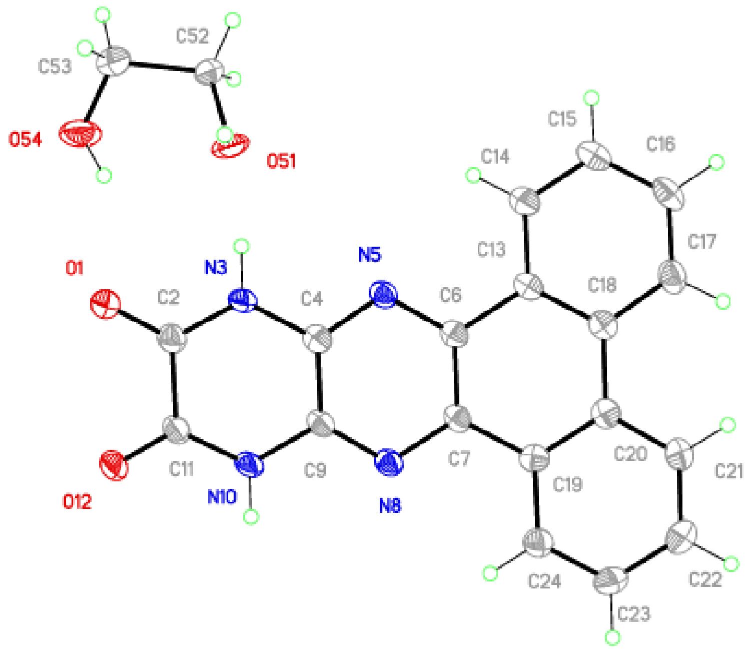

Figure S42. A representation of the structure is shown with thermal displacement ellipsoids at 50%. The ethylene glycol is disordered, and one disordered part is omitted for clarity.

Table S5. Crystal data and structure refinement for **6b**

Identification code	tural4	
CCDC Number	2324698	
Empirical formula	C20 H16 N4 O4	
Formula weight	376.37	
Temperature	100(2) K	
Wavelength	0.71073 Å	
Crystal system	Monoclinic	
Space group	$P2_1/n$	
Unit cell dimensions	a = 12.925(3) Å	α = 90°
	b = 7.4475(19) Å	β = 101.499(5)°
	c = 17.574(5) Å	γ = 90°
Volume	1657.8(7) $Å^3$	
Z	4	
Density (calculated)	1.508 mg/m^3	
Absorption coefficient	0.108 mm^{-1}	
F(000)	784	
Crystal size	0.455 x 0.145 x 0.014 mm^3	

Theta range for data collection	1.796 to 27.497°.
Index ranges	-16≤h≤16, -9≤k≤9, -22≤l≤22
Reflections collected	18003
Independent reflections	3818 [R(int) = 0.1244]
Completeness to theta = 25.242°	100.0 %
Absorption correction	Analytical
Max. and min. transmission	0.9988 and 0.9751
Refinement method	Full-matrix least-squares on F^2
Data / restraints / parameters	3818 / 240 / 273
Goodness-of-fit on F^2	0.914
Final R indices [I>2sigma(I)]	R1 = 0.0570, wR2 = 0.1142 [1826]
R indices (all data)	R1 = 0.1407, wR2 = 0.1370
Extinction coefficient	n/a
Largest diff. peak and hole	0.291 and -0.296 e.Å^{-3}

$R1 = \sum(||F_o| - |F_c||) / \sum|F_o|$ $wR2 = [\sum[w(F_o^2 - F_c^2)^2] / \sum[w(F_o^2)^2]]^{1/2}$

$S = [\sum[w(F_o^2 - F_c^2)^2] / (n-p)]^{1/2}$ $w= 1/[\sigma^2(F_o^2)+(m*p)^2+n*p]$, $p = [max(F_o^2,0)+ 2* F_c^2]/3$, m & n are constants.

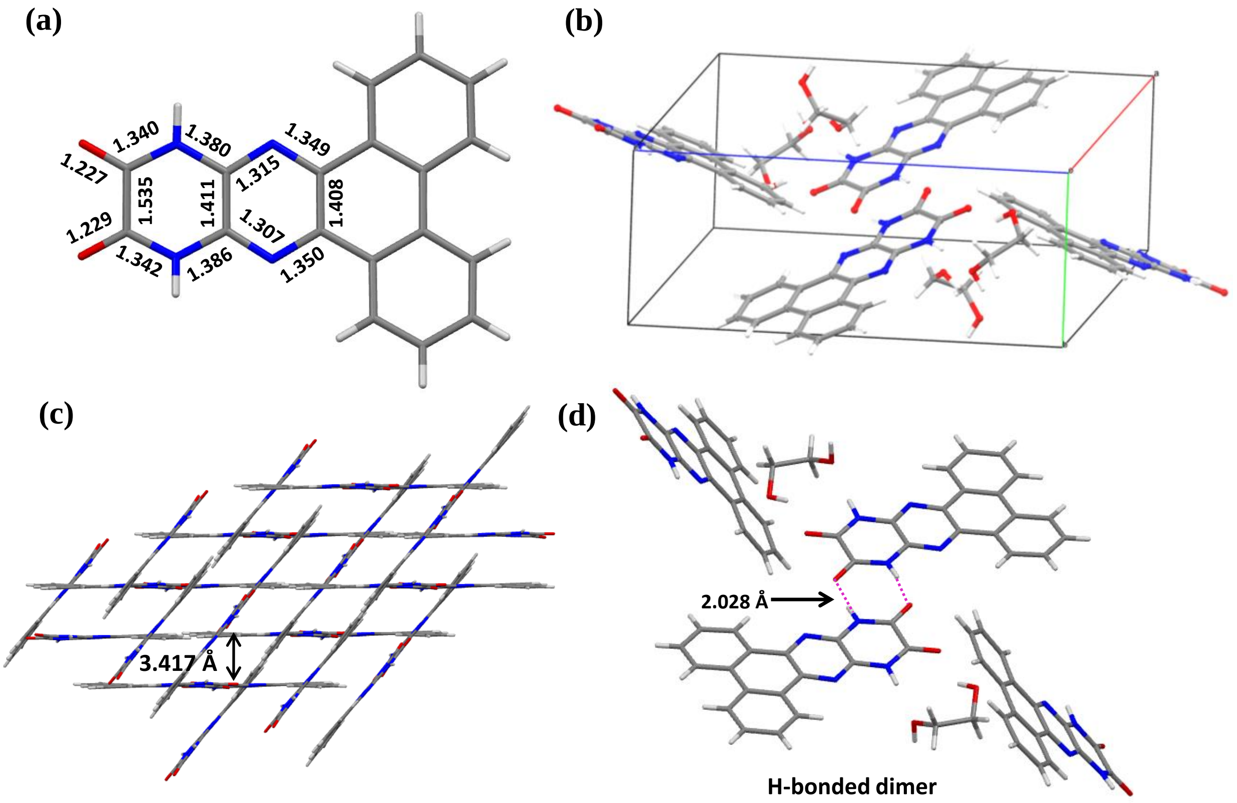

Figure S43. Asymmetric unit of **6b** with important bond lengths highlighted (a). Unit cell comprised of 4 molecules of **6b** (b). Close packing of **6b** showing the average distance between adjacent ring centroids (c). Packing diagram showing the average intermolecular N–H···O=C distance between **6b** molecules and solvent diethylene glycol molecules (d). Atom color code: blue N, gray C, red O, and white H, magenta H-bond.

Table S6. Crystal data and structure refinement for **5b**

Identification code	tural3	
CCDC Number	2324697	
Empirical formula	C18 H12 N4 O2	
Formula weight	316.32	
Temperature	100(2) K	
Wavelength	0.71073 Å	
Crystal system	Monoclinic	
Space group	$P2_1/c$	
Unit cell dimensions	a = 17.0068(19) Å	α = 90°.
	b = 7.2123(8) Å	β = 107.5554(19)°.
	c = 11.9963(14) Å	γ = 90°.
Volume	1402.9(3) $Å^3$	

Z	4
Density (calculated)	1.498 mg/m^3
Absorption coefficient	0.102 mm^{-1}
F(000)	656
Crystal size	0.434 x 0.130 x 0.052 mm^3
Theta range for data collection	2.512 to 27.500°.
Index ranges	$-22\leq h\leq 22$, $-9\leq k\leq 9$, $-15\leq l\leq 15$
Reflections collected	19520
Independent reflections	3220 [R(int) = 0.0254]
Completeness to theta = 25.242°	99.9 %
Absorption correction	Analytical
Max. and min. transmission	0.9959 and 0.9780
Refinement method	Full-matrix least-squares on F^2
Data / restraints / parameters	3220 / 195 / 225
Goodness-of-fit on F^2	1.089
Final R indices [I>2sigma(I)]	R1 = 0.0332, wR2 = 0.0916 [2727]
R indices (all data)	R1 = 0.0400, wR2 = 0.0953
Extinction coefficient	n/a
Largest diff. peak and hole	0.361 and -0.190 e.Å^{-3}

$R1 = \Sigma(||F_o| - |F_c||) / \Sigma|F_o|$ $wR2 = [\Sigma[w(F_o^2 - F_c^2)^2] / \Sigma[w(F_o^2)^2]]^{1/2}$

$S = [\Sigma[w(F_o^2 - F_c^2)^2] / (n-p)]^{1/2}$ $w= 1/[\sigma^2(F_o^2)+(m*p)^2+n*p]$, $p = R[max(F_o^2,0)+ 2* F_c^2]/3$, m & n are constants.

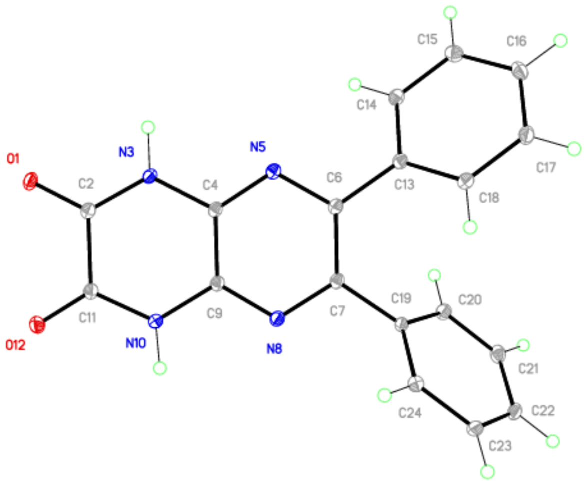

Figure S44. ORTEP of the asymmetric unit of **5b** at 50% probability level.

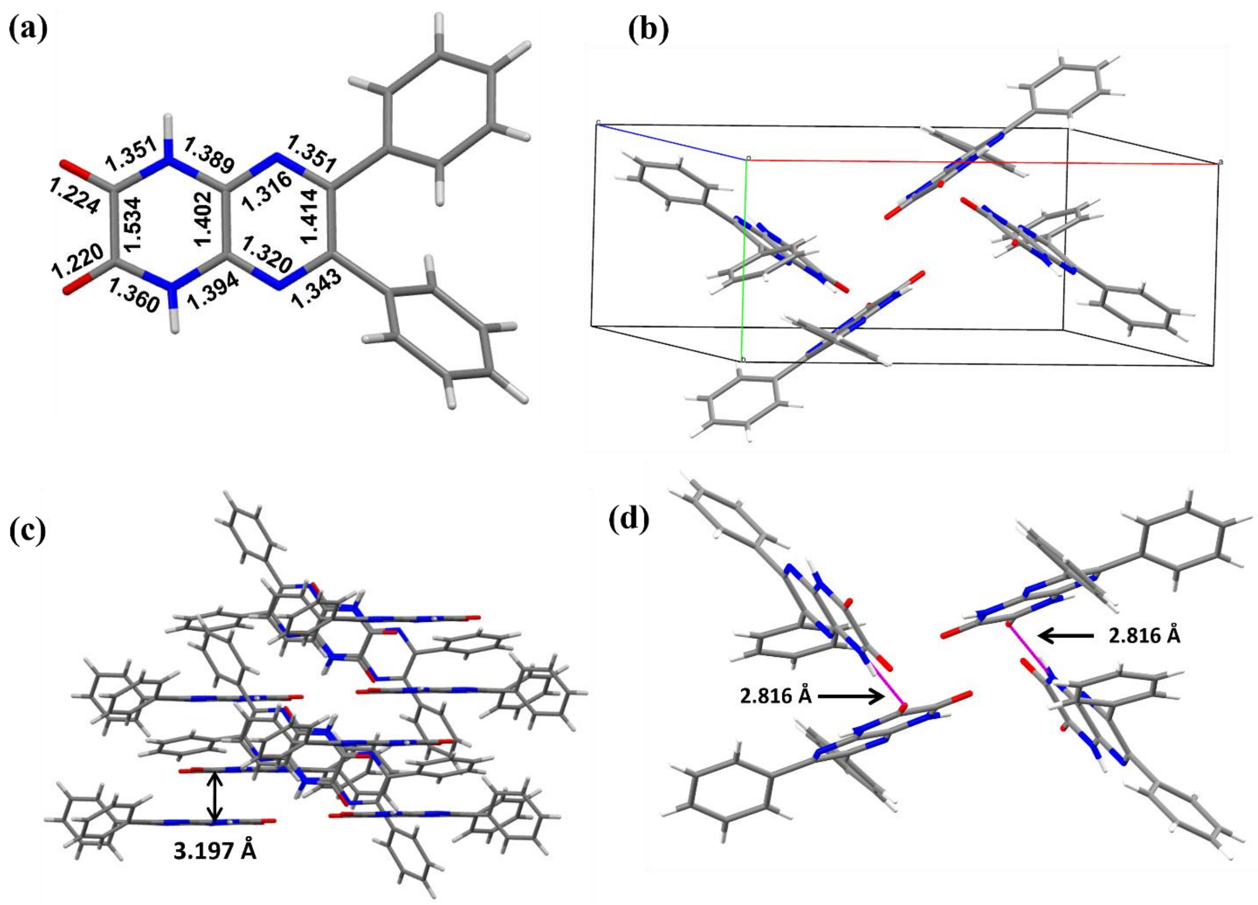

Figure S45. Asymmetric unit of **5b** with important bond lengths highlighted (a). Unit cell comprised of 4 molecules of **5b** (b). Close packing of **5b** showing the average distance between adjacent ring centroids (c). Packing diagram showing the average intermolecular N–H···O=C distance between **6b** molecules (d). Atom color code: blue N, gray C, red O, and white H, magenta H-bond.

Table S7. Crystal data and structure refinement for **2b**

Identification code	tural5	
CCDC Number	2324699	
Empirical formula	C12 H12 N4 O2	
Formula weight	244.26	
Temperature	100(2) K	
Wavelength	0.71073 Å	
Crystal system	Monoclinic	
Space group	$P2_1/n$	
Unit cell dimensions	a = 11.084(2) Å	α = 90°.
	b = 4.4996(10) Å	β = 105.040(4)°.

	c = 21.335(5) Å	γ = 90°.
Volume	1027.6(4) Å^3	
Z	4	
Density (calculated)	1.579 Mg/m^3	
Absorption coefficient	0.112 mm^{-1}	
F(000)	512	
Crystal size	0.202 x 0.101 x 0.056 mm^3	
Theta range for data collection	1.902 to 27.493°.	
Index ranges	-14≤h≤14, -5≤k≤5, -27≤l≤27	
Reflections collected	15752	
Independent reflections	2517 [R(int) = 0.0400]	
Completeness to theta = 25.242°	100.0 %	
Absorption correction	Analytical	
Max. and min. transmission	0.9965 and 0.9856	
Refinement method	Full-matrix least-squares on F^2	
Data / restraints / parameters	2517 / 150 / 174	
Goodness-of-fit on F^2	0.987	
Final R indices [I>2sigma(I)]	R1 = 0.0324, wR2 = 0.0752 [2043]	
R indices (all data)	R1 = 0.0452, wR2 = 0.0789	
Extinction coefficient	n/a	
Largest diff. peak and hole	0.260 and -0.195 e.Å^{-3}	

$R1 = \Sigma(||F_o| - |F_c||) / \Sigma|F_o|$ $wR2 = [\Sigma[w(F_o^2 - F_c^2)^2] / \Sigma[w(F_o^2)^2]]^{1/2}$

$S = [\Sigma[w(F_o^2 - F_c^2)^2] / (n-p)]^{1/2}$ $w= 1/[\sigma^2(F_o^2)+(m*p)^2+n*p]$, $p = [max(F_o^2,0)+ 2* F_c^2]/3$, m & n are constants.

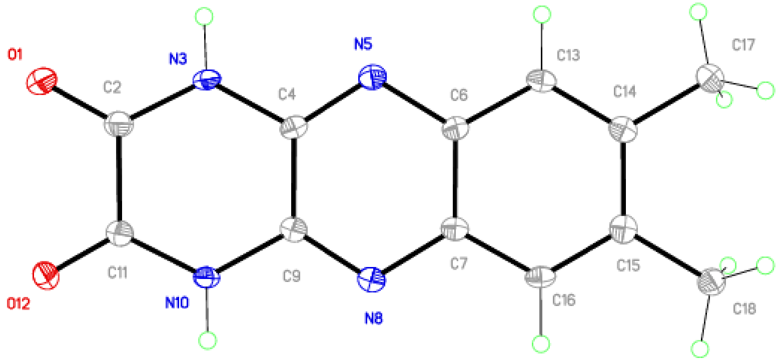

Figure S46. A representation of the molecule **2b** shown using thermal displacement ellipsoids at 50%.

REFERENCES

1. MØrkved, E. H.; Holmaas, L. T.; KjØsen, H.; Hvistendahl, G., Preparation of Magnesium Azaphthalocyanines by Cyclotetramerisation of *S*-Substituted 4,5-Disulfanylpyrazine-2,3,dicarbonitriles. *Acta Chemica Scandinavica* **1996,** *50*, 1153-1156.
2. Bureš, F.; Čermáková, H.; Kulhánek, J.; Ludwig, M.; Kuznik, W.; Kityk, I. V.; Mikysek, T.; Růžička, A., Structure–Property Relationships and Nonlinear Optical Effects in Donor-Substituted Dicyanopyrazine-Derived Push–Pull Chromophores with Enlarged and Varied π-Linkers. *European Journal of Organic Chemistry* **2012,** *2012* (3), 529-538.
3. Ried, W.; Tsiotis, G., Neue kondensierte Stickstoff-reiche Heterocyclen aus 5,6-Dichlorpyrazin-2,3-dicarbonitril. *Liebigs Annalen der Chemie* **1988,** *1988* (12), 1197-1199.
4. Wang, C.; Lystrom, L.; Yin, H.; Hetu, M.; Kilina, S.; McFarland, S. A.; Sun, W., Increasing the triplet lifetime and extending the ground-state absorption of biscyclometalated Ir(iii) complexes for reverse saturable absorption and photodynamic therapy applications. *Dalton Transactions* **2016,** *45* (41), 16366-16378.
5. Irudaya Jothi, A.; Alexander, V., Organic NLO material with H-bonded 1D helical self-assembly: synthesis, X-ray crystal structure, DFT calculations, SHG measurements and thermal studies of (5Z,6E)-1,10-phenanthroline-5,6-dione dioxime. *CrystEngComm* **2017,** *19* (35), 5251-5258.
6. Alrefai, R.; Hörner, G.; Schubert, H.; Berkefeld, A., Broadly versus Barely Variable Complex Chromophores of Planar Nickel(II) from κ3-N,N′,C and κ3-N,N′,O Donor Platforms. *Organometallics* **2021,** *40* (8), 1163-1177.
7. Thompson, B. C.; Kim, Y.-G.; McCarley, T. D.; Reynolds, J. R., Soluble Narrow Band Gap and Blue Propylenedioxythiophene-Cyanovinylene Polymers as Multifunctional Materials for Photovoltaic and Electrochromic Applications. *Journal of the American Chemical Society* **2006,** *128* (39), 12714-12725.
8. Richards, G. J.; Hill, J. P.; Subbaiyan, N. K.; D'Souza, F.; Karr, P. A.; Elsegood, M. R. J.; Teat, S. J.; Mori, T.; Ariga, K., Pyrazinacenes: Aza Analogues of Acenes. *The Journal of Organic Chemistry* **2009,** *74* (23), 8914-8923.
